# Simultaneous enhancement of stretchability, healability and carrier mobility in polymer semiconductors via hierarchical hydrogen-bonded engineering

**DOI:** 10.1093/nsr/nwag162

**Published:** 2026-03-14

**Authors:** Haoguo Yue, Ying Wang, Zhihao Meng, Shaochuan Luo, Jiasen Quan, Ruiwei Zhou, Haonan Geng, Xinyu Xia, Junxuan Tu, Jun Jin, Dongshan Zhou, Lei Zhang, Yonggang Zhen, Wenping Hu

**Affiliations:** State Key Laboratory of Organic-Inorganic Composites, College of Materials Science and Engineering, Beijing University of Chemical Technology, Beijing 100029, China; Shandong Key Laboratory of Chemical Energy Storage and New Battery Technology, School of Materials Science and Engineering, Liaocheng University, Liaocheng 252000, China; State Key Laboratory of Organic-Inorganic Composites, College of Materials Science and Engineering, Beijing University of Chemical Technology, Beijing 100029, China; State Key Laboratory of Smart Sensing Materials, MOE Key Laboratory of Organic Integrated Circuits & Tianjin Key Laboratory of Molecular Optoelectronic Sciences, Department of Chemistry, School of Sciences, Tianjin University, Tianjin 300072, China; State Key Laboratory of Organic-Inorganic Composites, College of Materials Science and Engineering, Beijing University of Chemical Technology, Beijing 100029, China; School of Chemistry and Chemical Engineering, Nanjing University, Nanjing 210023, China; State Key Laboratory of Organic-Inorganic Composites, College of Materials Science and Engineering, Beijing University of Chemical Technology, Beijing 100029, China; State Key Laboratory of Organic-Inorganic Composites, College of Materials Science and Engineering, Beijing University of Chemical Technology, Beijing 100029, China; State Key Laboratory of Organic-Inorganic Composites, College of Materials Science and Engineering, Beijing University of Chemical Technology, Beijing 100029, China; State Key Laboratory of Organic-Inorganic Composites, College of Materials Science and Engineering, Beijing University of Chemical Technology, Beijing 100029, China; State Key Laboratory of Organic-Inorganic Composites, College of Materials Science and Engineering, Beijing University of Chemical Technology, Beijing 100029, China; State Key Laboratory of Smart Sensing Materials, MOE Key Laboratory of Organic Integrated Circuits & Tianjin Key Laboratory of Molecular Optoelectronic Sciences, Department of Chemistry, School of Sciences, Tianjin University, Tianjin 300072, China; State Key Laboratory of Organic-Inorganic Composites, College of Materials Science and Engineering, Beijing University of Chemical Technology, Beijing 100029, China; State Key Laboratory of Fine Chemicals, School of Chemical Engineering, Dalian University of Technology, Dalian 116024, China; School of Chemistry and Chemical Engineering, Nanjing University, Nanjing 210023, China; State Key Laboratory of Organic-Inorganic Composites, College of Materials Science and Engineering, Beijing University of Chemical Technology, Beijing 100029, China; State Key Laboratory of Organic-Inorganic Composites, College of Materials Science and Engineering, Beijing University of Chemical Technology, Beijing 100029, China; State Key Laboratory of Smart Sensing Materials, MOE Key Laboratory of Organic Integrated Circuits & Tianjin Key Laboratory of Molecular Optoelectronic Sciences, Department of Chemistry, School of Sciences, Tianjin University, Tianjin 300072, China

**Keywords:** stretchable electronics, organic field-effect transistors, polymer semiconductors, hydrogen bonds, healability

## Abstract

Stretchable polymer semiconductors are vital for intelligent technologies such as health monitoring and human–machine interactions, but suffer from a fundamental trade-off between charge transport and stretchability/self-healability. Herein, we introduce the concept of hierarchical hydrogen bonds to provide a multilevel dynamic interconnected polymer network that simultaneously delivers outstanding stretchability, notable self-healing ability and high charge carrier mobility. The conjugation breaker *N,N*-dicarbamoylpyridine-2,6-dicarboxamide is incorporated into the polymer backbone with different strengths of hydrogen bonds, affording a crack-onset strain up to 150% and 90% mobility recovery upon healing treatment. Crucially, the hierarchical hydrogen bonds enable close interchain stacking for efficient interchain charge transport while enhancing chain dynamics and mechanical compliance. Fully stretchable transistors based on our designed polymer show stable and high mobility up to 1.01 cm^2^ V^−1^ s^−1^ even under 150% strain, marking unprecedented performance for healable semiconductors. Hierarchical hydrogen-bonded engineering thus establishes a design paradigm for high-performance stretchable and healable polymer semiconductors.

## Teaser

This work highlights how hierarchical hydrogen bonds can be used to design stretchable, self-healing polymer semiconductors that achieve record performance even when stretched 150%.

## INTRODUCTION

Flexible transistors that provide signal-processing and computational functions have become increasingly important in wearable electronics, health monitoring, artificial skins and implantable bioelectronics [1–5]. Excellent electrical characteristics and mechanical stretchability can be realized by molecular design or blend engineering of polymer semiconductors [6–10]. Apart from that, self-healing ability is highly desirable in flexible transistors, which can improve the device performance and life by repairing inflicted damage [11,12]. As a key class of component materials, polymer semiconductors are ideal candidates to integrate the three functions with low-cost amenability to large-area printing and high-density device manufacturing [13–16]. The intrinsic self-healing capability depends on the chemical cross-linking formed by dynamic covalent or noncovalent bonds [17], wherein noncovalent interactions usually have a lower kinetic stability and a reversible process of dissociation and generation without huge energy consumption [18–21]. As one of the most important dynamic non-covalent bonds, hydrogen bonds have been incorporated into polymer backbones or sidechains to afford high stretchability and self-healing properties owing to their reversible and spontaneous formation [22–27]. The reversible nature of hydrogen bonds allows them to break for strain energy dissipation and reform to restore initial mechanical properties, enabling high strain tolerance in materials [25,26,28]. Generally, loosely packed structures with dynamic and mobile supramolecular chains are beneficial for stretchability and healability, while densely packed structures with strong interactions facilitate charge transport. This trade-off poses a formidable but worthwhile challenge for the development of stretchable and self-healing polymer semiconductors for flexible transistors [21,29–31].

There are only a few examples reported on the intrinsically stretchable and self-healable polymer semiconductors to date [25–27,32–34]. Bao *et al*. introduced a pyridine dicarboxamide (PDCA) moiety as a conjugation breaker into diketopyrrolopyrrole (DPP) based polymers to construct a N-H···O···H-N hydrogen-bonded network, which showed a hole mobility >0.10 cm^2^ V^−1^ s^−1^ at 100% strain in a fully stretchable transistor and recovered almost to initial mobility upon healing of the damaged films [25]. Oh *et al*. integrated peptide conjugation breakers into semiconducting polymers with a stable mobility of 0.12 cm^2^ V^−1^ s^−1^ under 100% strain, displaying self-healing properties with ∼55% recovery of carrier mobility via two N-H···O=C intermolecular hydrogen bonds in one breaker unit [33]. We previously designed a high-performance healable polymer semiconductor with 81% recovery of carrier mobility by *in situ* continuous hydrogen-bonded engineering, which exhibited mobility up to 1.08 cm^2^ V^−1^ s^−1^ under 100% strain [26]. Almost all stretchable and self-healable polymer semiconductors are constructed using single-stage hydrogen bonds from monofunctional groups, which are difficult to achieve excellent stretchability and healability while retaining good charge transport characteristics. Hierarchical hydrogen bonds that are formed by two or more functional units give rise to different strengths of hydrogen bonds, broadening the distribution of the binding strength of physical junctions, which in turn promotes dynamic cross-linking for intrinsic stretchability and self-healing ability via synergistic effects [35–38]. Unfortunately, hierarchical hydrogen bonds have not yet been investigated to develop stretchable and healable polymer semiconductors.

**Figure 1.**
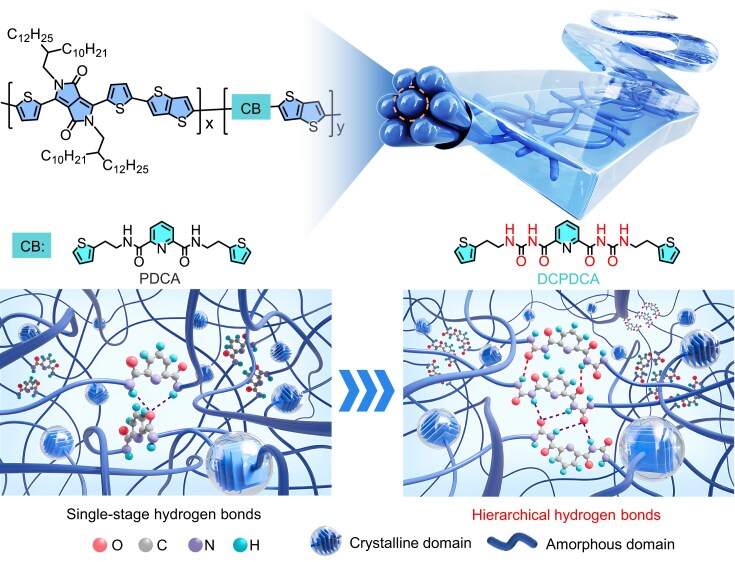
Design strategies of intrinsically stretchable and healable polymers via single-stage hydrogen bonds and hierarchical hydrogen bonds.

Herein, we incorporate *N,N*′-dicarbamoylpyridine-2,6-dicarboxamide (DCPDCA) into the conjugated DPP polymer backbone, constructing a multilevel dynamic interconnected network through hierarchical hydrogen bonding (Fig. 1). This design simultaneously yields outstanding stretchability, remarkable healability and high carrier mobility. As revealed by ^1^H NMR titration experiments, the DCPDCA breaker possesses two different strengths of intermolecular hydrogen bonds with extremely large self-association constants of 28.73 and 45.70, respectively, which are far higher than those of single hydrogen bonds. We found that terpolymer P3 with 10% ratio of DCPDCA displayed a crack-onset strain up to 150% and recovered to 90% of initial mobility upon healing treatment. Compared with single hydrogen bonds, hierarchical hydrogen bonds based on DCPDCA enable stronger interchain stacking for efficient interchain charge transport while enhancing chain dynamics and mechanical compliance for high stretchability and healability. Fully stretched transistors based on P3 polymer showed stable and high mobilities up to 1.01 cm^2^ V^−1^ s^−1^ even under 150% strain, which, to our knowledge, represents an unprecedented performance for intrinsically stretchable and healable semiconducting polymers [25–27,34]. Therefore, hierarchical hydrogen-bonded engineering can be used as a powerful strategy to construct stretchable and healable high-performance transistors.

## RESULTS AND DISCUSSION

### Design and characterization of hierarchical hydrogen-bonded polymer semiconductors

**Figure 2.**
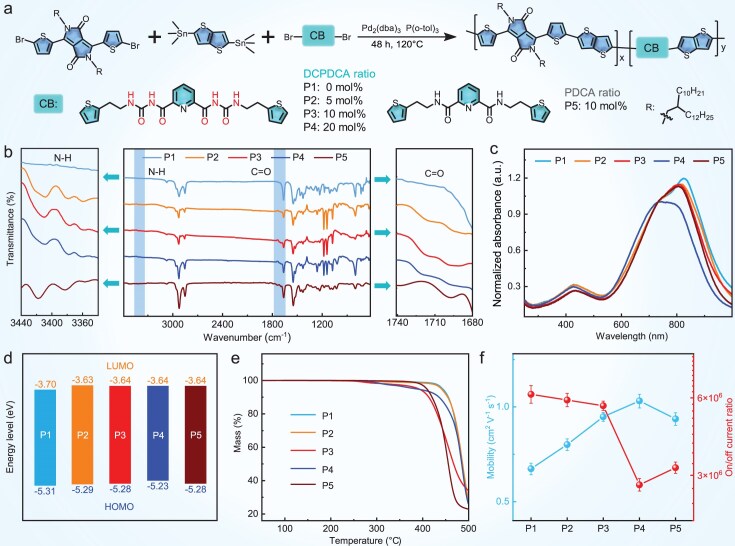
Synthesis and characterization of polymer semiconductors P1-P5. (a) Synthetic route of P1-P5 with the different feeding ratios of DCPDCA or PDCA. (b) FT-IR spectra of P1-P5. (c) Normalized UV-Vis/NIR absorption spectra of P1-P5 in the film state. (d) The energy-level diagram of P1-P5. (e) The thermogravimetric analysis of P1-P5. (f) The field-effect mobilities and on/off current ratios of P1-P5 measured by conventional OFETs.

We designed a class of acceptor-donor-conjugation breaker-donor type (A-D-CB-D type) random terpolymer semiconductors (P1-P5, Fig. 2a) to simultaneously achieve intrinsic stretchability, high mobility and self-healing properties. The polymers were synthesized by Stille polymerization of 3,6-bis(5-bromothiophen-2-yl)-2,5-bis(2-decyl-tetradecyl)pyrrolo [3,4-c]pyrrole-1,4(2H,5H)-dione (DPP), 2,5-bis(trimethylstannyl) thieno [3,2-b] thiophene (TT) and the brominated conjugation breakers *N,N*′-bis((2-(5-bromothiophen-2-yl)ethyl) carbamoyl) pyridine-2,6-dicarboxamide (DCPDCA-Br_2_) or *N*, *N*′-bis(2-(5-bromothiophen-2-yl)ethyl) pyridine-2,6-dicar boxamide (PDCA-Br_2_) units, with the detailed synthetic procedure being provided in the Supporting Information.

It is noteworthy that brominated conjugation breakers rather than trimethyl stannyl counterparts were used owing to easier accessibility with facile and efficient synthesis. The contents and properties of hydrogen bonds in the backbones can be finely regulated by changing the feeding molar ratios or species of the conjugation breakers. Three terpolymers (P2-P4) containing the DCPDCA moieties were prepared, in which the molar ratios of TT units versus DCPDCA units were 20:1, 10:1 and 5:1, respectively. For comparison, P1 without conjugation breaker units and terpolymer P5 with a 10:1 molar ratio of TT units versus PDCA units were also similarly prepared. The molecular weights of P1-P5 are in the range of 20.0–35.0 kDa (Table S1) measured by gel permeation chromatography (GPC). All polymers (P1-P5) showed good solubility in chlorobenzene after heating at 70°C, which allowed subsequent characterization and application in flexible devices.

In the Fourier transform infrared (FT-IR) spectra (Fig. 2b), P2-P5 exhibited a prominent peak in the range of 3400–3430 cm^−1^ originating from amide N-H stretching of DCPDCA or PDCA, whereas P1 did not show this peak. As determined by a curve-fitting analysis (Fig. S1), the intensity ratios of hydrogen-bonded N-H to free counterpart for polymers P2-P5 were further calculated to be 0.50, 0.59, 0.71 and 0.52, respectively. Moreover, we observed that P2-P4 showed two vibration peaks at ∼3380 and ∼3360 cm^−^^1^ in the hydrogen-bonded region, whereas P5 only presented a single absorption peak at 3383 cm^−^^1^, confirming the formation of a hierarchical hydrogen‑bonded network in P2-P4.

On the other hand, the bands in the range of 1650–1750 cm^−1^ for polymers P1-P5 were ascribed to the stretching vibration of the carbonyl (C=O) groups, which can be deconvoluted into three plausible sources: the DPP backbone, bound conjugation breakers, and free conjugation breakers [39–41]. H-bound peaks were observed at lower wavenumbers than free states due to weakened C=O bonds resulting from hydrogen bonding. The above analysis of the spectral characteristic peaks indicates that conjugation breakers (DCPDCA or PDCA) have been successfully synthesized in the polymer skeleton. To further investigate the influence of the conjugation breaker content on the optical and aggregation state of polymers, ultraviolet-visible/near-infrared (UV-Vis/NIR) absorption spectra of polymers P1-P5 were recorded in both thin film and solution states (Fig. 2c and Fig. S2). As the content of DCPDCA breaker in the polymer backbone increased, the absorption spectrum was blue-shifted due to the decreased degree of conjugation. In addition, the intensity ratios of 0–0 (*I*_0-0_) and 0–1 (*I*_0-1_) vibrational peaks became lower from P2 to P4, suggesting a reduced degree of aggregation with an increase in the DCPDCA ratio. Although both P3 and P5 exhibited similar absorption spectra in solution, P3 showed a bathochromic-shifted absorption spectrum with enhanced *I*_0-0_/*I*_0-1_ values in thin film, suggesting a stronger interchain stacking state that is beneficial to efficient charge carrier transport [16,42–44].

The electrochemical properties of polymer P1-P5 films (Fig. S3) were tested by cyclic voltammetry (CV), and the highest occupied molecular orbital (HOMO) and lowest unoccupied molecular orbital (LUMO) energy levels were estimated via the onset oxidation and reduction potentials (Fig. 2d). The polymers P1-P5 showed similar HOMO/LUMO energy levels as listed in Table S2. In addition, thermogravimetric analysis (TGA) results revealed that all polymers began to lose weight at temperatures above 370°C (Fig. 2e), which confirmed good thermal stability for potential applications under conventional conditions. The charge transport characteristics of polymer P1-P5 films were preliminarily evaluated by fabricating the organic field-effect transistors (OFETs) with a bottom gate/top contact (BGTC) configuration. P1-P3 showed satisfying initial hole mobilities (>1 cm^2^ V^−1^ s^−1^), while P4 and P5 exhibited decreased mobilities of 0.67 cm^2^ V^−1^ s^−1^ and 0.85 cm^2^ V^−1^ s^−1^, respectively (Fig. 2f, Figs S4–S8 and Table S3). Although the polymers showed reduced mobility with the increase in content of the conjugation breakers, the polymers with a small ratio of DCPDCA still retained excellent charge transport behaviors without significant disruption of the backbone conjugation. We further evaluated the electrical stability of the transistor devices in a 50% humidity chamber in nitrogen atmosphere. P1 and P3 exhibited a significant degradation in electrical performance under humid conditions after 7-d storage, while showing negligible decay in a dry nitrogen atmosphere when stored for 30 d, demonstrating a high susceptibility to moisture even for the parent polymer P1 without hydrogen-bonded breakers (Fig. S9) [45].

### Comparison of hydrogen bonding interactions between DCPDCA and PDCA

**Figure 3.**
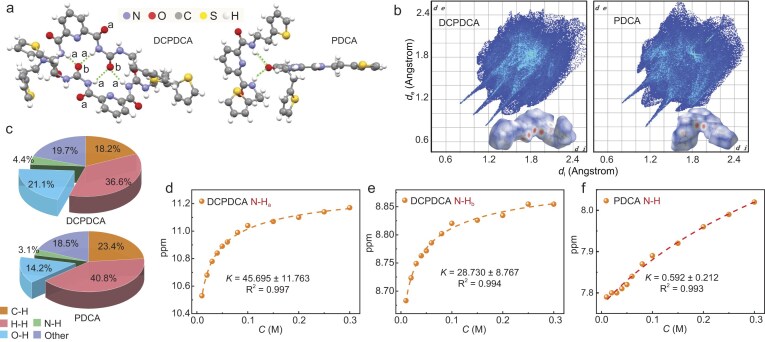
Hydrogen bonding interactions of conjugation breakers DCPDCA and PDCA. (a) Crystal structures of DCPDCA and PDCA. (b) Hirshfeld surface analyses for DCPDCA and PDCA. (c) Non-covalent interactions of DCPDCA and PDCA with the contribution percentage to crystal packing. (d–f) The equilibrium between monomeric and dimeric states of DCPDCA and PDCA as monitored N-H proton chemical shift during the ^1^H NMR titration experiment for N-H_a_ (d) and N-H_b_ (e) of DCPDCA and N-H (f) of PDCA. The association constant is extracted by fitting the data points into a binding equation.

To further investigate the hydrogen bonding interactions of DCPDCA, single crystals were obtained for X-ray crystallography analysis by slow evaporation of CH_2_Cl_2_ solution at room temperature (Fig. S10 and Tables S4 and S5). One pair was formed by hydrogen bonds in the single-crystal structure of DCPDCA, in which both molecules aligned nearly parallel across the long molecular axis (Fig. 3a). There were two sets of intermolecular hydrogen bonds (N-H_a_···O_b_···H_a_-N: 2.067, 2.068 Å; 2.121, 2.216 Å) among one C=O_b_ group of one molecule and two N-H_a_ groups of another molecule (Fig. S11). The pyridyl ring was almost coplanar with its adjacent trans amide groups, exhibiting close π-π interactions between neighboring molecules [46]. In contrast, only one set of intermolecular hydrogen bonds (N-H···O···H-N: 2.235, 2.095 Å) accounted for pair formation in the single-crystal structure of the reference PDCA (Fig. S11), in which the long molecular axis of one molecule was almost perpendicular to that of the other molecule. Moreover, the interaction energies of hydrogen bonds for DCPDCA and PDCA were calculated in the optimized geometry of dimers based on density functional theory (m08/6–31g**). The two sets of hydrogen bonds of DCPDCA showed higher interaction energies of −6.51/−9.42 and −6.94/−2.13 kcal/mol, respectively, while those of PDCA had lower interaction energies of −5.11/−2.18 kcal/mol.

Hirshfeld surface analysis and its corresponding fingerprint plot calculated by CrystalExplorer were used to analyze the contributions of various intermolecular interactions in a crystal lattice [47]. As shown in Fig. 3b, the red-white-blue schemes can distinguish whether contacts are shorter than the van der Waals distance or not in the Hirshfeld surface plot. The denser surface mapped in red spots of the DCPDCA unit was mainly distributed in N and O atoms, representing closer intermolecular distances. It is clear that DCPDCA exhibited more pronounced O-H non-covalent interactions with the contribution percentage of 21.1% while PDCA showed less pronounced O-H non-covalent interactions with the contribution percentage of 14.2% to crystal packing (Fig. 3c, and Figs S12 and S13). To quantitatively determine the hydrogen bonding strength for DCPDCA and PDCA, we performed a ^1^H NMR titration experiment to measure the self-association constants [33,40,48]. Briefly, a series of solutions with different concentrations were prepared by dissolving DCPDCA or PDCA conjugation breakers in CDCl_3_, and ^1^H NMR spectra were collected to enable a reliable fit to the equation [48]:


\begin{eqnarray*}
\delta = {\delta }_c + \frac{{{\delta }_c - {\delta }_a}}{{4K{C}_0}}\left( {1 - \sqrt {1 + 8K{C}_0} } \right).\end{eqnarray*}

The ^1^H NMR titration experiment and fitting data of two conjugation breakers are shown in Fig. 3d–f and Figs S14 and S15. It can be seen that chemical shifts (*δ*) of the N-H protons increased with concentration; *δ*_c_ and *δ*_a_ represent the chemical shift of the N-H proton in H-bound state and free state, respectively. *K* represents the self-association constant in equilibrium state, while *C*_0_ is the formal solution concentration. Although we cannot observe the strong hydrogen bonds from N-H_b_ in a single crystal structure, the intermolecular interactions are definitely in the aggregated state to make different chemical shifts. *K* values were extracted as summarized in Table S6, which increased significantly from 0.59 for N-H to 28.73 for N-H_b_ or 45.70 for N-H_a_ by changing the conjugation breaker from PDCA to DCPDCA, suggesting the formation of hierarchical hydrogen bonds. The self-association constant *K* for DCPDCA is the highest value of hydrogen-bonded conjugation breaker for stretchable polymer semiconductors [33,40,48].

### Improved mechanical properties and film microstructures via hierarchical hydrogen bonds

**Figure 4.**
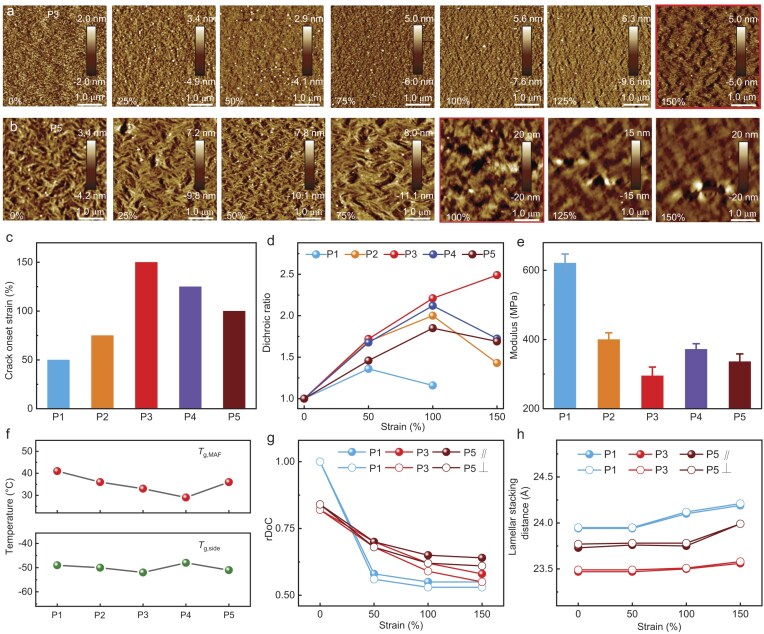
Mechanical properties and film microstructures of polymer semiconductors. (a–b) AFM height images for polymer P3 (a) and P5 (b) under different strains. (c) Crack onset strains for polymer P1-P5 from optical microscopy. (d) Dichroic ratio values of P1-P5 films at different strains. (e) Elastic moduli for polymer P1-P5 measured by AFM nanomechanical mapping. (f) Glass transition temperatures of polymer backbone and side chain for polymer P1-P5. (g and h) The changes of relative degree of crystallinity (g) and lamellar stacking distances (h) of P1, P3 and P5 films under different strains.

The morphology analysis of the thin films under different strains was characterized by optical microscopy and atomic force microscopy (AFM) (Fig. 4a and b and Figs S16–S23). Some tiny cracks emerged in P1 thin film at 50% strain, while cracks for P2, P4 and P5 appeared at strains of 75%, 125% and 100%, respectively. In comparison, there were only a few sparse microcracks detected within the P3 thin film at strains as high as 150% as measured by optical microscopy. Although the nanocrack formation was observed for P3 at 150% strain from AFM measurements, the connectivity of the nanofiber network still remained to ensure efficient charge transport. Notably, P3 showed a much higher crack onset strain (150%) than that of P5 (100%) with the same ratio of conjugation breakers (10 mol%), suggesting that the DCPDCA unit with hierarchical hydrogen bonds is more conducive to improving stretchability than the PDCA unit (Fig. 4c).

Consequently, we used polarized UV-Vis/NIR spectroscopy to measure the degree of polymer chain alignment under strain, which was quantified by dichroic ratios (Fig. 4d, and Figs S24 and S25). The dichroic ratios initially increased because of the strain-induced chain alignment and then decreased due to the formation of cracks when further stretched [31]. P3 thin film showed the largest dichroic ratio among all the polymers, indicating the highest degree of polymer chain alignment. The dichroic ratio of P3 still increased at 150% strain while that of P5 decreased sharply at the same strain.

Next, the tensile moduli of five polymer films were measured by AFM nanomechanical mapping (Fig. 4e and Fig. S26). Compared with the parent polymer P1, P2-P5 showed reduced moduli by introducing conjugation breakers. However, as manifested in the case of P4, the higher ratio of the DCPDCA unit gave rise to an increased modulus due to stronger hydrogen bonding interactions [49]. P3 with 10% DCPDCA moieties had the lowest modulus value (295.3 MPa), which was <P5 by 12.2%, thus accounting for the excellent film deformability and ductility. We further performed tensile testing of the polymer films by employing the film-on-water (FOW) technique (Table S7). The typical stress-strain curves are shown in Fig. S27. The variation trend in derived tensile moduli for P1-P5 was consistent with that measured by AFM nanomechanical mapping. The parent polymer P1 showed a toughness of 0.69 MJ m^−3^ and maximum stretchability of 6.4%. Those values of P2, P4 and P5 were at the same level relative to P1. Impressively, P3 with appropriate content of hierarchical hydrogen bonds possessed the largest degree of toughness (6.04 MJ m^−3^) and maximum stretchability (50.6%), one order of magnitude higher than that of P1. One hundred cyclic stretching tests revealed that the modulus of P1 decreased significantly as the number of cycles increased (Fig. S28). For P2-P5, the modulus decreased significantly during initial stretching but stabilized as the number of cycles increased while the residual strain remained almost unchanged. P2, P3 and P5 showed relatively larger residual strain probably due to locked deformation by hydrogen bonding interactions. In addition, dynamic mechanical analysis (DMA) on P3 and P5 was performed to compare their energy dissipation characteristics (Fig. S29), which was achieved by reinforcing the polymer films with woven glass fiber [50]. The storage modulus of P3 was much higher than that of P5 below 30 °C, which was the result of increased stiffness in the hierarchical hydrogen-bonded system. The tan *δ* value of P3 was also larger than that of P5, suggesting that more energy was dissipated by hierarchical hydrogen bonds. Notably, the glass transition of P3 was very broad with multilevel peaks in the range of −12 to −53 °C, which confirmed that the relaxation behavior of polymer chains was produced by the dynamic dissociation of hierarchical hydrogen bonds [51]. We further investigated the influence of conjugation breakers on polymer chain dynamics by alternating current (ac) chip calorimetry [52]. The glass transition temperatures (*T*_g_) of the polymer backbone decreased with increasing content of DCPDCA (Fig. 4f, and Figs S30 and S31). P3 had relatively lower *T*_g_ of polymer backbone than P5, suggesting a higher degree of chain dynamics. Although P3 had a higher *T*_g_ value than P4, it possessed the largest mass percentage ratio (61.8%, Fig. S31) according to the step change of heat capacity at the corresponding glass transitions when compared with those of the side chains, resulting in the lowest modulus value.

To understand the impact of the conjugation breaker on microstructures and crystallinity, the stretched films were characterized via grazing incidence wide angle X-ray scattering (GIWAXS), as depicted in Fig. 4g and h, Figs S32–S36 and Table S8. The π-π stacking distances of polymers P1, P3 and P5 were calculated to be 3.68 Å, 3.72 Å and 3.75 Å (Fig. S37), respectively. A smaller π-π distance generally indicates stronger molecular orbital overlap, which favors efficient charge carrier transport. This is consistent with the trend in their initial field‑effect mobilities. The broad diffused peak (100) and weak peak (200) in the out-of-plane (q_z_) direction showed the low crystallinity of all polymers P1-P5. Next, we analyzed the relative degree of crystallinity (rDoC) of stretched films based on the (100) lamellar stacking peak in the 2D diffractograms. As indicated in Fig. 4g, the initial rDoC decreased with increasing content of the conjugation breakers, which corresponded well with the glass transition temperatures of the polymer backbone. We observed that the rDoC of P1 decreased with strains from 0% to 50% and then plateaued, suggesting partial energy dissipation through breakage of the crystalline regions and further crack formation at higher strain [26]. The rDoC of P3 decreased steadily with increasing strain from 0% to 150% while that of P5 became plateaued at 100% strain, further demonstrating that the hierarchical hydrogen-bonded network formed by DCPDCA units can be stepwise broken to facilitate strain energy dissipation. Furthermore, the corresponding lamellar stacking distance extracted from (100) peaks can also explain the change of crystalline domains (Fig. 4h). The lamellar distances of P3 and P5 were slightly smaller than that of P1 mainly due to the replacement of the branched alkyl chains with fewer steric conjugation breakers in the backbone. P3 exhibited the least amount of change under strain, which suggested that the crystalline domains underwent smaller deformation. This is mainly because strain energy was dissipated by the gradual disruption of hierarchical hydrogen bonds of the DCPDCA unit.

### Stable electrical properties under mechanical deformation and excellent self-healing properties

To explore the charge transport behaviors under different mechanical deformation, the stretched films were transferred onto SiO_2_/Si substrates to prepare transistor devices for electrical measurements (Fig. S38). We found that the average mobilities of P3 in the parallel direction (//) remained almost unchanged with strains up to 150% (Figs S39 and S40), which showed excellent stretchability and charge transfer properties. The average mobilities of P3 in the perpendicular direction did not decrease significantly at strains <100%, retaining the stability of output currents. Further investigation revealed that the cracks of polymer P3 became increasingly dense as the strain increased from 200% to 300%, indicating that the film suffered severe damage (Fig. S41). Electrical performance measurements showed that the mobility of the P3 film decreased to 0.13 cm^2^ V^−1^ s^−1^ under 300% strain in the parallel direction, while the mobility dropped to 0.04 cm^2^ V^−1^ s^−1^ in the perpendicular direction (Fig. S42). The significant deterioration of electrical performance for P3 in both directions was attributed to the formation of cracks within the film, which disrupt efficient charge transport. In contrast, the average mobility of P1 and P5 in both directions decreased obviously upon increasing the strain to 50% or 100%, respectively. The trends in mobilities for P1, P3 and P5 under strain agreed well with the relative changes in crack onset strain and dichroic ratio. Next, standard stretching cycle tests were conducted on three polymers (P1, P3 and P5) at 50% strain for 100 cycles. Along parallel or perpendicular directions of applied stress (Figs S43 and S44), the mobilities of P3 decreased by only 2%–5% compared to the initial properties while those of P1 and P5 dropped by 80%–90% and 7%–12%, respectively, demonstrating that the introduction of DCPDCA units in P3 endowed the polymer with superior durability and robustness compared to those containing PDCA units or lacking hydrogen-bonded groups.

**Figure 5.**
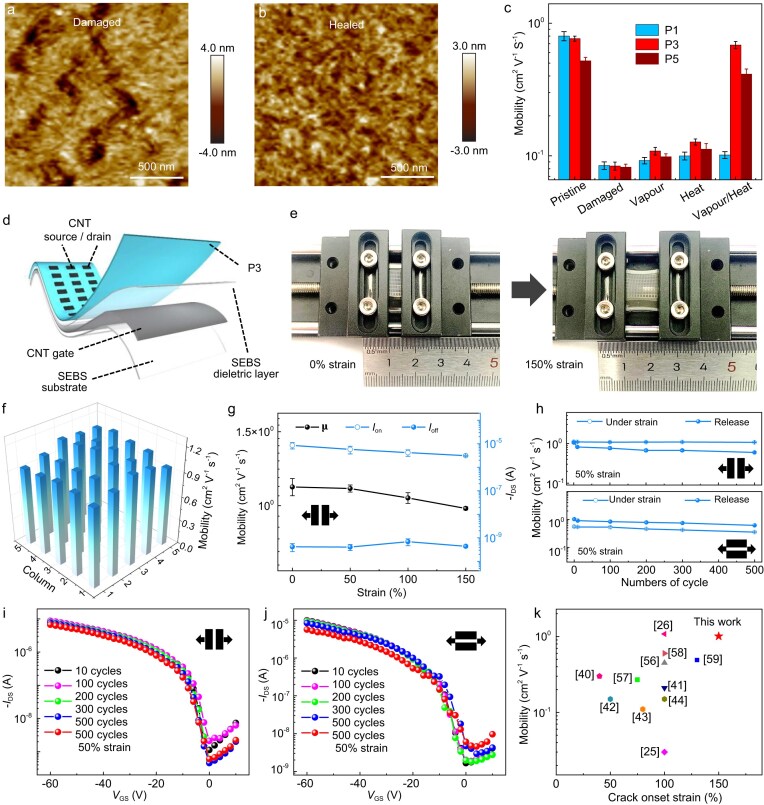
Self-healing properties of polymers and fully stretchable transistors based on P3 films possessing a hierarchical hydrogen-bonded network. (a) AFM image for the damaged film of P3. (b) AFM image for the healed film of P3. (c) Field-effect mobilities of damaged, post-processing process, healed transistor devices for P1, P3 and P5. (d) Device structure of a fully stretchable transistor with a bottom-gate top-contact configuration. (e) Images of the fully stretchable device under 0% and 150% strain. (f) Distributions of field-effect mobilities for the fully stretchable transistor devices based on P3. (g) Field-effect mobilities, on-currents and off-currents as a function of various strains parallel to the charge transport direction. (h) Mobilities as a function of stretching-releasing cycles under 50% strain parallel and perpendicular to the charge transport direction. (i and j) Transfer curves of P3 after 500 stretching-releasing cycles under 50% strain parallel (i) and perpendicular (j) to the charge transport direction. (k) Comparison in mobilities and crack onset strain values between P3 and representative work in the literature.

In addition to the fascinating intrinsic stretchability, the hydrogen bonds of conjugation breakers in polymer backbone also provided self-healing capabilities due to the dynamic reversibility of hydrogen bonds after breakage. To investigate the self-healing capability of the polymers (Fig. 5a–c and Figs S45–S51), the films were intentionally damaged to form nanocracks with strain of 50% for P1, 150% for P3 and 100% for P5 up to 10 cycles. The solvent vapor time and annealing temperature were optimized for healing treatment, which gave rise to significant morphological improvement for the cracked polymer P3 films (Figs S45–S47). This was further quantitatively evaluated through comparative analysis of AFM height images and the recovery of electrical conductivity. As revealed by the AFM height images of damaged and treated P1, P3 and P5, the cracks of P1 film were almost unchanged before and after treatment (Fig. S48), but those of P3 and P5 diminished completely with a much smoother morphology for P3 after post-treatment (Fig. 5a and b, and Figs S49 and S50). P3 showed the most superior recovery of charge transport after treatment with mobility being 90% of that of the pristine thin film while the mobility of P5 was recovered to 78% of that of the pristine film (Fig. 5c and Fig. S51). Overall, these results indicated that polymer P3 with hierarchical hydrogen bonds exhibited much more efficient healing capability when suffering mechanical deformation and nanocrack formation, hence holding great prospects for practical applications.

We hypothesized that the hierarchical hydrogen bonds formed by DCPDCA units were important to achieve excellent carrier mobility, stretchability and healability simultaneously. First, the DCPDCA units tended to align in a nearly parallel direction as revealed by single crystal X-ray diffraction analysis, which induced adjacent polymer chains of P3 to take a nearly parallel alignment. This further accounted for stronger interchain stacking indicated by the larger *I*_0-0_/*I*_0-1_ value relative to P5, thereby facilitating efficient interchain charge transport. Second, the higher chain dynamics and lower tensile modulus of P3 compared with P5 substantially contributed to the improvement in stretchability. Third, the hierarchical hydrogen bonds of DCPDCA with various bonding strengths were favorable to the multilevel dynamic cross-linking for intrinsic stretchability and self-healing ability [38,53]. P3 possessed various hydrogen bonds to stepwise dissipate the strain energy, showing notably enhanced stretchability. When damaged, P3 thin films were supposed to be healed at a higher efficiency considering that the different strengths of hydrogen bonds had a high probability of being reformed, which is consistent with the self-healing ability measurements [54].

### Fully stretchable transistors

Finally, based on the analysis and comparison of the polymers, fully stretchable transistors were fabricated using P3 as the active semiconductor layer (Fig. 5d and e). The transistors were fabricated in a BGTC configuration with polystyrene-block-poly(ethylene-ranbutylene)-block-polystyrene (SEBS) 1062 as the substrate, carbon nanotubes (CNTs) as gate and source/drain electrodes, SEBS (1052) as the dielectric layer [55]. Representative transfer curve and output curves of stretchable transistors suggested stable and ideal p-channel transistor characteristics (Fig. S52). The devices showed an average mobility of 1.05 cm^2^ V^−1^ s^−1^ and a maximum mobility of 1.17 cm^2^ V^−1^ s^−1^ at 0% strain (Fig. 5f). When the device was stretched along the charge transport direction, the field-effect mobility remained almost unchanged at 100% strain (1.04 cm^2^ V^−1^ s^−1^) and even at 150% strain (1.01 cm^2^ V^−1^ s^−1^) (Fig. 5g and Fig. S53), which is an unprecedented value at such high strain for intrinsically stretchable and healable semiconducting polymers [25–27]. In addition, the on-current remained as stable as 66% of the initial value though the carrier mobility along the perpendicular direction showed a distinct drop at 150% strain (Fig. S54). We further evaluated the stretching durability for P3 film at 50% strain and observed that the mobilities of the fully stretchable transistors maintained stable even after 500 stretching cycles in both parallel and perpendicular directions (Fig. 5h–j and Fig. S55). To the best of our knowledge, it showed the best comprehensive performance in terms of the stretchability, mobility and self-healing capability compared with other intrinsically stretchable polymers, without the aid of elastomers, reported so far (Fig. 5k) [25,26,40–44,56–59]. In addition, the array maintained satisfactory electrical stability under various mechanical deformations, including poking, twisting and biaxial stretching, thereby laying a solid material foundation for potential applications (Fig. S56).

## CONCLUSION

In conclusion, we have incorporated hierarchical hydrogen-bonded conjugation breakers into semiconducting polymer backbones, facilitating multilevel dynamic cross-linking for intrinsic stretchability and self-healing ability while simultaneously maintaining high carrier mobilities. The polymer P3 with 10 mol% DCPDCA breakers exhibited a large crack onset strain up to 150%, small tensile modulus as well as high polymer chain dynamics. The morphology and electrical characteristics of the damaged P3 films can be almost recovered after solvent and thermal annealing treatment. Hierarchical hydrogen bonds based on DCPDCA induced strong interchain stacking for efficient interchain charge transport while improving stretchability and healability by broadening the distribution of the binding strength of dynamic cross-linking. Fully stretchable transistors based on P3 showed an excellent mobility up to 1.01 cm^2^ V^−1^ s^−1^ under 150% strain, which is the highest value for intrinsically stretchable and healable polymer semiconductors. Furthermore, the stretchable transistors did not suffer from performance degradation along both parallel and perpendicular directions even after being subjected to a repeated strain of 500 cycles. The incorporation of hierarchical hydrogen bonds into the conjugated backbone provides a promising strategy to solve the long-standing issue of charge transport, stretchability and self-healing ability.

## METHODS

### Synthesis of various conjugated polymers

All of the studied conjugated polymers based on DPP main-chain structures were synthesized by Stille coupling polycondensation with dibrominated DPP monomers, stannylated compounds (TT), dibrominated DCPDCA or PDCA monomers by control of feeding ratios of the relative monomers. Details of all synthetic procedures are available in the Supplementary Information.

### Thin-film preparation

Wafers with 300 nm silicon dioxide on n^++^ Si were cleaned with deionized water, acetone and isopropanol, in that order, and then modified with an OTS self-assembled monolayer according to previous reported methods. Polymers were dissolved in chlorobenzene (5 mg/ml) at 80°C and the polymer solutions were spin-coated on OTS-treated SiO_2_ wafers at 1500 rpm for 60 s. The thickness of the polymer films was controlled at ∼30 nm. The obtained semiconducting films were annealed at 200°C in nitrogen for 30 min.

### Materials characterization

PDMS films were prepared at ratios of 15:1 (base: crosslinker, w/w) and cured overnight at 80°C and then cut into 1 × 2 cm slabs used for the laminating substrate to transfer the polymer thin films. The polymer thin films peeled from OTS-treated SiO_2_ wafers with a PDMS slab were transferred onto SiO_2_/Si substrates. Then gold source and drain electrodes (40 nm in thickness) were deposited with shadow masks (W/L = 8.2). All samples have bottom-gate top-contact device architecture. Electrical characterization of the OFETs was conducted with Keithley 4200 in a N_2_ glove box. The capacitance of SiO_2_ gate dielectric capacitance was 10 nF cm^−2^.

### Self-healing process

The films were damaged to form nanocracks with strains of 50% for P1, 150% for P3 and 100% for P5 up to 10 cycles. Then the sample was initially treated with CHCl_3_ vapor annealing for 10 min and then thermal annealing in a N_2_ glove box at 120°C for 10 min. This process was repeated three times.

### Fully stretchable transistors

SEBS H1221 (from Asahi Kasei) was dissolved in toluene (250 mg mL^−1^) at 80°C and then poured onto a 2 × 2 cm Si/SiO_2_ substrate, then the obtained films were peeled and used as substrates for fully stretchable transistors. CNT solution diluted to 0.2 mg ml^−1^ with deionized water was cast onto the Si/SiO_2_ wafers patterned with capton tape. The CNT gate electrodes were subsequently transferred onto SEBS substrates. SEBS H1052 (from Asahi Kasei) was dissolved in toluene (70 mg mL^−1^) and filtered with a 0.22 um filter. The solution was spin coated onto the OTS-Si/SiO_2_ substrates at 1000 rpm for 60 s. The resulting film was annealed in a vacuum drying oven at 100°C for 1 h. This SEBS dielectric layer was then transferred onto the CNT gate electrode. Next, the semiconducting polymer film was transferred onto the dielectric layer. Finally, the diluted CNT solution was then spray-coated onto the semiconducting layer through a shadow mask with channel length (L) of 1000 µm and width (W) of 200 µm. The mobilities were calculated with measured device geometry and dielectric capacitance under strain (Table S9).

## Supplementary Material

nwag162_Supplemental_File
