# Supplementary material for: Simultaneous enhancement of stretchability, healability and carrier mobility in polymer semiconductors via hierarchical hydrogen-bonded engineering
Source: Natl Sci Rev. 2026 Mar 14;13(9):nwag162. doi: 10.1093/nsr/nwag162 (PMC13228146; doi:10.1093/nsr/nwag162)

Supplementary Information

Simultaneous Enhancement of Stretchability, Healability and Carrier Mobility in Polymer Semiconductors via Hierarchical Hydrogen-Bonded Engineering

Haoguo Yue[1,2,†], Ying Wang[1,3,†], Zhihao Meng[1,†], Shaochuan Luo[4], Jiasen Quan[1], Ruiwei Zhou[1], Haonan Geng[1], Xinyu Xia[1], Junxuan Tu[1,3], Jun Jin[1,5], Dongshan Zhou[4], Lei Zhang[1], Yonggang Zhen[1,*], Wenping Hu[3]

[1] State Key Laboratory of Organic-Inorganic Composites, College of Materials Science and Engineering, Beijing University of Chemical Technology, Beijing, 100029, China

[2] Shandong Key Laboratory of Chemical Energy Storage and New Battery Technology, School of Materials Science and Engineering, Liaocheng University, Liaocheng, 252000, China

[3] State Key Laboratory of Smart Sensing Materials, MOE Key Laboratory of Organic Integrated Circuits & Tianjin Key Laboratory of Molecular Optoelectronic Sciences, Department of Chemistry, School of Sciences, Tianjin University, Tianjin, 300072, China

[4] School of Chemistry and Chemical Engineering, Nanjing University, Nanjing, 210023, China

[5] State Key Laboratory of Fine Chemicals, School of Chemical Engineering, Dalian University of Technology, Dalian, 116024, China

† These authors contributed equally to this work.

1. Materials synthesis and characterization

Synthetic route:

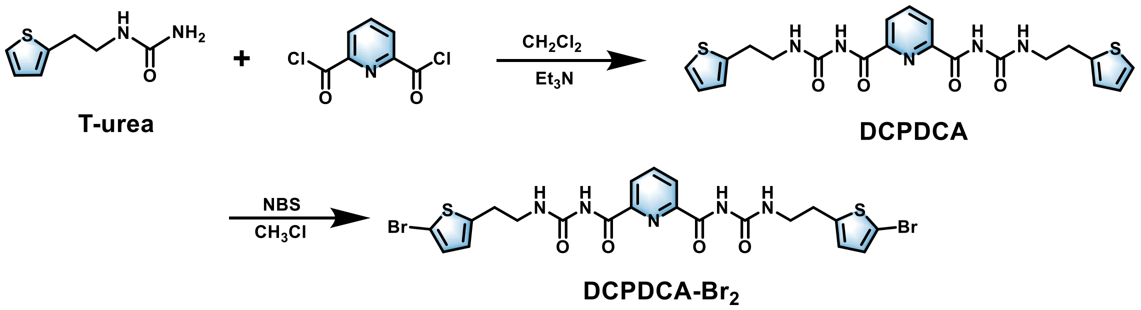

Synthetic route of monomer DCPDCA-Br$_2$.

Compound DCPDCA: The compound 1-(2-(Thiophen-2-yl)ethyl)urea (T-urea) (340.0 mg, 2 mmol) and 75 ml anhydrous tetrahydrofuran (THF) were added to a 250 mL fused Schlenk tube under nitrogen atmosphere. Next, pyridine (240 μl, 3 mmol) was dropped to the 250 mL fused Schlenk tube. Then, the compound pyridine-2,6-dicarbonyl dichloride (204.0 mg, 1 mmol) and 5 ml anhydrous tetrahydrofuran were added to another 10 mL fused Schlenk tube under nitrogen atmosphere, and the prepared solution was slowly added to the 250 mL reaction tube containing T-urea. The reaction mixture was stirred for 24 h at room temperature under nitrogen atmosphere. Next, the crude product was obtained by removal of tetrahydrofuran in the tube under reduced pressure, which was further recrystallized in ethanol to give the pure product DCPDCA (yield: 401.1 mg, 85%). ^{1}H NMR (400 MHz, Chloroform-d) δ 10.98 (s, 2H), 8.78 (t, J = 5.8 Hz, 2H), 8.42 (d, J = 7.8 Hz, 2H), 8.11 (t, J = 7.8 Hz, 1H), 7.12 (dd, J = 5.1, 1.2 Hz, 2H), 6.92 (dd, J = 5.1, 3.4 Hz, 2H), 6.83 (dd, J = 3.4, 1.1 Hz, 2H), 3.61 (td, J = 7.0, 5.7 Hz, 4H), 3.08 (t, J = 7.0 Hz, 4H). ^{13}C NMR (101 MHz, Chloroform-d) δ 164.55, 153.92, 147.92, 141.21, 139.13, 126.96, 126.75, 125.25, 123.83, 41.42, 29.97. HRMS calcd for $C_{21}H_{22}N_5O_4S_2^+$ $[M+H]^+$, 472.1108; found, 472.1095.

Compound DCPDCA-Br$_2$: The compound DCPDCA (94.3 mg, 0.2 mmol) and 10 mL chloroform ($CHCl_3$) were added to a 50 mL fused Schlenk tube under nitrogen atmosphere. Then, *N*-Bromosuccinimide (178.0 mg, 1 mmol) was dissolved in 3 mL acetonitrile and slowly added to the reaction tube at 0°C. The reaction mixture was stirred 12 h at room temperature in the dark. Next, the crude product was obtained by removal of organic solvents in the tube under reduced pressure, which was further recrystallized in ethanol to give the pure product DCPDCA-Br$_2$ (yield: 103.2 mg, 82%). ^{1}H NMR (400 MHz, Chloroform-d) δ 10.76 (m, 2H), 10.81–10.68 (m, 2H), 8.68 (d, J = 7.2 Hz, 2H), 8.44 (d, J = 7.8 Hz, 2H), 8.16 (t, J = 7.8 Hz, 1H), 6.87 (d, J = 3.7 Hz, 2H), 6.63

(dd, J = 3.6, 1.0 Hz, 2H), 3.60 (q, J = 6.6 Hz, 4H), 3.04 (t, J = 6.8 Hz, 4H). ^{13}C NMR (101 MHz, Chloroform-d) δ 164.55, 153.96, 147.91, 143.06, 139.41, 129.86, 127.02, 125.90, 109.92, 41.17, 30.49. HRMS calcd for $C_{21}H_{20}Br_2N_5O_4S_2^+$ $[M+H]^+$, 627.9318; found, 627.9309.

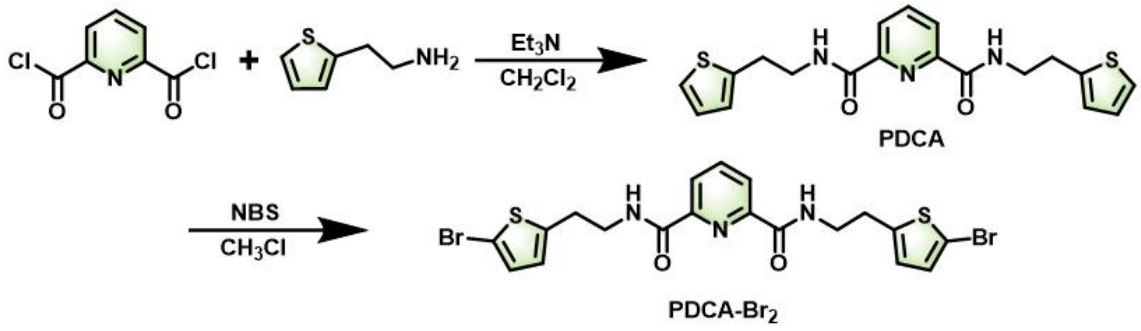

Synthetic route of monomer PDCA-Br$_2$.

Compound PDCA: The compound *N,N'*-bis(2-(thiophen-2-yl)ethyl)pyridine-2,6-dicarboxamide (PDCA) was prepared according to known procedure [1].

Compound PDCA-Br$_2$: The compound PDCA (77.1 mg, 0.2 mmol) and 10 mL chloroform ($CHCl_3$) were added to a 50 mL fused Schlenk tube under nitrogen atmosphere. Then, *N*-Bromosuccinimide (178.0 mg, 1 mmol) was dissolved in 3 mL acetonitrile and slowly added to the reaction tube at 0°C. The reaction mixture was stirred 12 h at room temperature in the dark. Next, the crude product was obtained by removal of organic solvents in the tube under reduced pressure, which was further recrystallized in ethanol to give the pure product PDCA-Br$_2$ (yield: 85.8 mg, 79%). ^1H NMR (400 MHz, Chloroform-d) δ 8.35 (d, J = 7.7 Hz, 2H), 8.05 (t, J = 7.8 Hz, 1H), 7.85–7.75 (m, 2H), 6.88 (d, J = 3.7 Hz, 2H), 6.64 (d, J = 3.7 Hz, 2H), 3.75 (q, J = 6.6 Hz, 4H), 3.10 (t, J = 6.6 Hz, 4H). ^{13}C NMR (101 MHz, Chloroform-d) δ 163.53, 148.73, 143.05, 139.34, 130.02, 126.35, 125.24, 110.18, 40.69, 30.66. HRMS calcd for $C_{19}H_{18}Br_2N_3O_2S_2^+$ $[M+H]^+$, 541.9202; found, 541.9195.

Polymer P1: In the Schlenk tube, 2,5-bis(trimethylstannyl)thieno[3,2-b]thiophene (TT, 93.2 mg, 0.2 mmol), 3,6-bis- (5-bromo-thiophen-2-yl)-2,5-bis(2-decyltetradecyl)-1,4-dioxo-pyrrolo[3,4-c]pyrrole (DPP, 226.2 mg, 0.20 mmol), $Pd_2(dba)_3$ (6 mg), $P(o\text{-}tol)_3$ (16.4 mg), and dry chlorobenzene (3.5 mL) were added to a 10 mL fused Schlenk tube. The reaction system was handled with the standard freeze-vacuum-thaw operation. After raised to the room temperature, the reaction tubes were heated to 120°C and stirred for 48 h. Next the reaction mixture was quenched with 3 mL hydrochloric acid dissolved in 100 mL anhydrous methanol and stirred for 2

h. The polymer solids were collected and then extracted with methanol, acetone and hexane for 12 h each by Soxhlet extractor. Finally, polymer **P1** was dissolved in chloroform and then precipitated again in methanol. After collected, **P1** was dried under vacuum at 60°C for 24 h and used for subsequent characterization and testing. Yield (178 mg, 79%). GPC: Mn = 25.7 kDa, Mw = 53.1 kDa, PDI = 2.07.

Polymer P2: In the Schlenk tube, 2,5-bis(trimethylstannyl)thieno[3,2-b]thiophene (TT, 93.2 mg, 0.2 mmol), 3,6-bis-(5-bromo-thiophen-2-yl)-2,5-bis(2-decyltetradecyl)-1,4-dioxo-pyrrolo[3,4-c]pyrrole (DPP, 214.9 mg, 0.19 mmol), *N,N'*-bis((2-(5-bromothiophen-2-yl)ethyl)carbamoyl)pyridine-2,6-dicarboxamide (DCPDCA-Br_2, 6.3 mg, 0.01 mmol), $Pd_2(dba)_3$ (6 mg), $P(o\text{-}tol)_3$ (16.4 mg), and dry chlorobenzene (3.5 mL) were added to a 10 mL fused Schlenk tube. The reaction system was handled with the standard freeze-vacuum-thaw operation. After raised to the room temperature, the reaction tubes were heated to 120°C and stirred for 48 h. Next the reaction mixture was quenched with 3 mL hydrochloric acid dissolved in 100 mL anhydrous methanol and stirred for 2 h. The polymer solids were collected and then extracted with methanol, acetone and hexane for 12 h each by Soxhlet extractor. Finally, polymer **P2** was dissolved in chloroform and then precipitated again in methanol. After collected, **P2** was dried under vacuum at 60°C for 24 h and used for subsequent characterization and testing. Yield (172 mg, 75%). GPC: Mn = 22.5 kDa, Mw = 40.4 kDa, PDI = 1.79.

Polymer P3-P4: The whole experimental procedure can be referred to that of polymer **P2**, except for the feed ratios of DPP and DCPDCA-Br_2 are different. For **P3**, the feed ratios of DPP (203.6 mg, 0.18 mmol), DCPDCA-Br_2 (12.6 mg, 0.02 mmol) have adjusted based on the polymer design. Yield: (180 mg, 77%). GPC: Mn = 22.8 kDa, Mw = 48.7 kDa, PDI = 2.13. For **P4**, the feed ratios of DPP (180.9 mg, 0.16 mmol), DCPDCA-Br_2 (25.2 mg, 0.04 mmol) have also changed based on the polymer design. Yield: (169 mg, 73%). GPC: Mn = 32.9 kDa, Mw = 58.2 kDa, PDI = 1.77.

Polymer P5: The whole experimental procedure can be referred to that of polymer **P2**, except for DCPDCA-Br_2 was replaced by PDCA-Br_2. For **P5**, the feed ratio of PDCA-Br_2 (10.9 mg, 0.02 mmol) is same as the **P3**. Yield: (167 mg, 73%). GPC: Mn = 28.7 kDa, Mw = 66.7 kDa, PDI = 2.32.

Table S1. Molecular weight characterization of polymers P1–P5 [a]

Polymer	M_n[b] [kDa]	M_w[kDa]	PDI
**P1**	25.7	53.1	2.07
**P2**	22.5	40.4	1.79
**P3**	22.8	48.7	2.13
**P4**	32.9	58.2	1.77
**P5**	28.7	66.7	2.32

[a)] Abbreviation: M_n, number-average molecular weight. M_w, weight-average molecular weight. PDI, polymer dispersity index. [b)] Determined by high-temperature gel permeation chromatography (GPC) using 1,2,4-trichlorobenzene as the eluent at 160°C and calibrated using polystyrene standards.

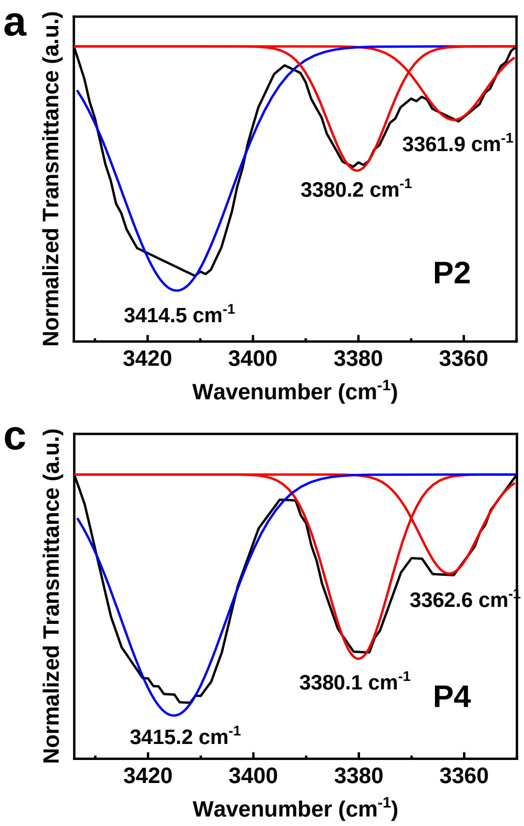

Figure S1. FT-IR analysis of thin films of polymer P2–P5 (a-d). FT-IR spectra (solid black line) and curve fitting (solid blue line and solid red line) of the thin films of polymer P2–P5 in the region of the amide N-H stretching peaks.

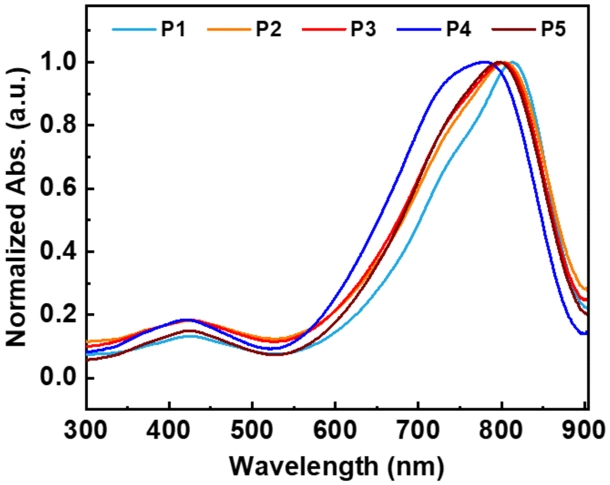

Figure S2. Normalized UV–vis/NIR absorption spectra of polymer P1–P5 in solution state (chloroform).

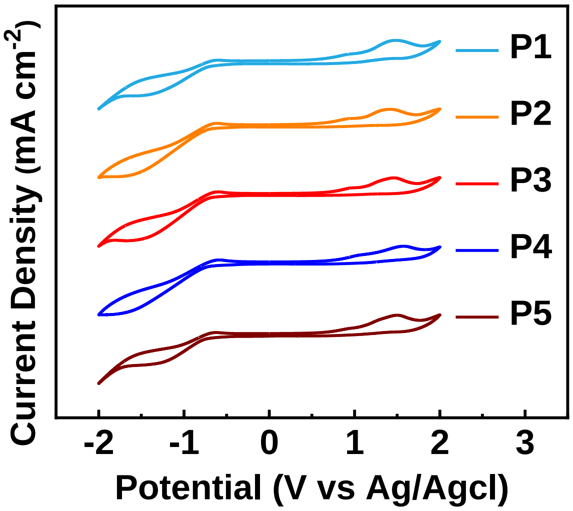

Figure S3. CV curves of polymer (P1–P5) films.

Table S2. The parameters related to the optical absorption, band gaps and thermal stability analysis of P1–P5.

Polymer	E_{ox} (eV)	E_{red} (eV)	HOMO (eV)	LUMO (eV)	$E_{g,cv}$ (eV)	λ_{max} (nm)	λ_{onset} (nm)	$E_{g,opt}$ (eV)	T_g (°C)
**P1**	0.94	-0.67	-5.31	-3.70	1.61	813	916	1.35	444
**P2**	0.92	-0.74	-5.29	-3.63	1.66	803	926	1.34	441
**P3**	0.91	-0.73	-5.28	-3.64	1.64	799	919	1.35	392
**P4**	0.86	-0.73	-5.23	-3.64	1.59	779	905	1.37	387
**P5**	0.91	-0.73	-5.28	-3.64	1.64	797	916	1.35	408

2. Fabrication and characterization of rigid OFETs

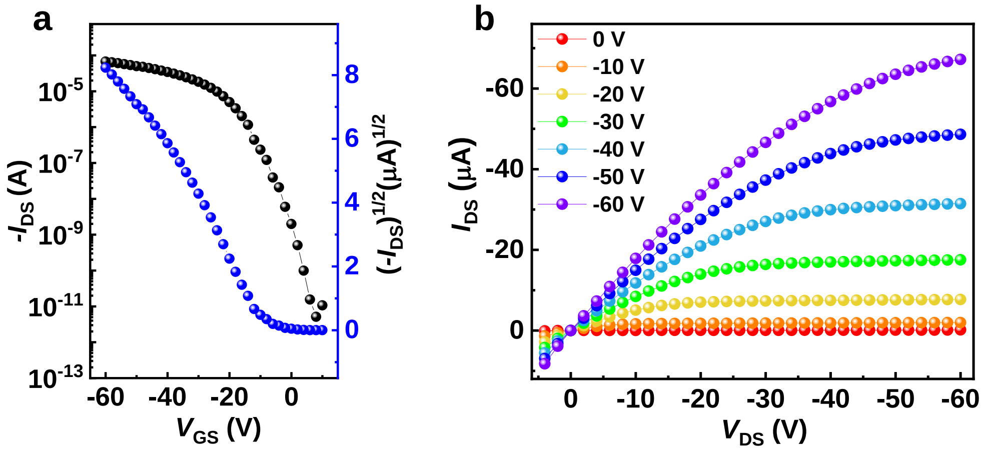

Figure S4. **Typical transfer (a) and output (b) curves for OFET devices based on P1**.

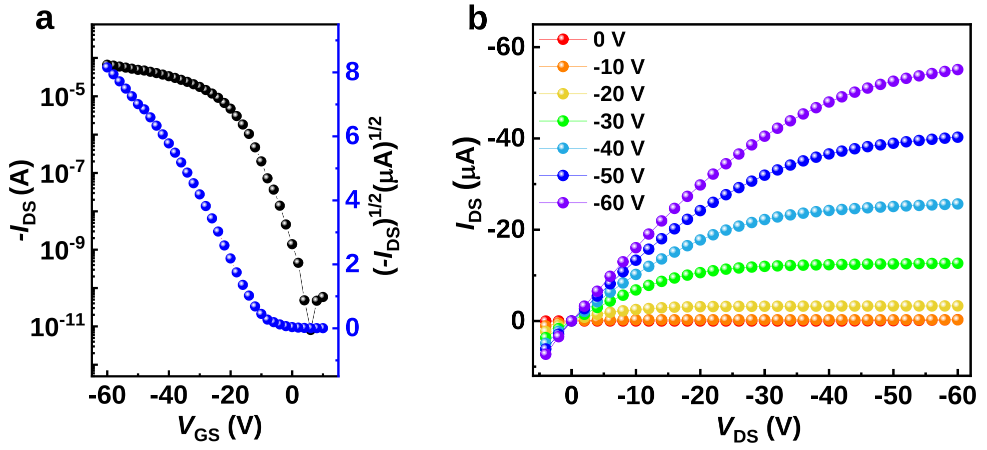

Figure S5. **Typical transfer (a) and output (b) curves for OFET devices based on P2.**

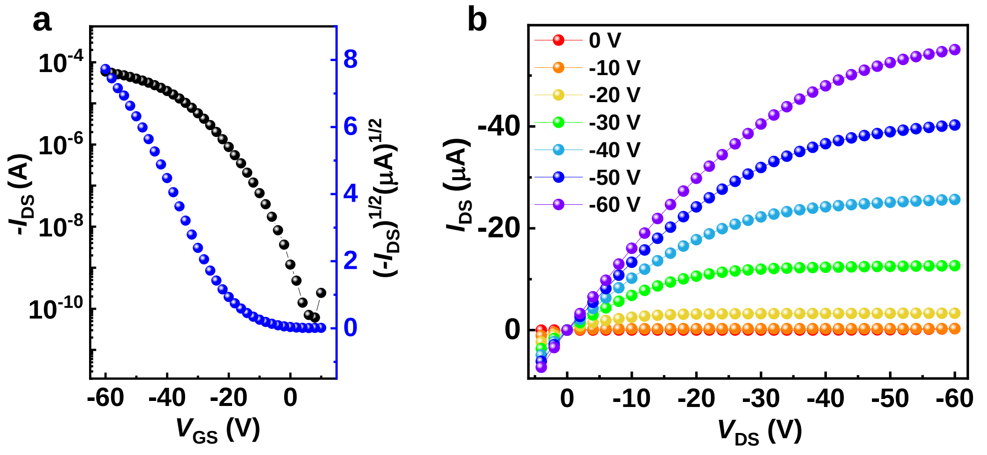

Figure S6. **Typical transfer (a) and output (b) curves for OFET devices based on P3**.

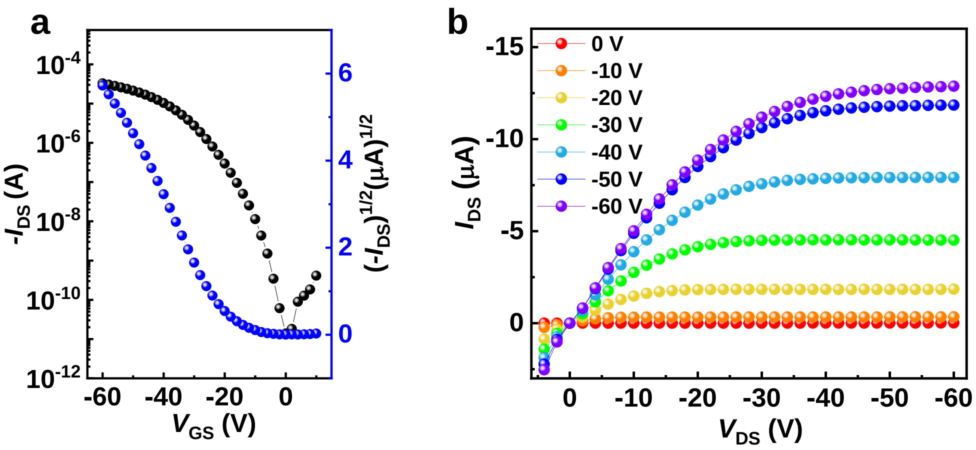

Figure S7. **Typical transfer (a) and output (b) curves for OFET devices based on P4**.

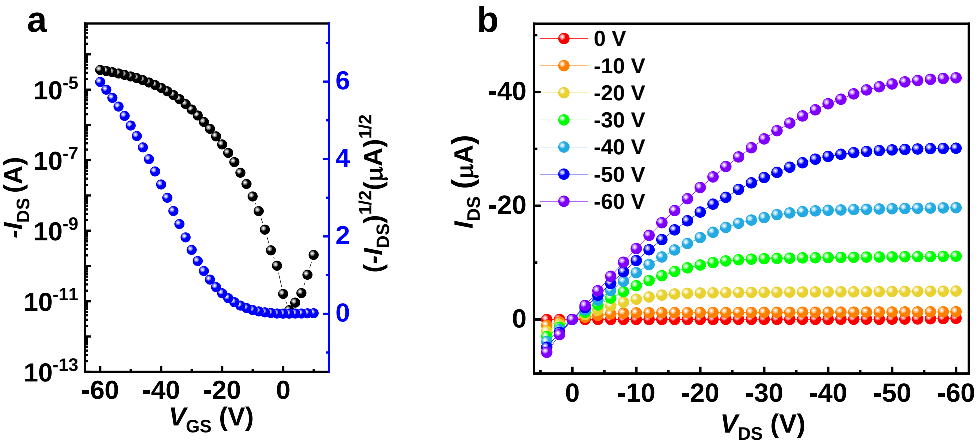

Figure S8. **Typical transfer (a) and output (b) curves for OFET devices based on P5**.

Table S3. OFETs parameters of thin films of polymer P1–P5.

Strain	μ_{ave} (cm^2 V^{-1} s^{-1})	I_{on}/I_{off}	*r* (%)
P1	1.067 ± 0.046		90.73 ± 1.95
P2	1.036 ± 0.035		89.39 ± 1.73
P3	1.006 ± 0.025	10^5–10^6	90.82 ± 1.87
P4	0.589 ± 0.033		89.67 ± 1.79
P5	0.679 ± 0.029		88.34 ± 1.89

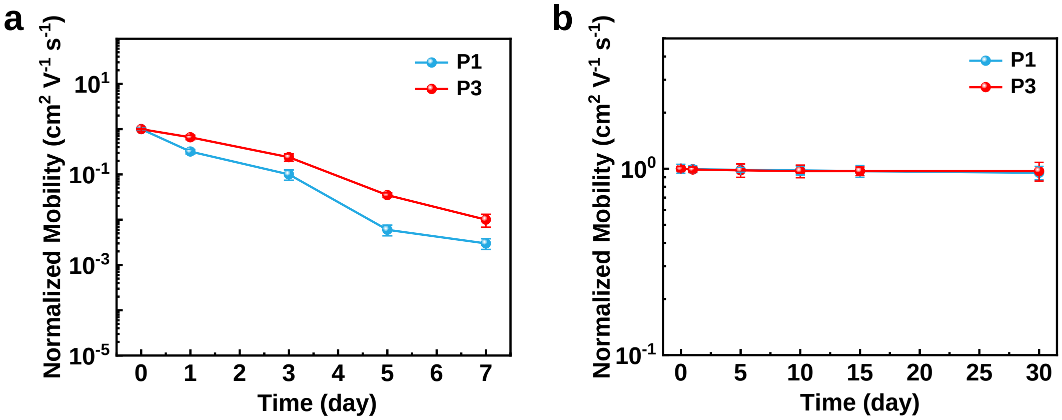

Figure S9. **Electrical performance tests of polymers P1 and P3 in a humidity chamber (a) or a dry nitrogen atmosphere (b).**

3. Single crystal X-rays Diffraction

Table S4. Crystallographic data for compound DCPDCA

Compound	**DCPDCA**
CCDC	**2409200**
Empirical formula	$C_{21}H_{21}N_5O_4S_2$
Formula weight	471.10
Temperature/K	170.0(3)
Crystal description	Needle
Detector type	HyPix
Radiation type	Cu K\a
Radiation wavelength	1.54184
a/Å	9.6407(4)
b/Å	15.5622(5)
c/Å	16.1443(4)
α/°	65.352(3)
β/°	80.626(3)
γ/°	79.511(3)
Z	1
Z'	0.5
d min (Cu K\a)	0.79
R factor all	0.0822
R factor gt	0.0732
Goof	1.054
Final R index	R1=0.0732, wR2=0.2209

a

b

c

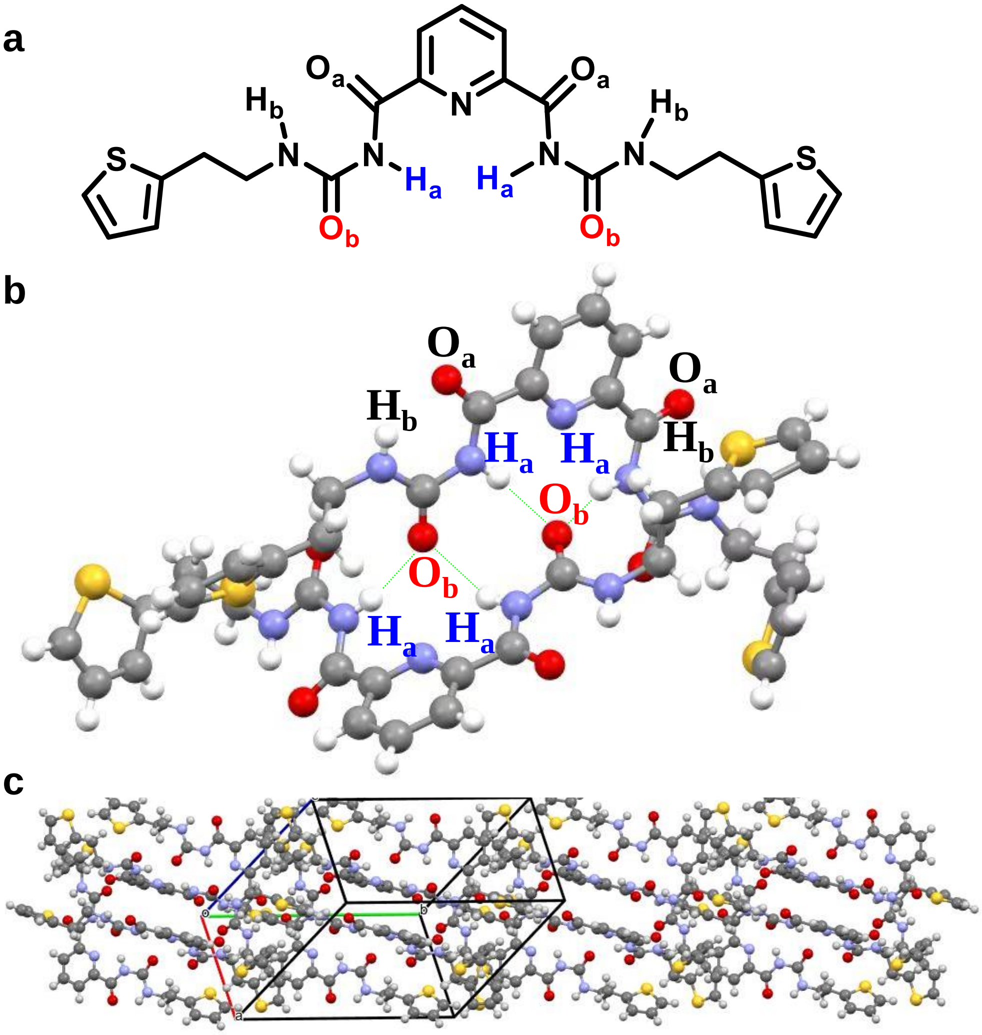

Figure S10. **Molecular structures of DCPDCA showing intermolecular hydrogen bonds determined by single-crystal X-ray diffraction**. **(a)** Chemical structure of DCPDCA. **(b)** Intermolecular hydrogen bonding interactions between two adjacent DCPDCA molecules. **(c)** Part of the crystal structure of DCPDCA showing molecules linked in chains along the *c* axis by N-H···O interaction.

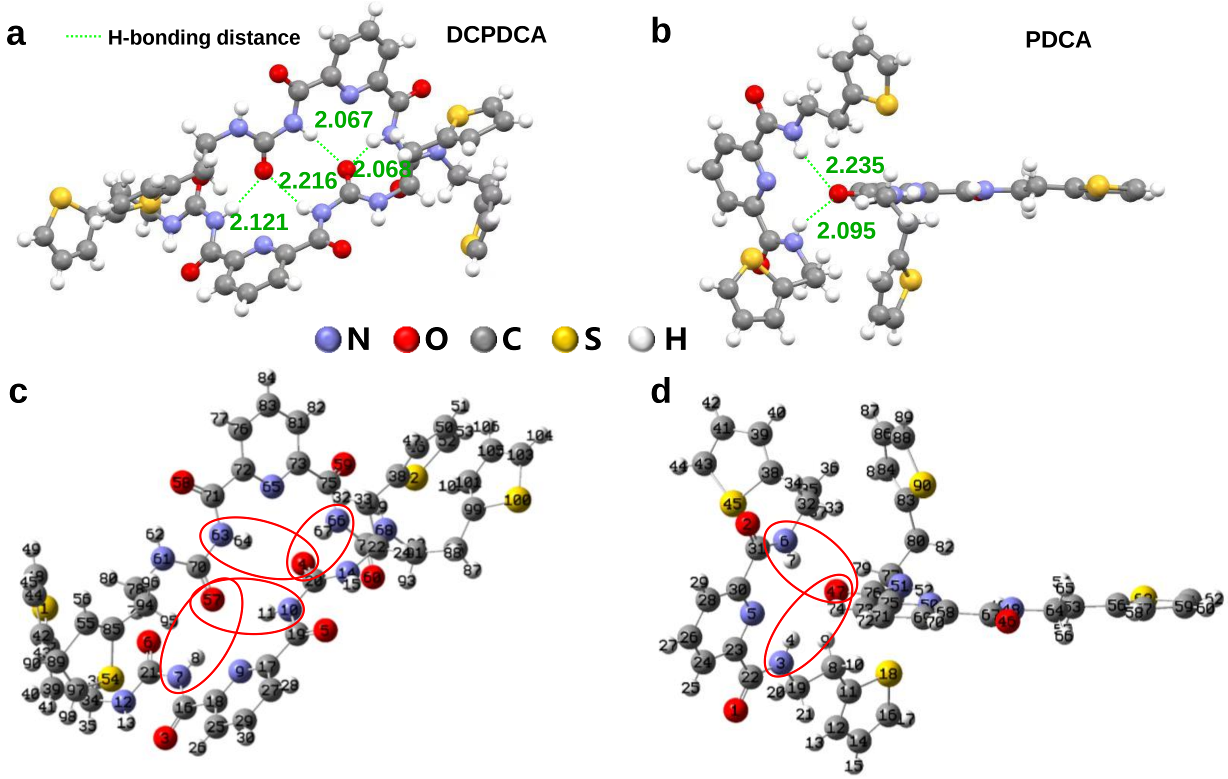

Figure S11. **Hydrogen bonding distance and DFT calculation for DCPDCA and PDCA crystals**. The hydrogen bonding distance for DCPDCA crystal (**a**) and PDCA crystal (**b**). The molecular structure for DCPDCA crystal (**c**) and PDCA crystal (**d**) as extracted from DFT calculation (m08/6-31g**).

Table S5. The distance of hydrogen bonding and hydrogen bonding energy for DCPDCA and PDCA crystals.

Compound	N	H	O	D(H-O)[Å]	D(N-O)[Å]	N-H-O[°]	Energy[Kcal/mol]
DCPDCA	N7	H8	O57	2.068	2.886	162.93	6.51
DCPDCA	N10	H11	O57	2.067	2.909	156.20	9.42
DCPDCA	N63	H64	O4	2.216	2.976	150.93	6.94
DCPDCA	N66	H67	O4	2.121	2.982	170.00	2.13
PDCA	N6	H7	O47	2.095	2.847	149.21	5.11
PDCA	N3	H4	O47	2.235	3.059	158.98	2.18

4. Characterization of intermolecular hydrogen bonds

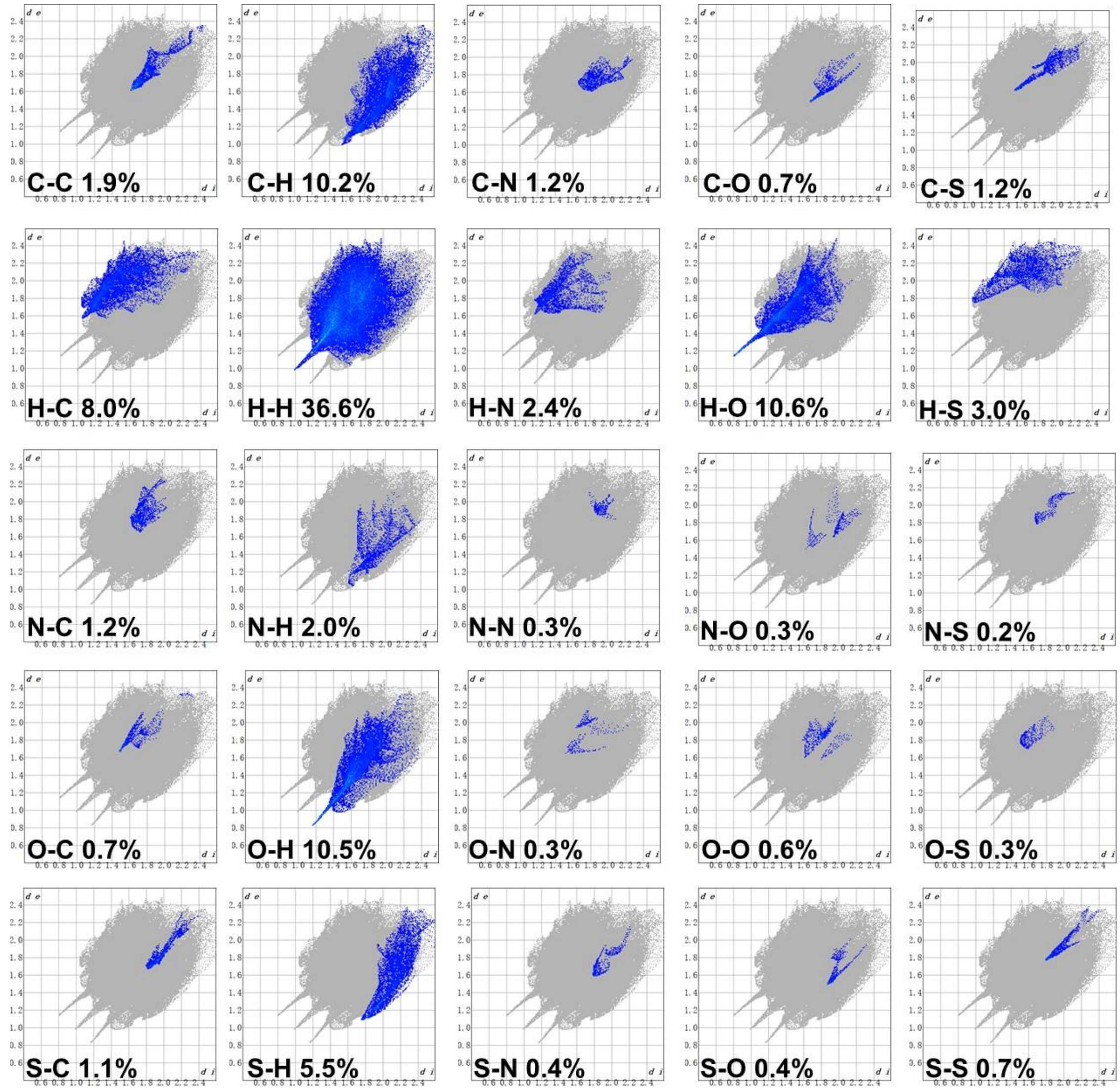

Figure S12. **Fingerprint plots for different contacts in DCPDCA crystal**.

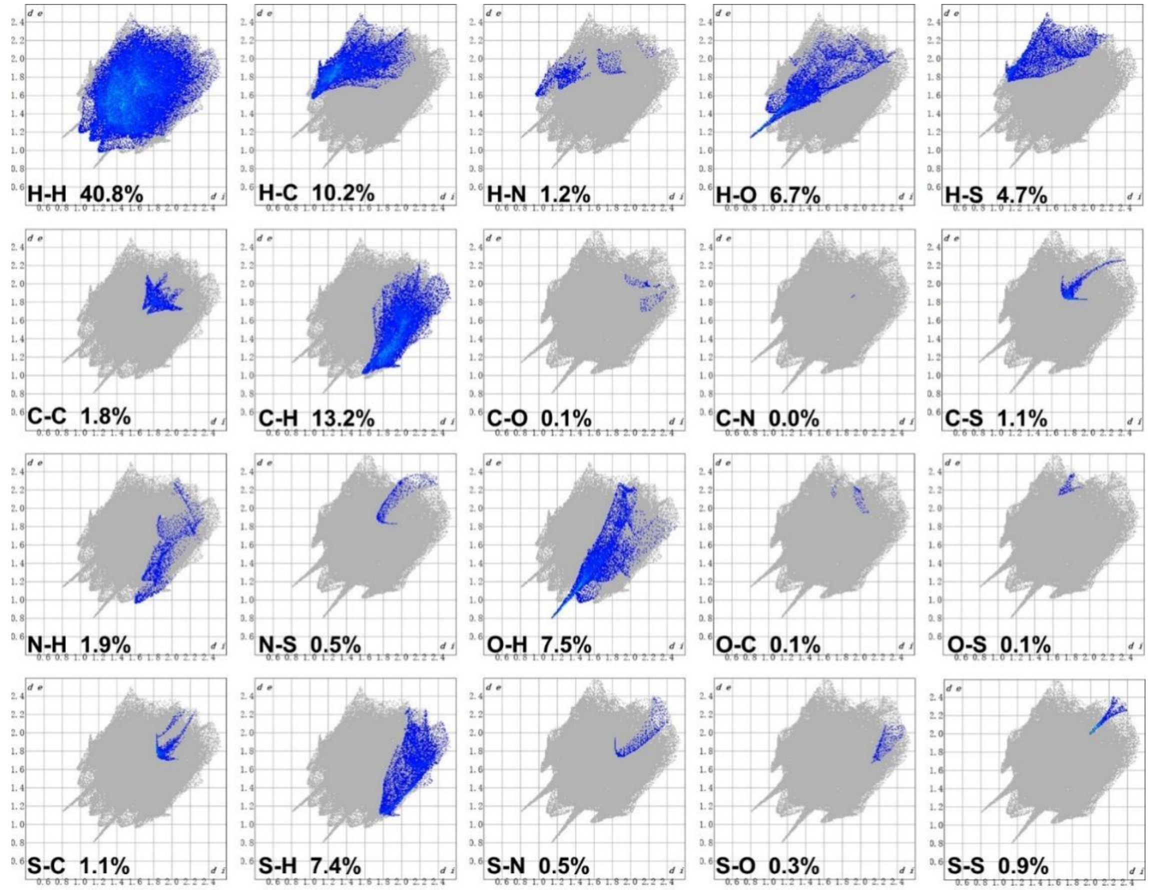

Figure S13. **Fingerprint plots for different contacts in PDCA crystal**.

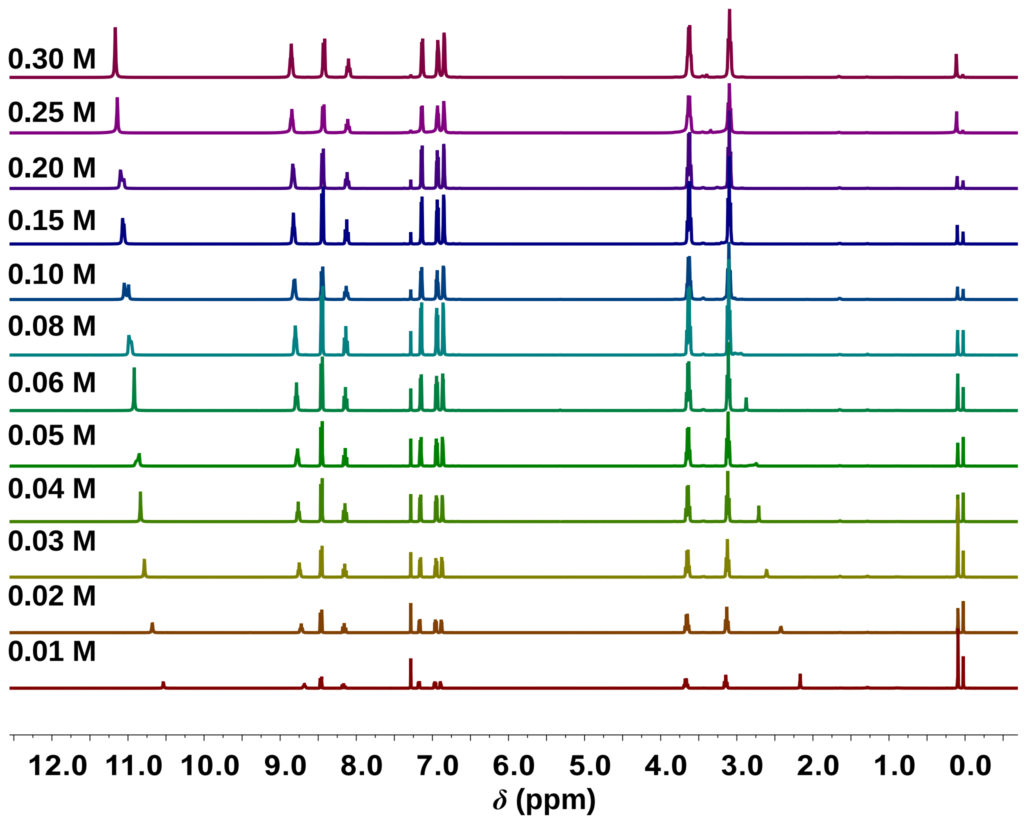

Figure S14. [1]**H NMR spectra of DCPDCA with different concentrations (0.01M–0.30M) in $CDCl_3$ (400 MHz)**.

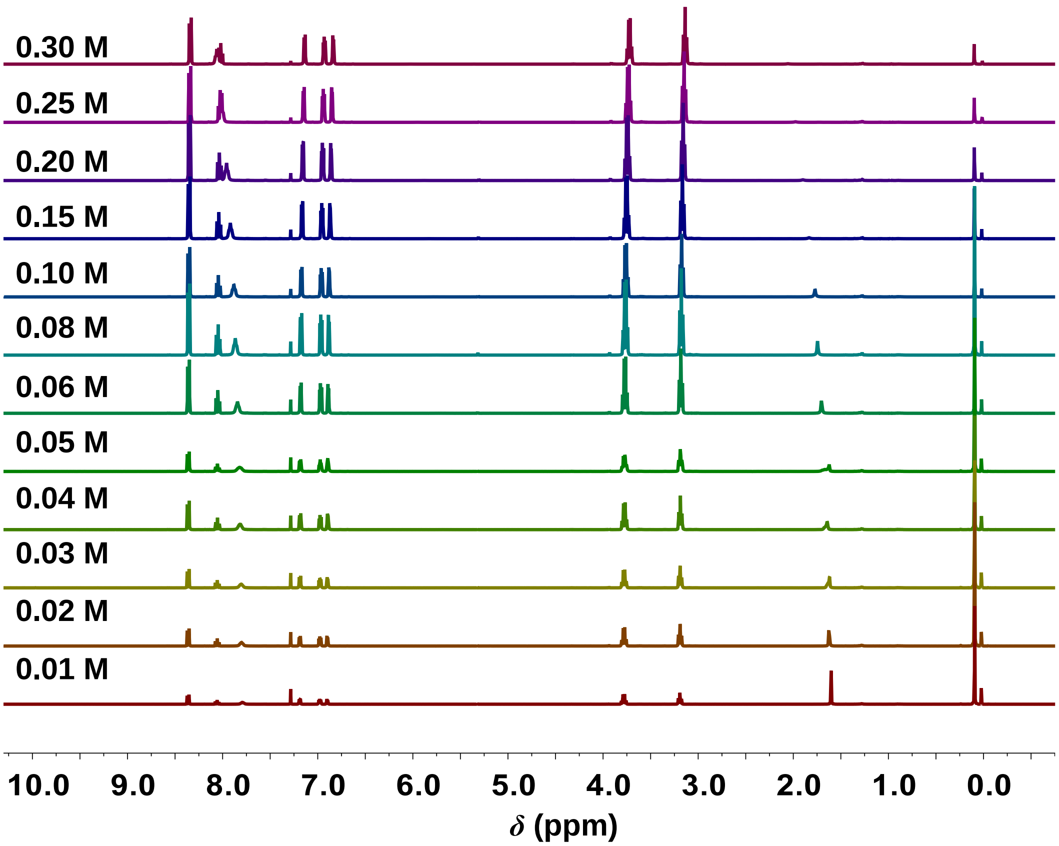

Figure S15. **^1H NMR spectra of PDCA with different concentrations (0.01M–0.30M) in $CDCl_3$ (400 MHz)**.

Table S6. **The related parameters extract the self-association constant equation**.

Model	DCPDCA N-H_a	DCPDCA N-H_b	PDCA N-H
Equation	C+(C-A)/(4*K*X)*(1-sqrt(1+8*K*X))		
C	11.4057 ± 0.0208	8.9294 ± 0.0089	8.9243 ± 0.2659
A	10.0151 ± 0.0986	8.5825 ± 0.0253	7.7688 ± 0.0064
K	45.6951 ± 11.7625	28.7299 ± 8.7672	0.5916 ± 0.2123
Reduced Chi-Sqr	1.4183E-4	2.1332E-5	5.5255E-5
R-Square (COD)	0.9970	0.9939	0.9930
Adj. R-Square	0.9963	0.9925	0.9914

5. Thin film morphologies of polymer semiconductors under mechanical deformation

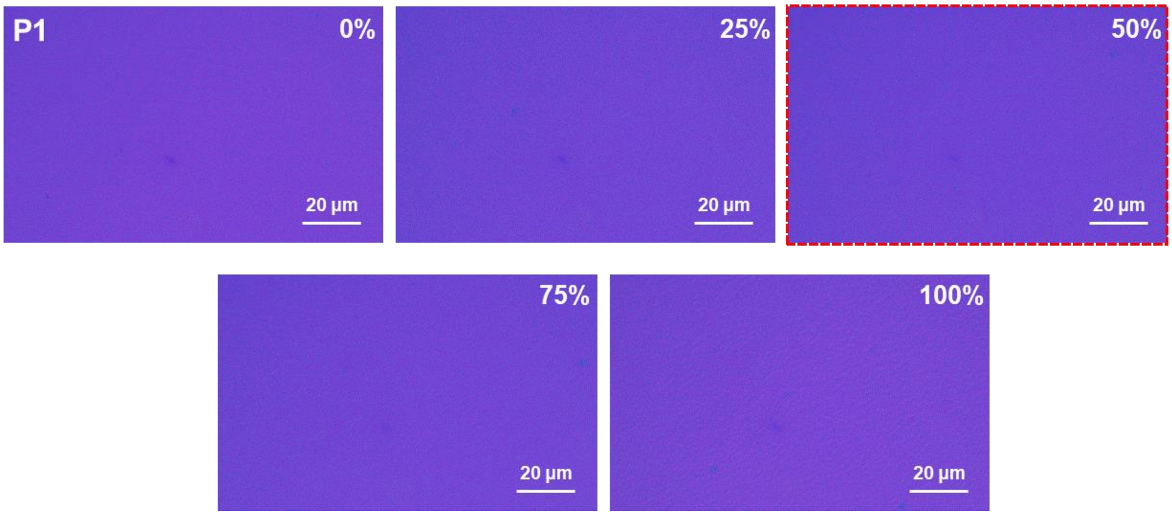

Figure S16. Optical microscopic images of P1 films transferred onto the PDMS substrate under different strains. (Scale bar: 20 µm).

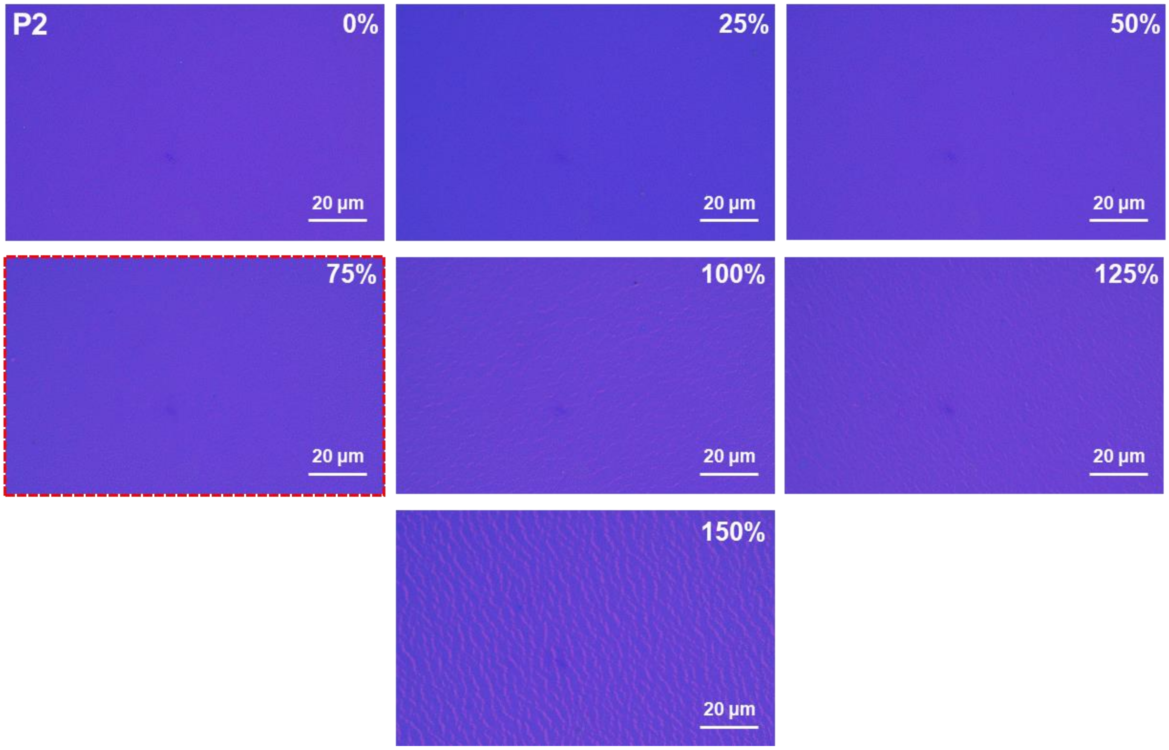

Figure S17. Optical microscopic images of P2 films transferred onto the PDMS substrate under different strains. (Scale bar: 20 µm).

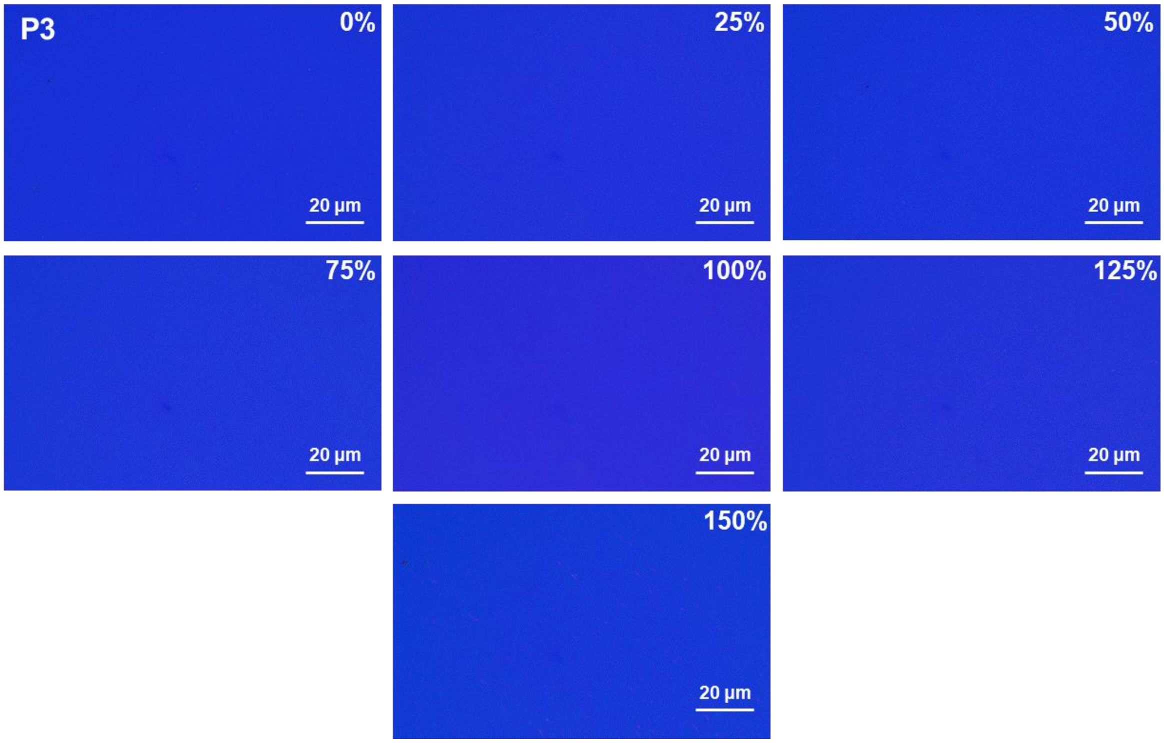

Figure S18. **Optical microscopic images of P3 films transferred onto the PDMS substrate under different strains**. **(Scale bar: 20 µm)**.

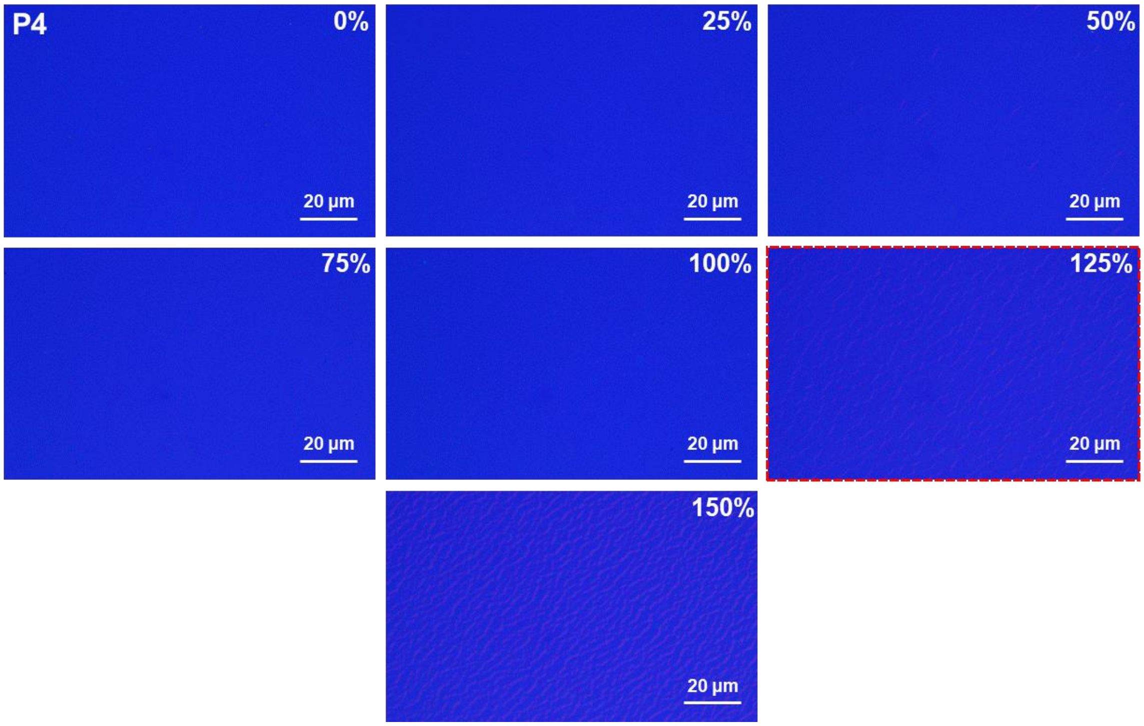

Figure S19. **Optical microscopic images of P4 films transferred onto the PDMS substrate under different strains**. **(Scale bar: 20 µm)**.

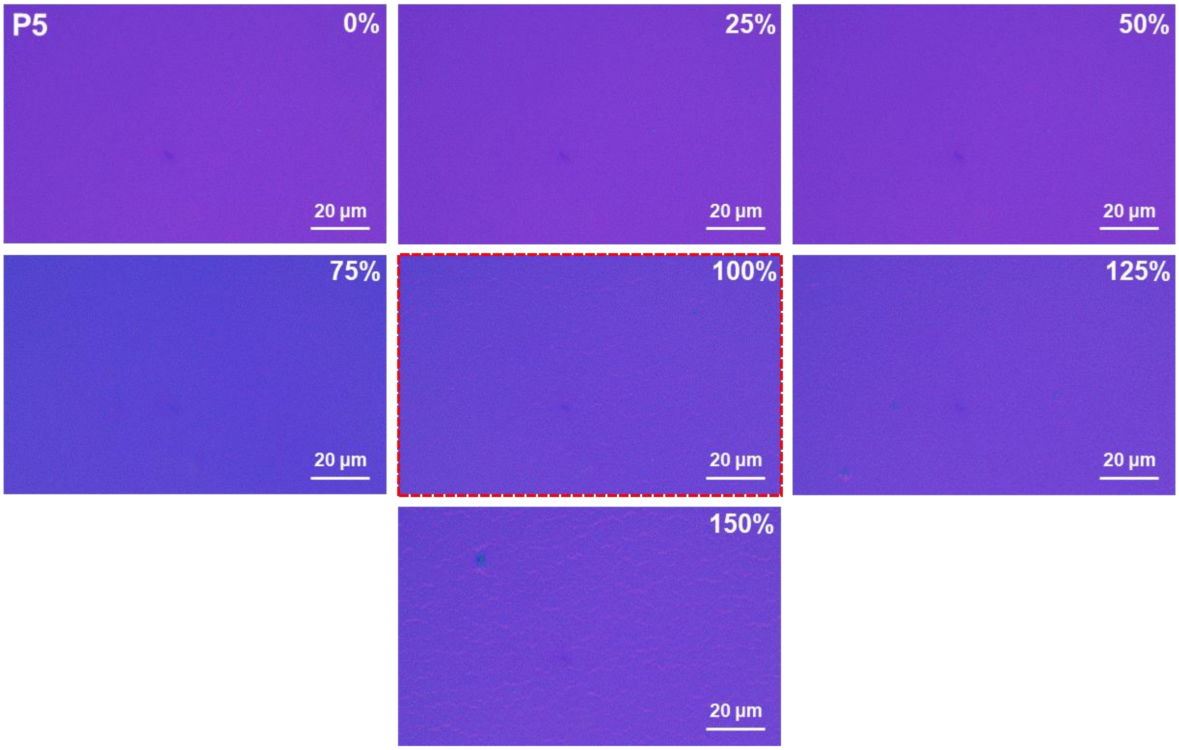

Figure S20. **Optical microscopic images of P5 films transferred onto the PDMS substrate under different strains**. **(Scale bar: 20 µm)**.

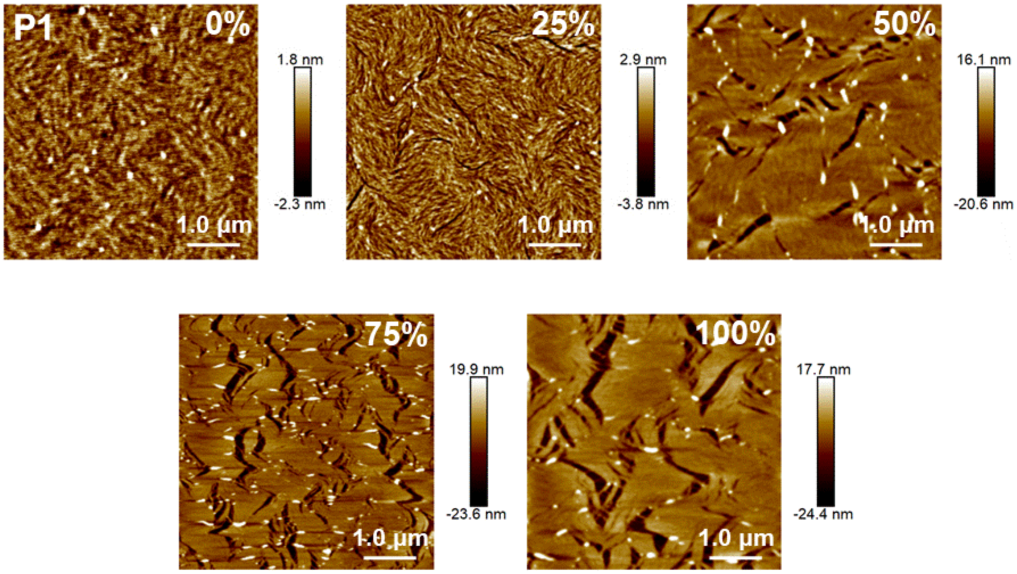

Figure S21. **AFM height images of P1 stretched thin films under different strains (Scale bar: 1.0 µm)**.

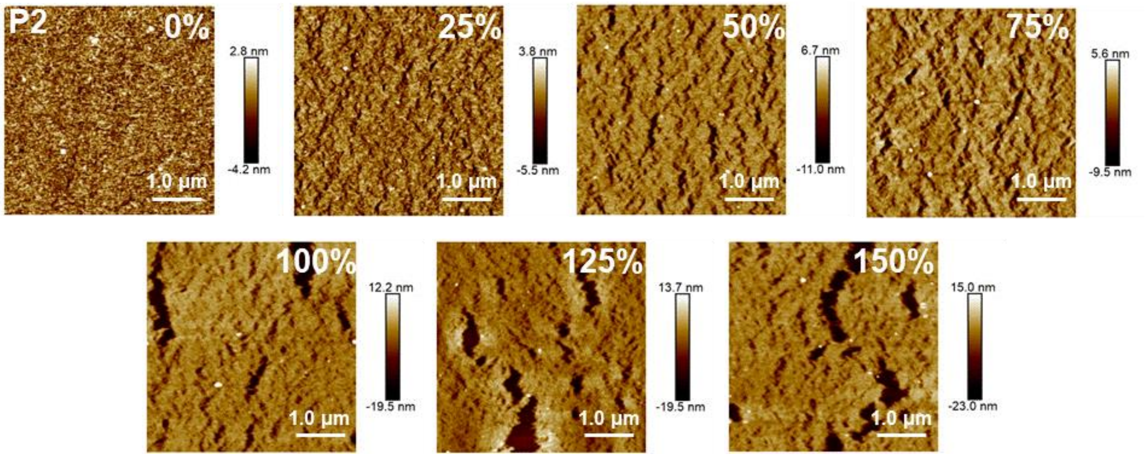

Figure S22. AFM height images of P2 stretched thin-films under different strains (Scale bar: 1.0 μm).

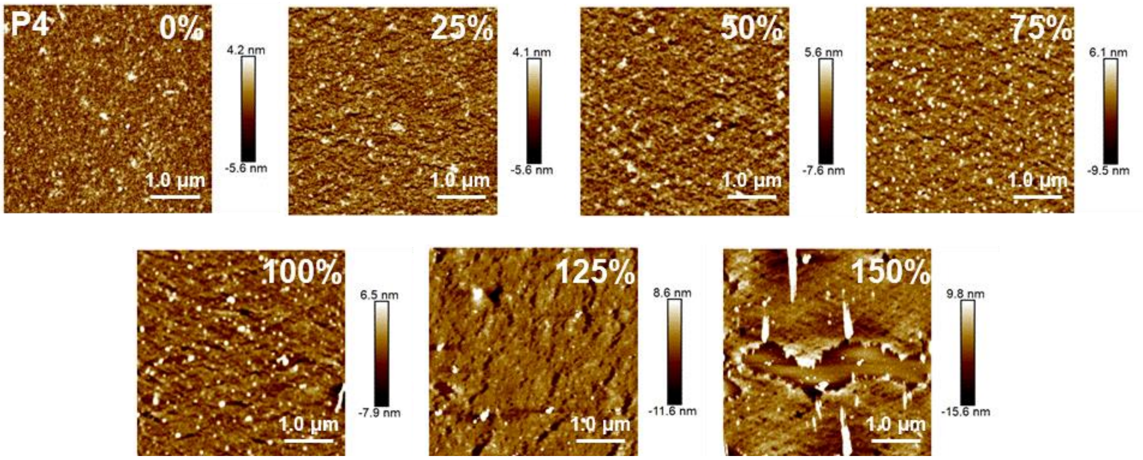

Figure S23. AFM height images of P4 stretched thin-films under different strains (Scale bar: 1.0 μm).

6. Dichroic ratios, moduli and crystallinity properties of polymer semiconductors

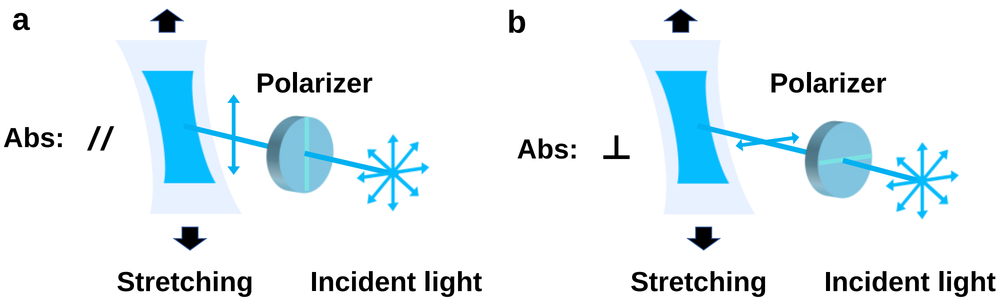

Figure S24. Schematic illustration of polarized UV-Vis/NIR spectra for the stretched polymer films along the parallel (a) and perpendicular (b) directions.

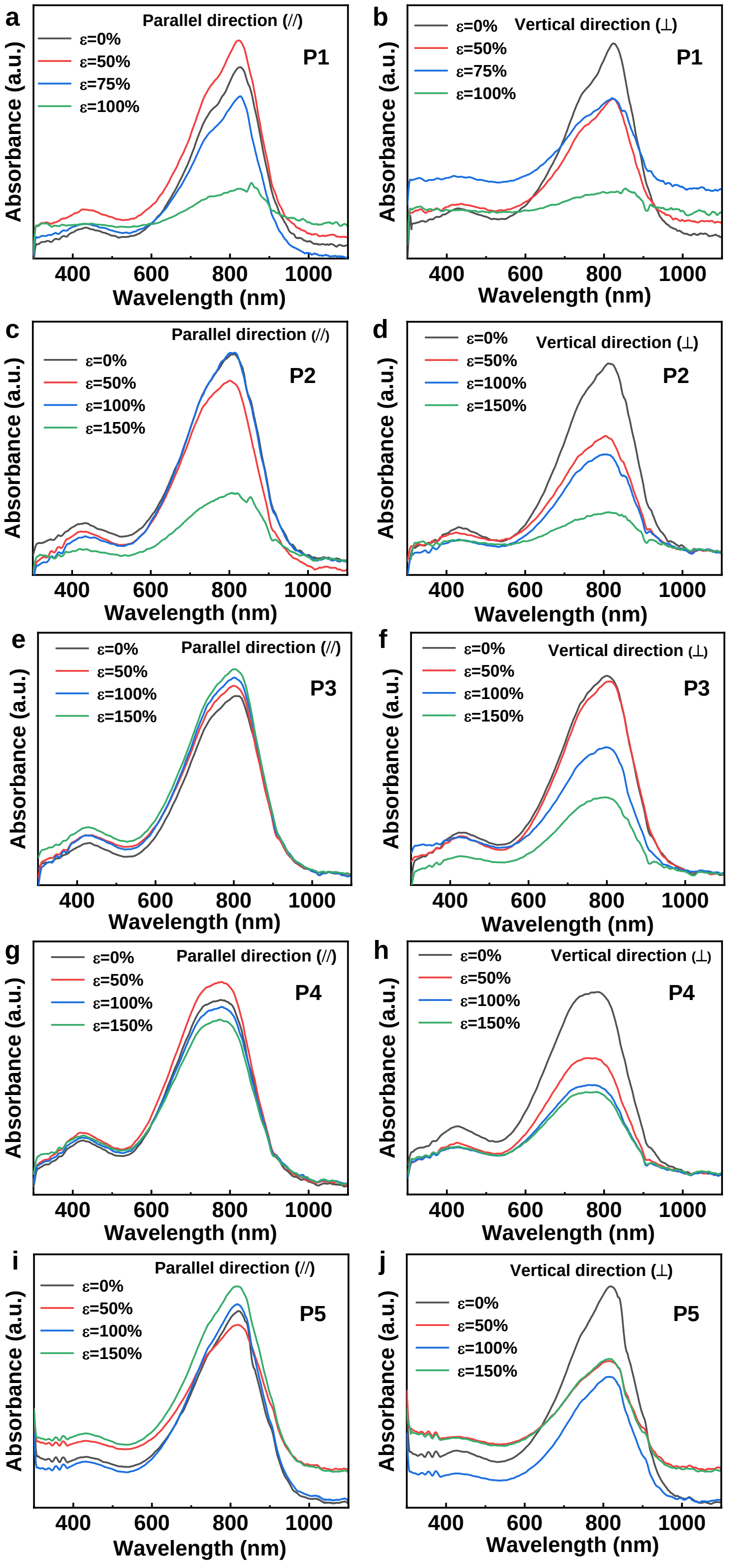

Figure S25. **Polarized absorption properties**. Polarized UV-Vis/NIR spectra for the stretched films of P1 (**a, b**), P2 (**c, d**), P3 (**e, f**), P4 (**g, h**) and P5 (**i, j**) under the different strains based on the 0-0 absorption peak.

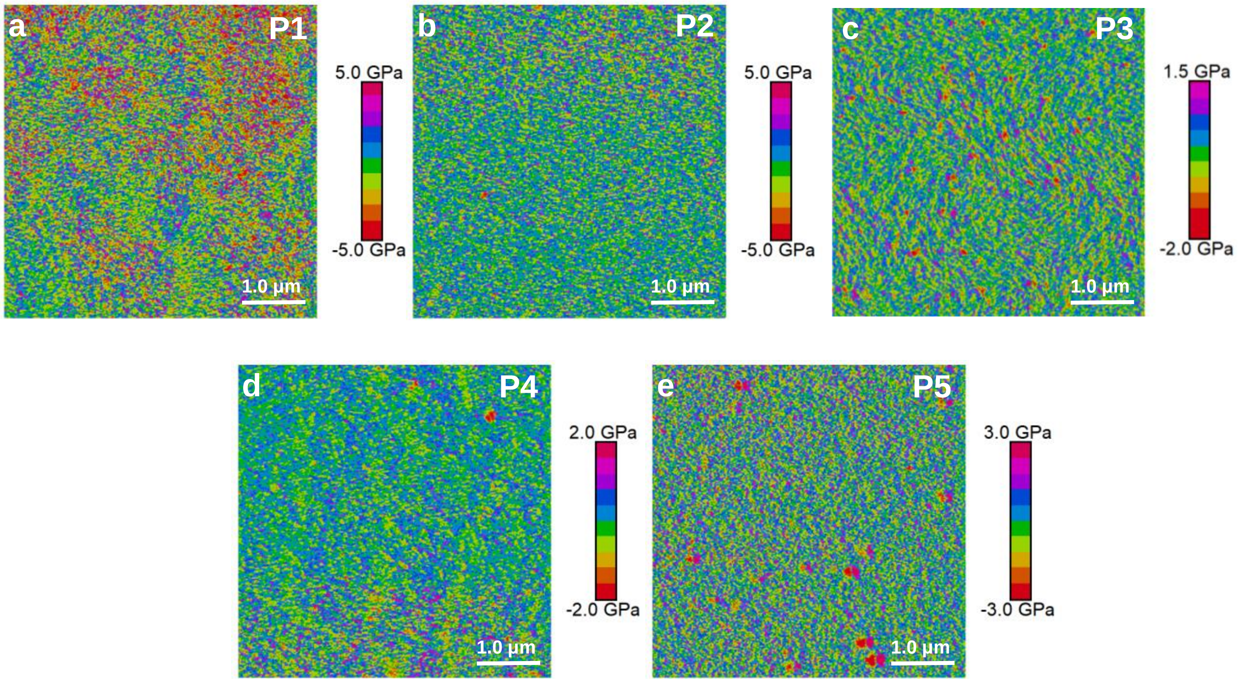

Figure S26. **The tensile moduli by AFM nanomechanical mapping**. The elastic moduli of polymer P1–P5 (**a-e**) by AFM nanomechanical mapping.

Table S7. The parameters related to elastic modulus, toughness and maximum stretchability of P1–P5 derived from stress-strain curves.

Polymer	Elastic modulus (MPa)	Toughness (MJ/m^3)	Maximum stretchability (%)
**P1**	345.1±27.6	0.69±0.04	6.4±0.3
**P2**	246.8±16.5	0.32±0.03	8.2±0.5
**P3**	132.6±9.3	6.04±0.29	50.6±2.7
**P4**	201.5±13.2	0.67±0.05	10.3±0.8
**P5**	180.5±11.7	0.15±0.02	5.3±0.4

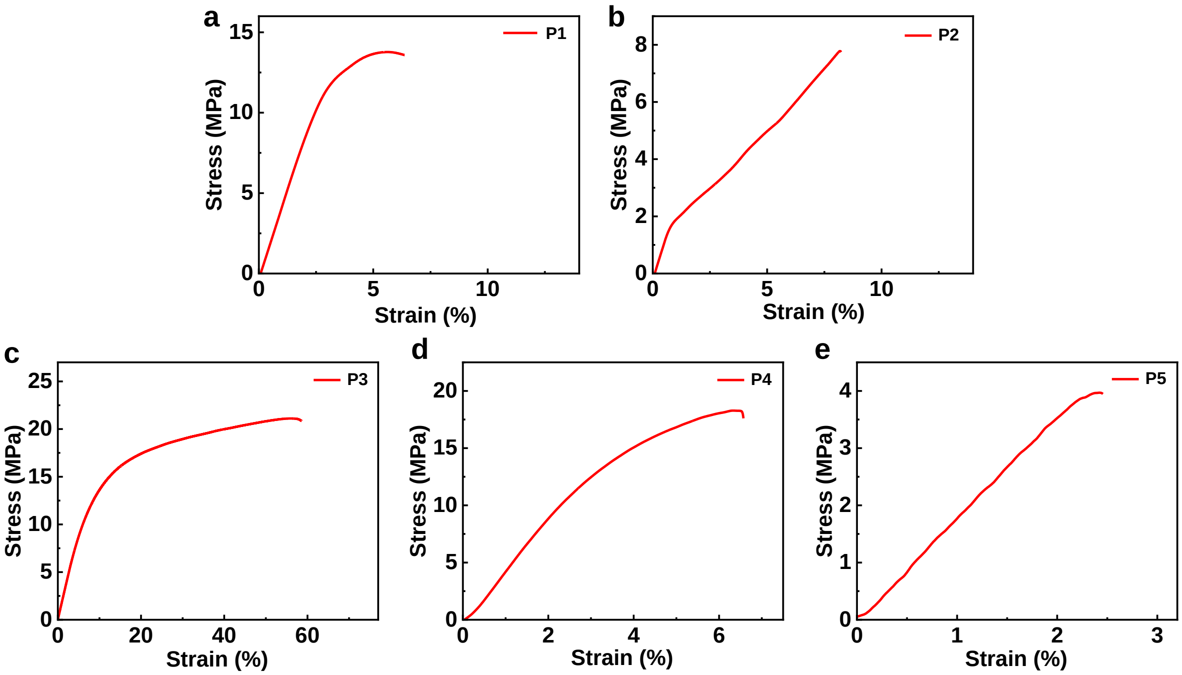

Figure S27. **Stress-strain curves of films based on P1–P5 (a-e)**.

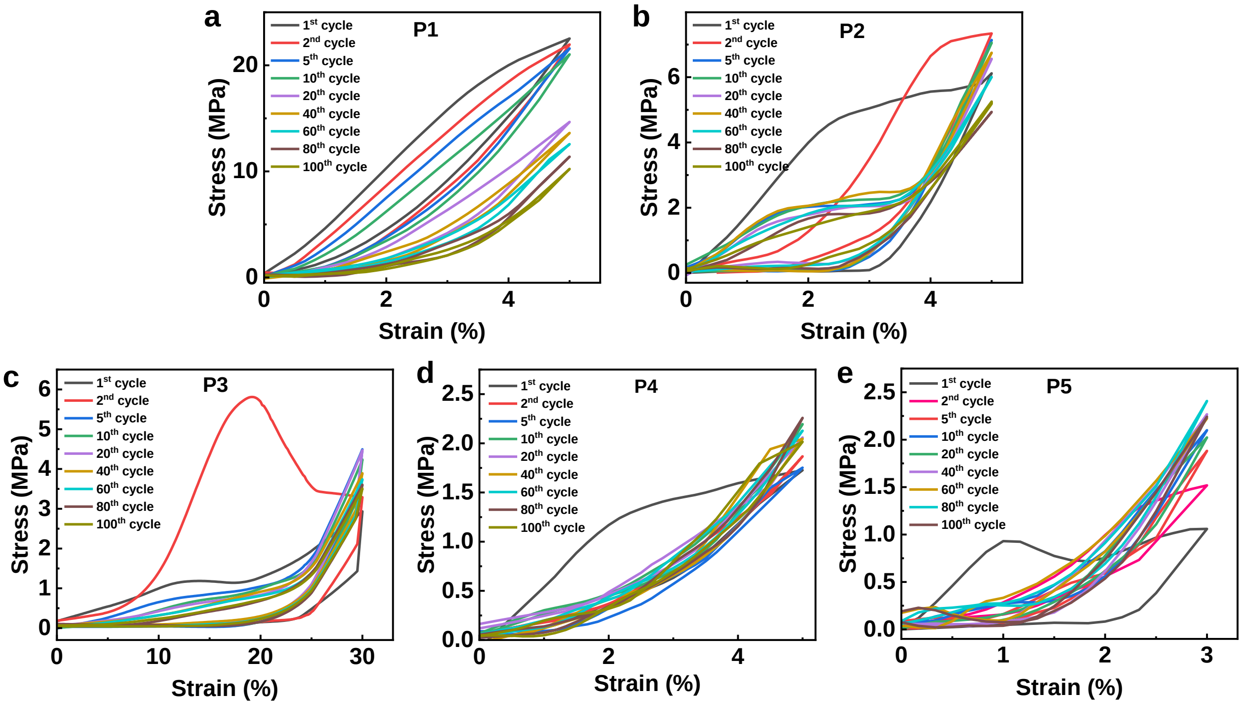

Figure S28. **Typical cyclic stress-strain stretching tests for P1–P5 (a-e).**

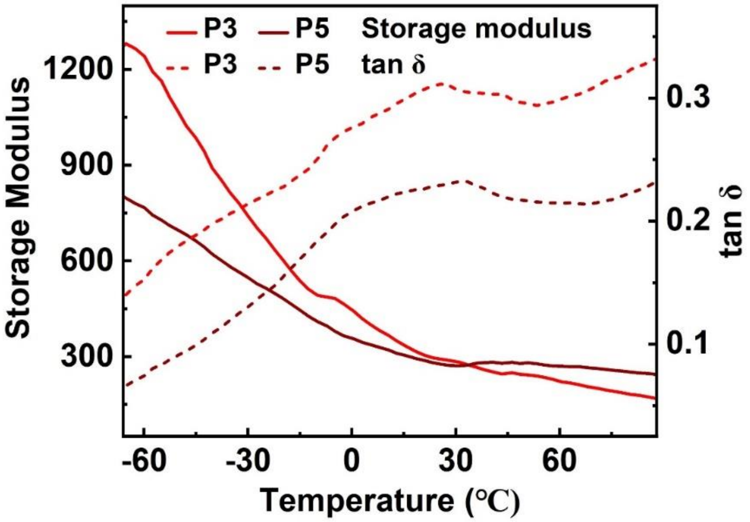

Figure S29. **Storage modulus and tan δ of P3 and P5 as functions of temperature.**

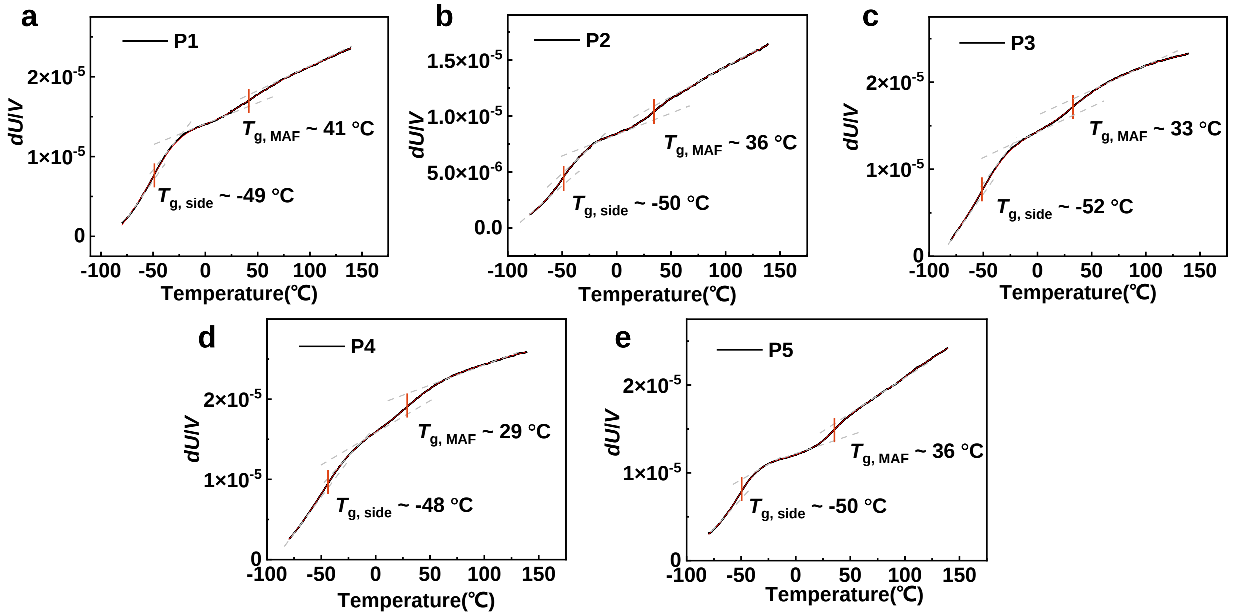

Figure S30. **Heating curves from calorimetric ac-chip measurement for polymer films based on P1–P5 (a-e).**

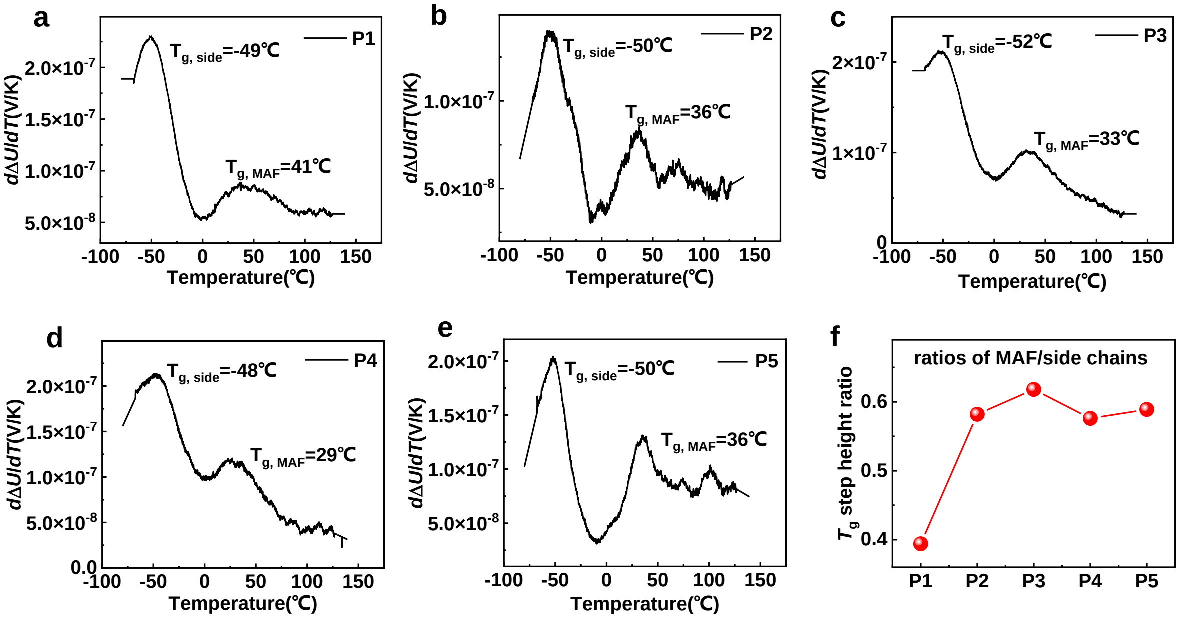

Figure S31. **The first derivative curves from calorimetric ac-chip measurement for polymer films based on P1–P5 (a-e) and the step height ratio of P1–P5 (f) based on the heat capacity at the corresponding glass transitions.**

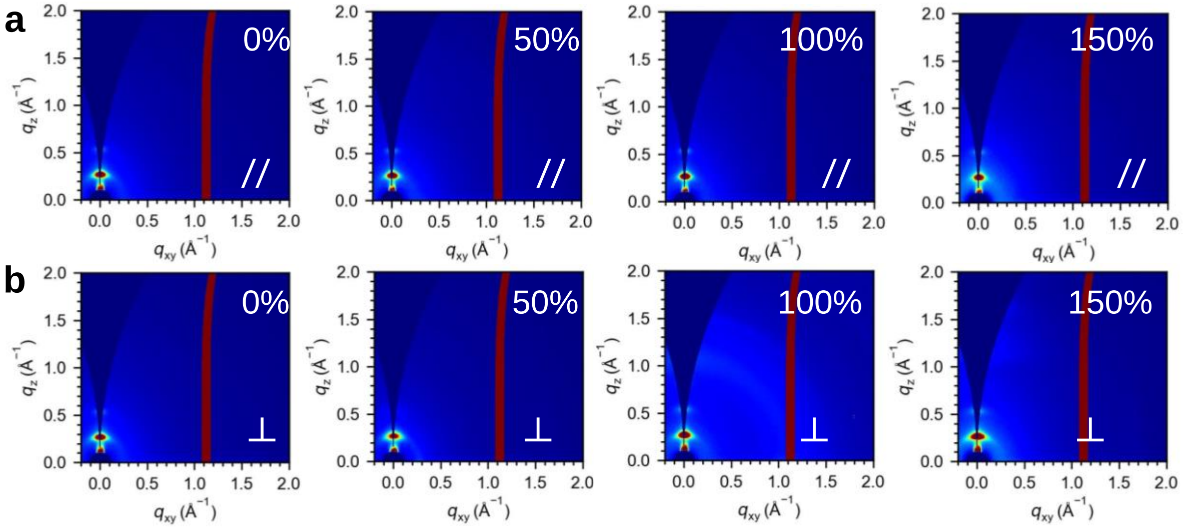

Figure S32. **GIWAXS of P1 films**. The results were obtained under different strains along the parallel (**a**) or perpendicular (**b**) direction of incident X-ray with respect to the strain.

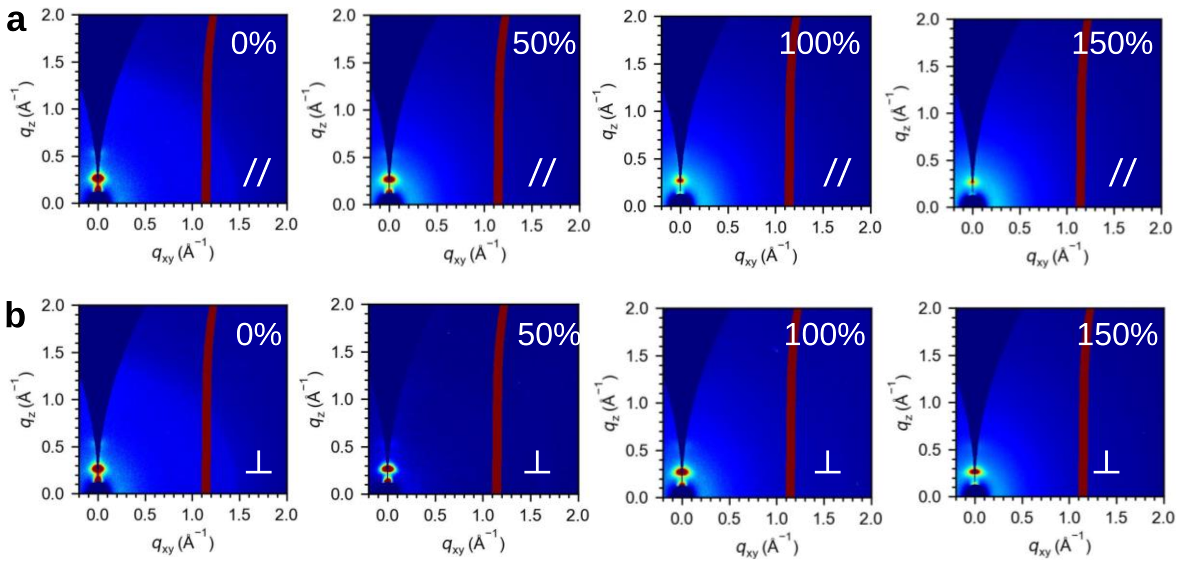

Figure S33. **GIWAXS of P2 films**. The results were obtained under different strains along the parallel (**a**) or perpendicular (**b**) direction of incident X-ray with respect to the strain.

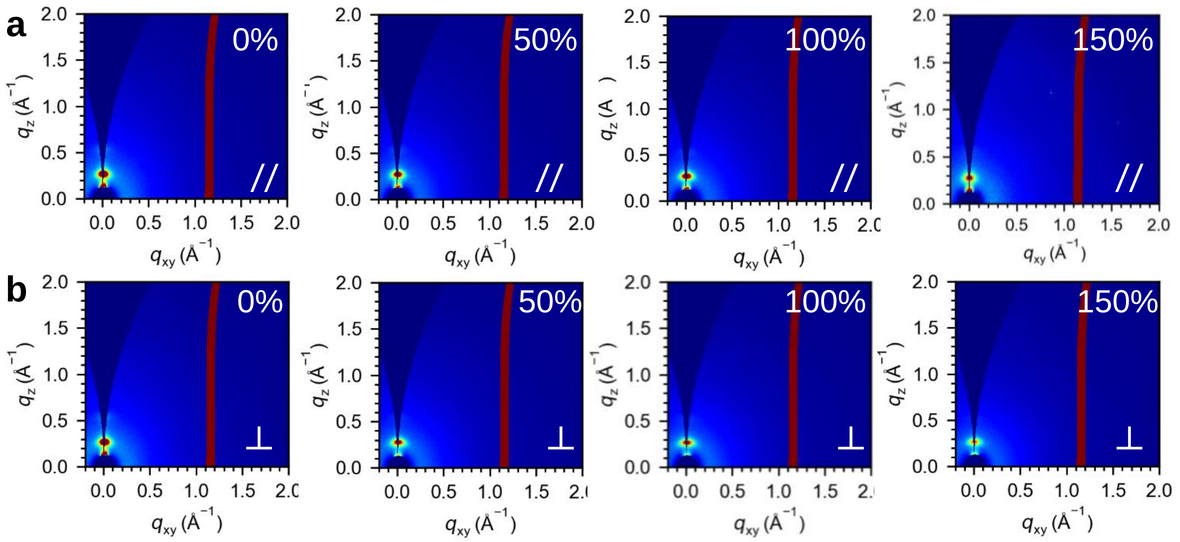

Figure S34. **GIWAXS of P3 films**. The results were obtained under different strains along the parallel (**a**) or perpendicular (**b**) direction of incident X-ray with respect to the strain.

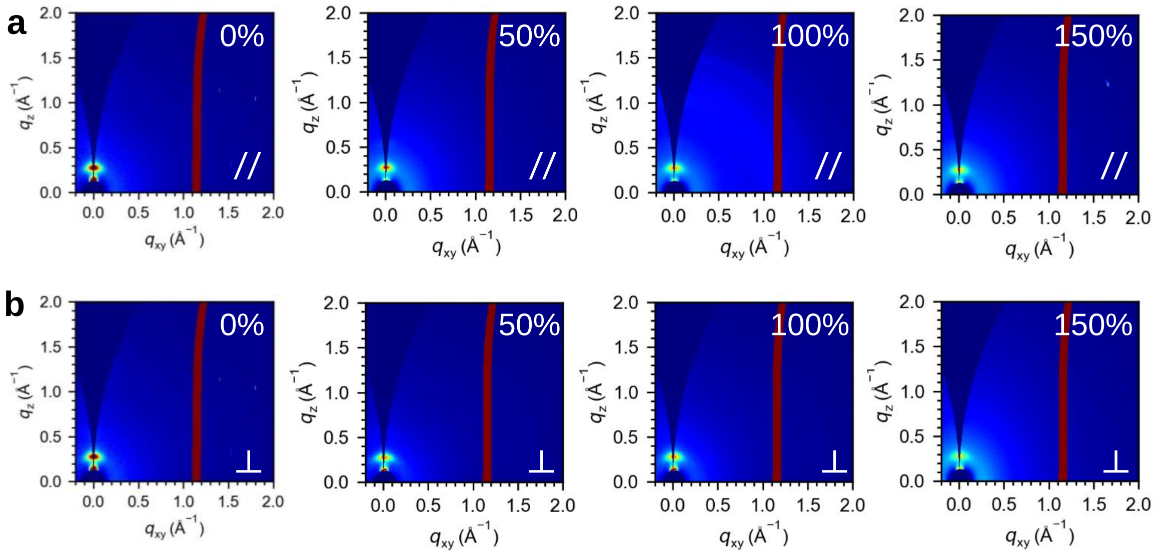

Figure S35. **GIWAXS of P4 films**. The results were obtained under different strains along the parallel (**a**) or perpendicular (**b**) direction of incident X-ray with respect to the strain.

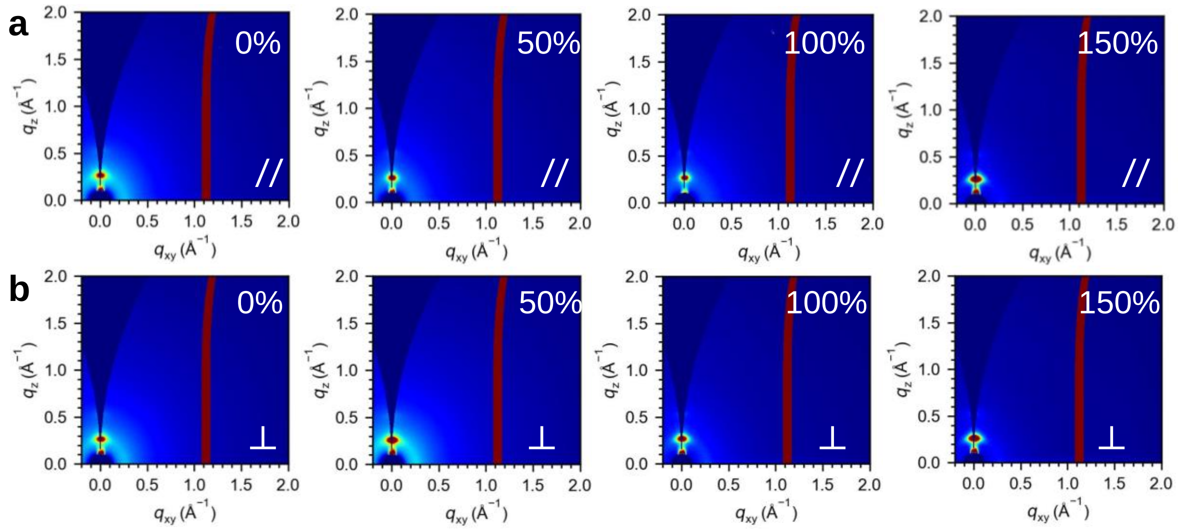

Figure S36. **GIWAXS of P5 films**. The results were obtained under different strains along the parallel (**a**) or perpendicular (**b**) direction of incident X-ray with respect to the strain.

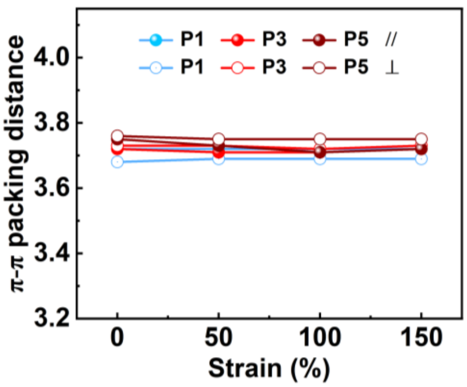

Figure S37. **The changes of relative $\pi-\pi$ stacking distance of P1, P3 and P5 films under different strains**.

Table S8. Detailed parameters of the polymer films as obtained from GIWAXS.

Material	q_z 100	Lamellar spacing [Å] 100	Lamellar peak FWHM [$Å^{-1}$] 100	coherence length [Å] 100	rDOC
**P1**	0.262	23.95	0.05036	112.29	1
**P2**	0.262	23.85	0.06061	93.30	0.91
**P3**	0.268	23.48	0.06493	87.10	0.81
**P4**	0.271	23.16	0.08756	64.58	0.72
**P5**	0.265	23.75	0.06628	85.32	0.84

7. Electrical properties under mechanical deformation

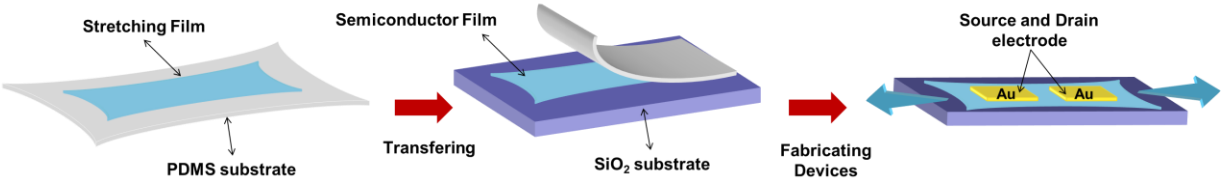

Figure S38. **The Illustration of the OFETs with the stretched thin films of polymers under different strains.**

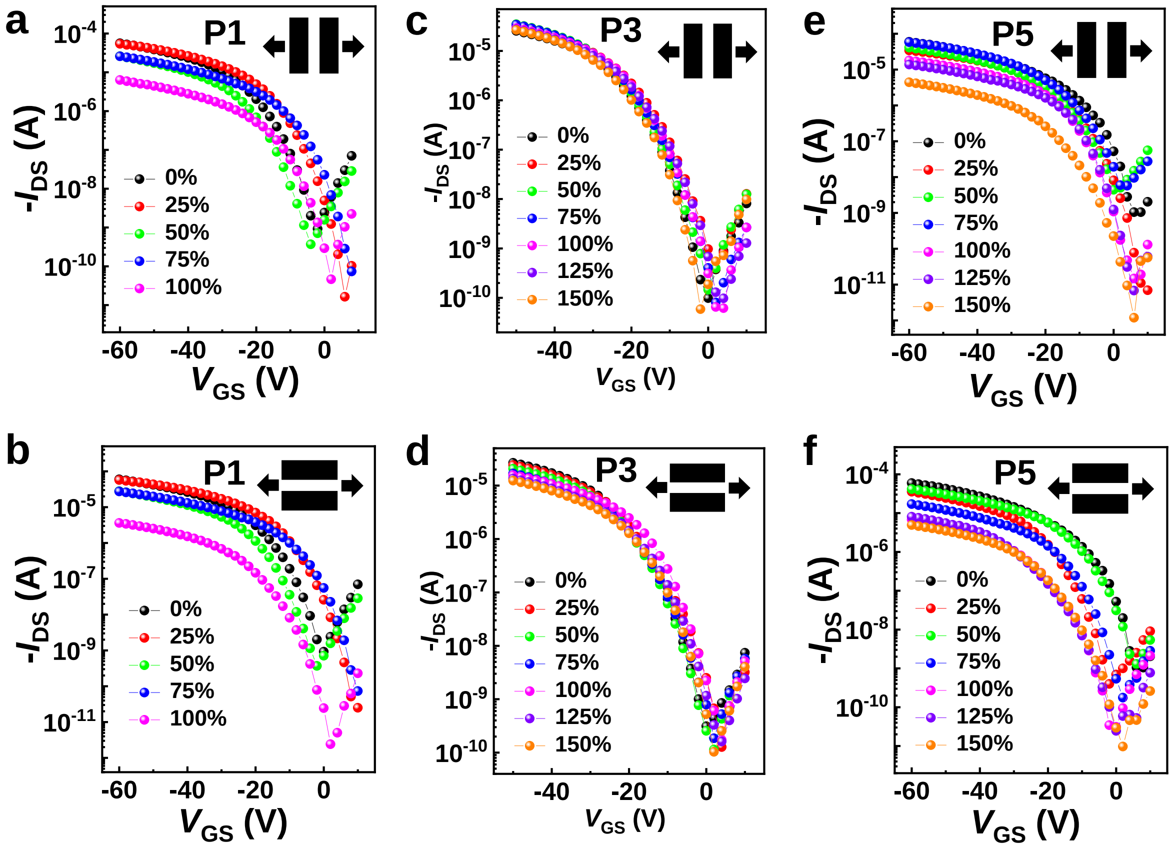

Figure S39. **Electrical characteristics of P1, P3 and P5 films at strains**. Transfer curves of P1, P3 and P5 films at different strains along the parallel (**a**, **c**, **e**) or perpendicular **b** stretching direction (**b**, **d**, **f**).

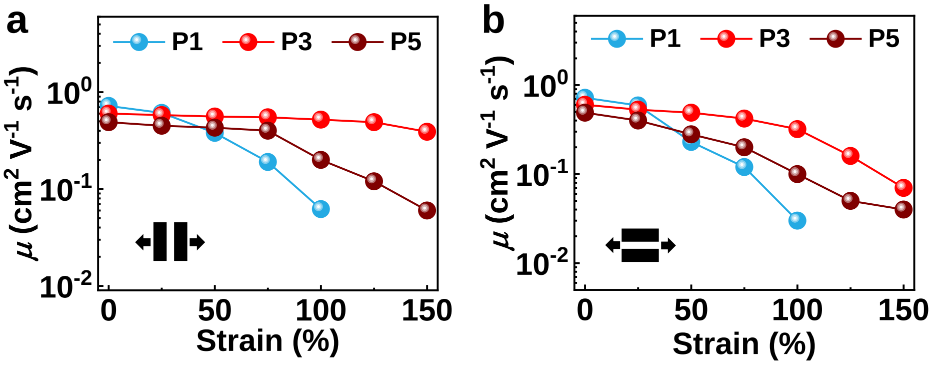

Figure S40. **Field-effect mobilities versus the different strains of P1, P3 and P5 along the parallel (a) and perpendicular (b) stretching direction.**

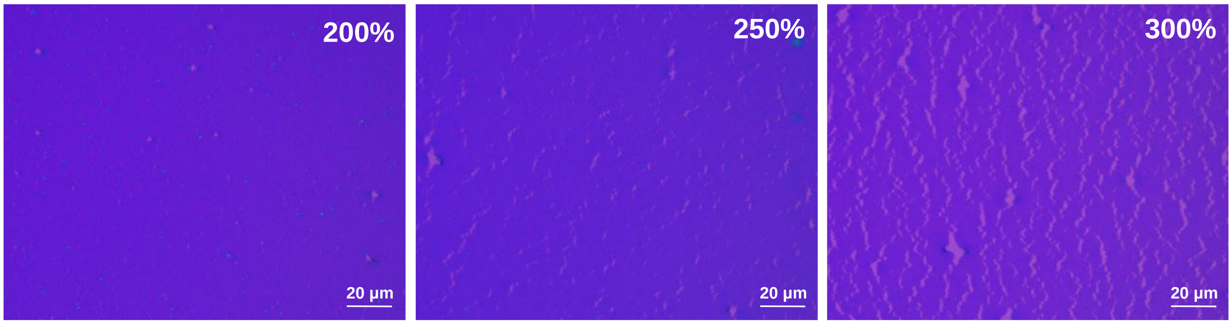

Figure S41. **Optical microscopy images of P3 under different strains**.

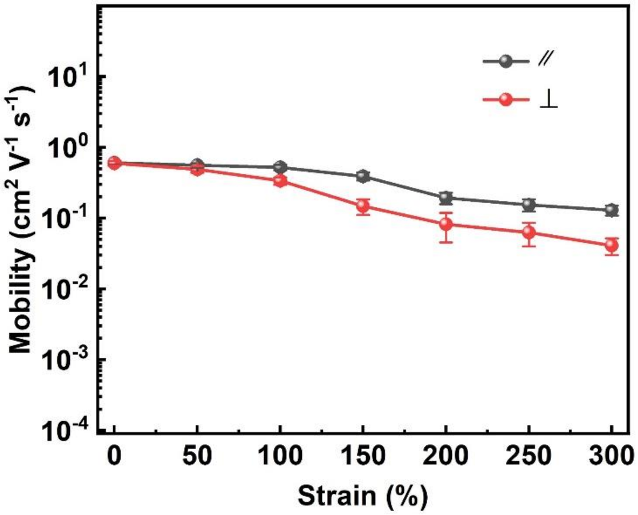

Figure S42. **Electrical characteristics of P3 films at strains**. Average mobility of polymer P3 during stretching in the stretchable transistor configuration, with charge transport parallel and perpendicular to the strain direction. All mobility values are averaged and extracted from at least ten devices.

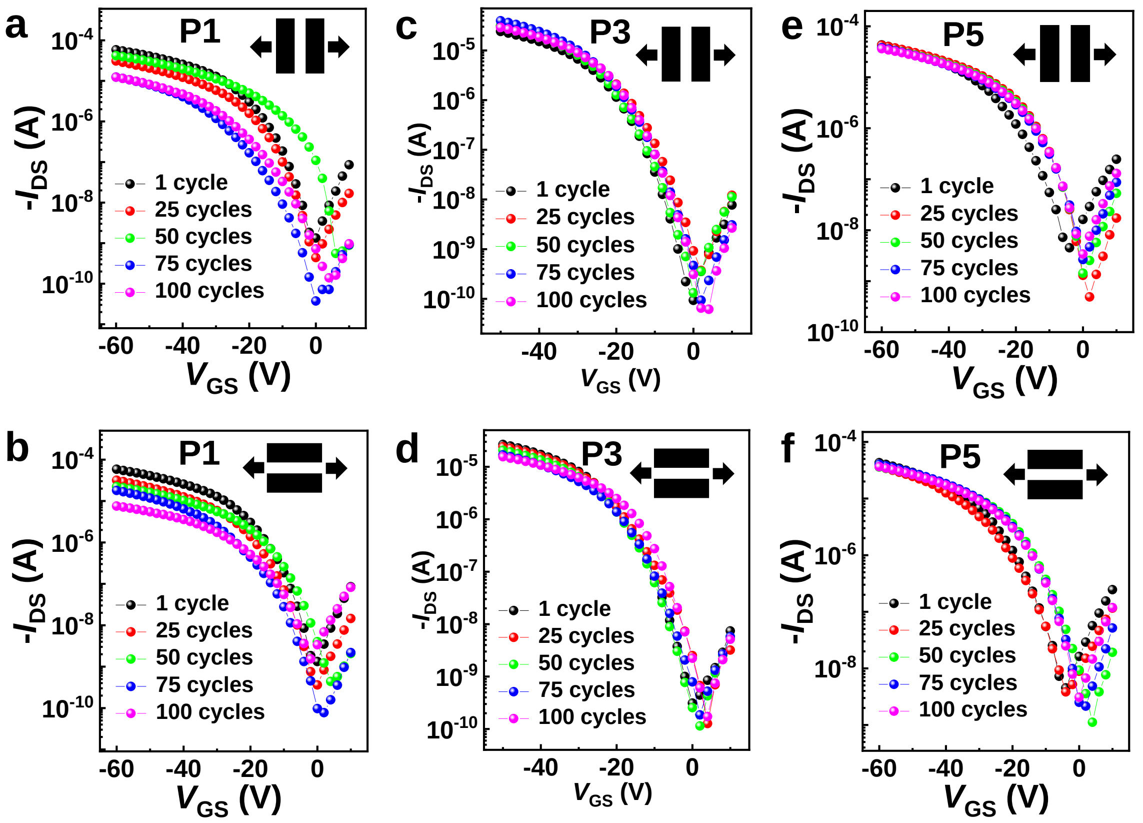

Figure S43. **Electrical characteristics of P1, P3 and P5 films under stretching-releasing cycles.** Transfer curves of P1, P3 and P5 films under 50% strain after multiple stretching-releasing cycles along the parallel (**a**, **c**, **e**) or perpendicular (**b**, **d**, **f**) stretching direction.

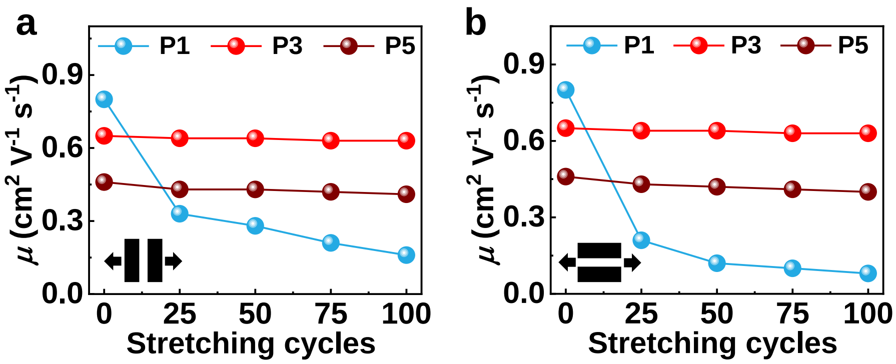

Figure S44. **Field-effect mobilities versus the different stretching cycles of P1, P3 and P5 along the parallel (a) and perpendicular (b) stretching direction.**

8. Self-healing abilities characterization

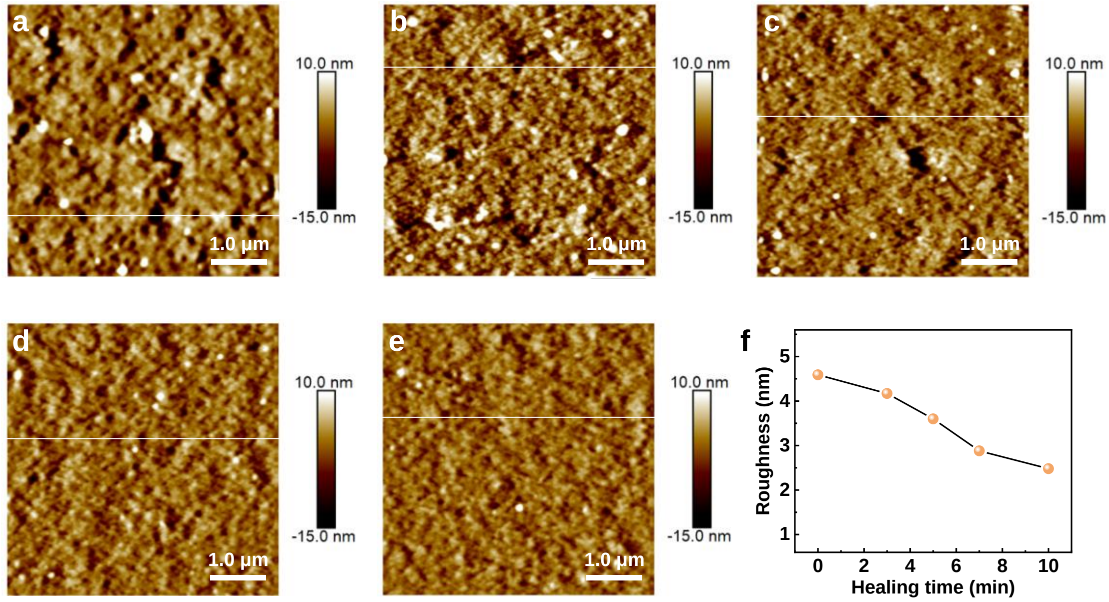

Figure S45. **Self-healing properties of P3 film under different annealing times**. AFM height images of P3 films under different annealing times for 0 min (**a**), 3 min (**b**), 5 min (**c**), 7 min (**d**), 10 min (**e**) and the corresponding roughness changes (**f**).

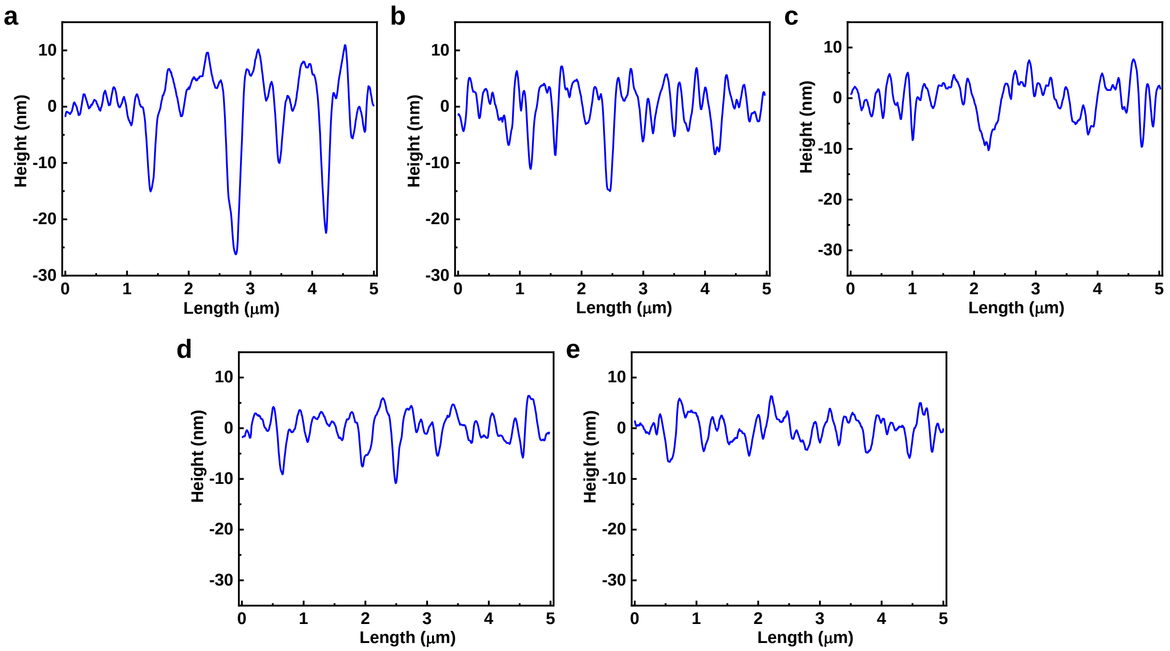

Figure S46. **The variation of P3 film thickness under different solvent annealing times for 0 min (a), 3 min (b), 5 min (c), 7 min (d), and 10 min (e)**.

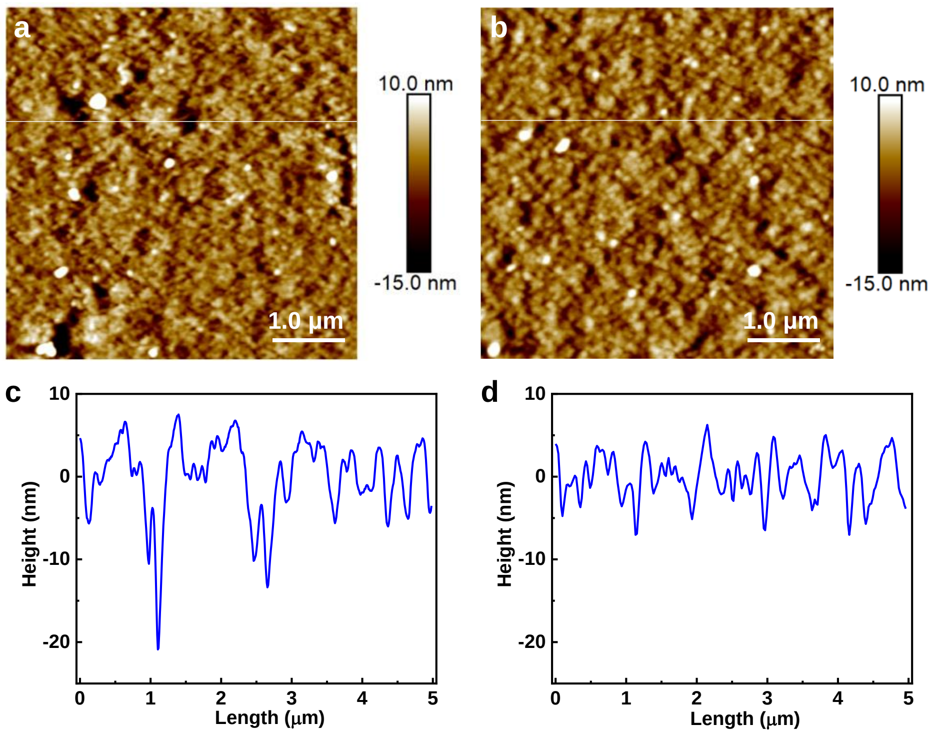

Figure S47. **AFM height images of the P3 film in the damaged state (a) and healed state at 80 °C (b). The variation of P3 film thickness in the damaged state (c) and healed state at 80 °C (d).**

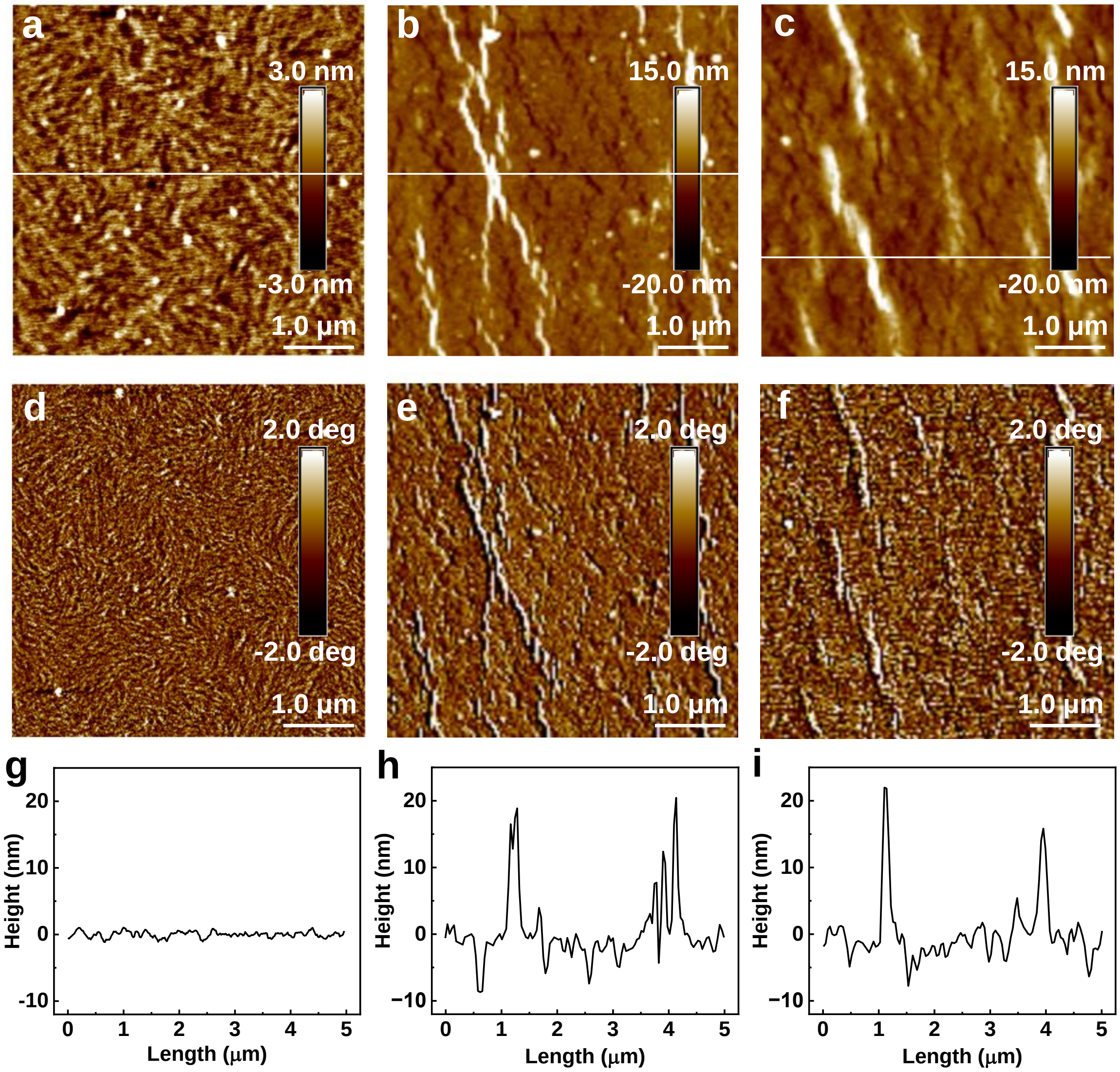

Figure S48. **Self-healing properties of P1 film**. Height image for pristine (**a**), damaged (**b**) and healed (**c**) films for P1. Phase image for pristine (**d**), damaged (**e**) and healed (**f**) films for P1. Thickness for pristine (**g**), damaged (**h**) and healed (**i**) films for P1.

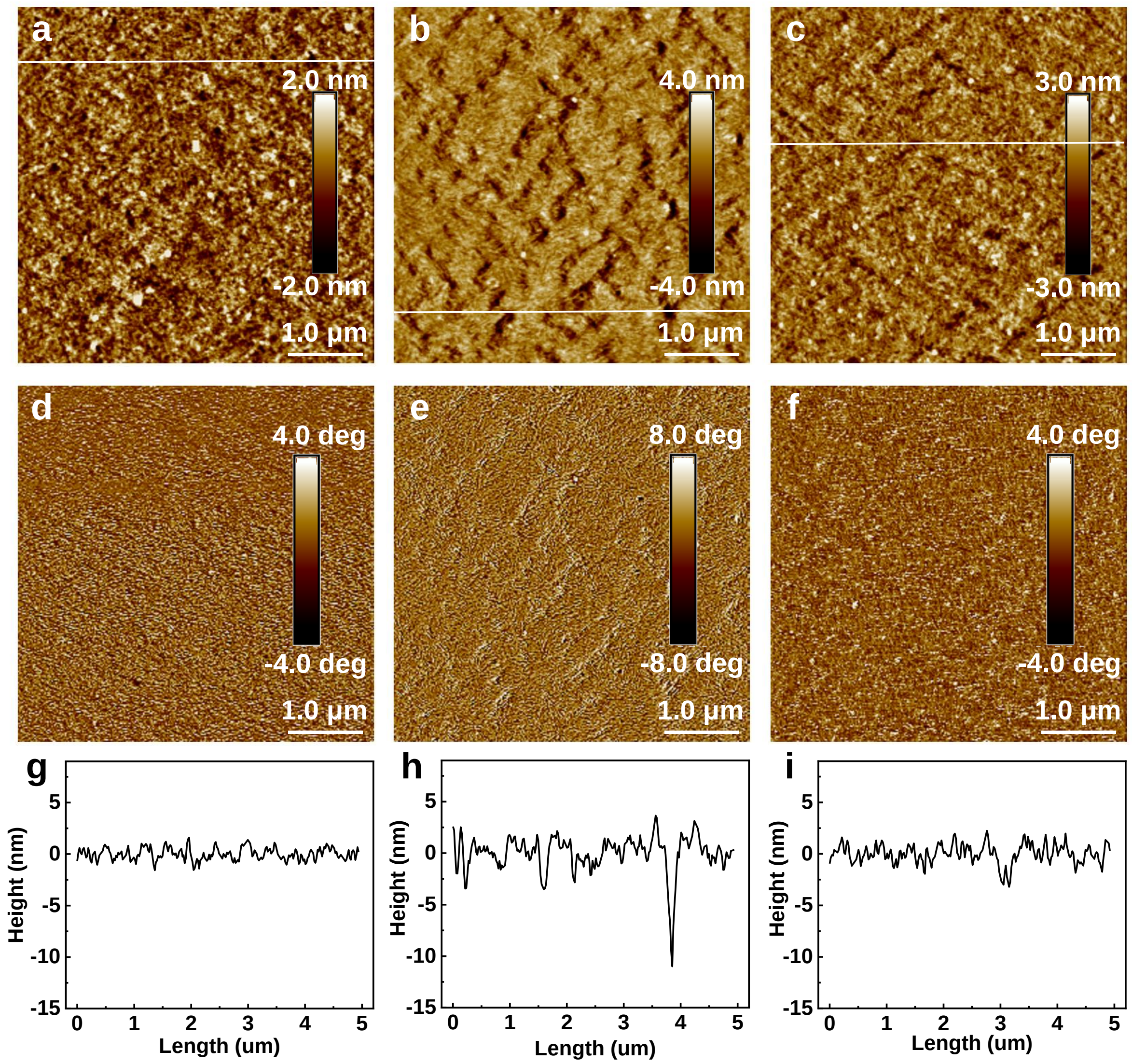

Figure S49. **Self-healing properties of P3 film**. Height image for pristine (**a**), damaged (**b**) and healed (**c**) films for P3. Phase image for pristine (**d**), damaged (**e**) and healed (**f**) films for P3. Thickness for pristine (**g**), damaged (**h**) and healed (**i**) films for P3.

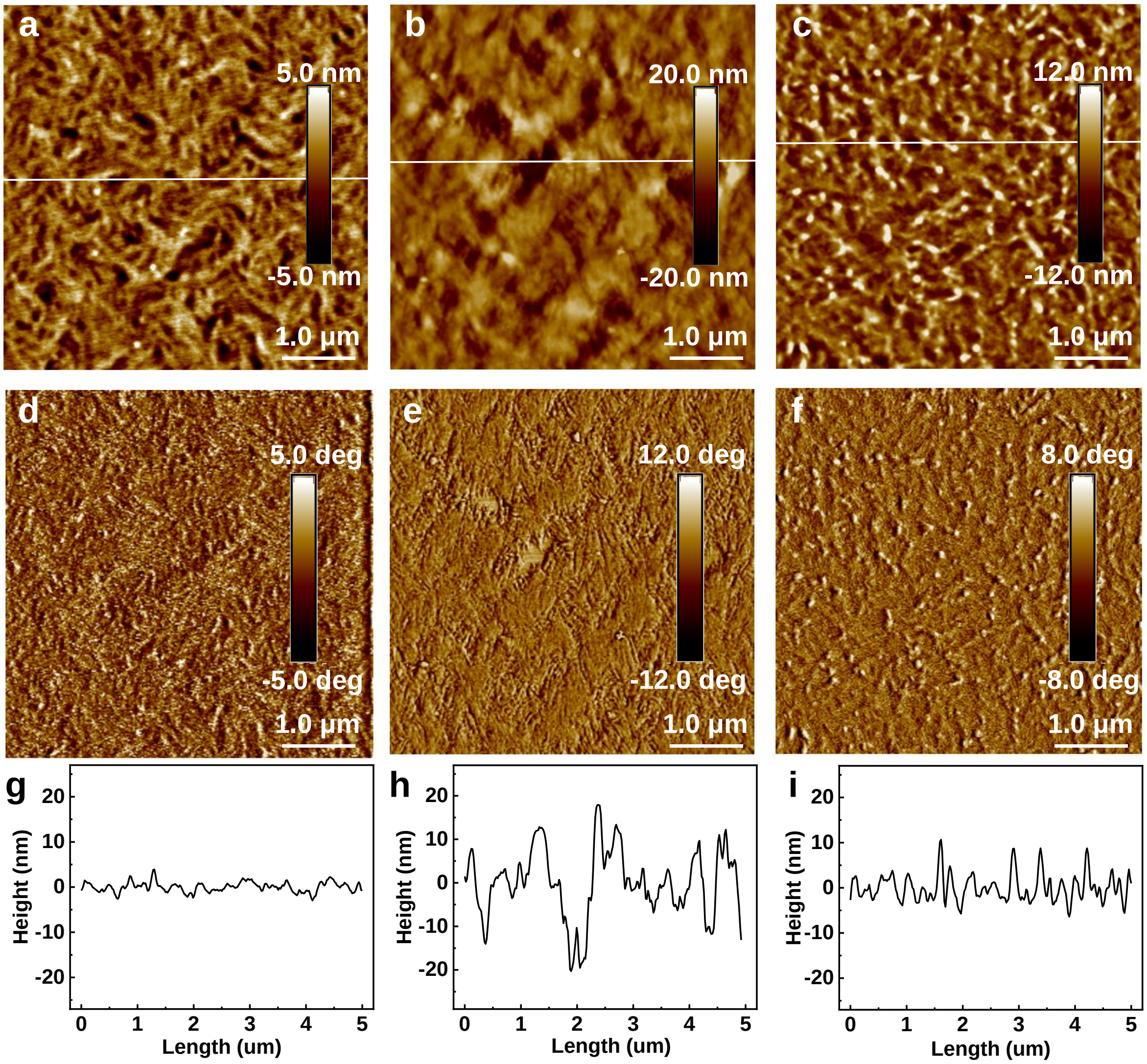

Figure S50. **Self-healing properties of P5 film**. Height image for pristine (**a**), damaged (**b**) and healed (**c**) films for P5. Phase image for pristine (**d**), damaged (**e**) and healed (**f**) films for P5. Thickness for pristine (**g**), damaged (**h**) and healed (**i**) films for P5.

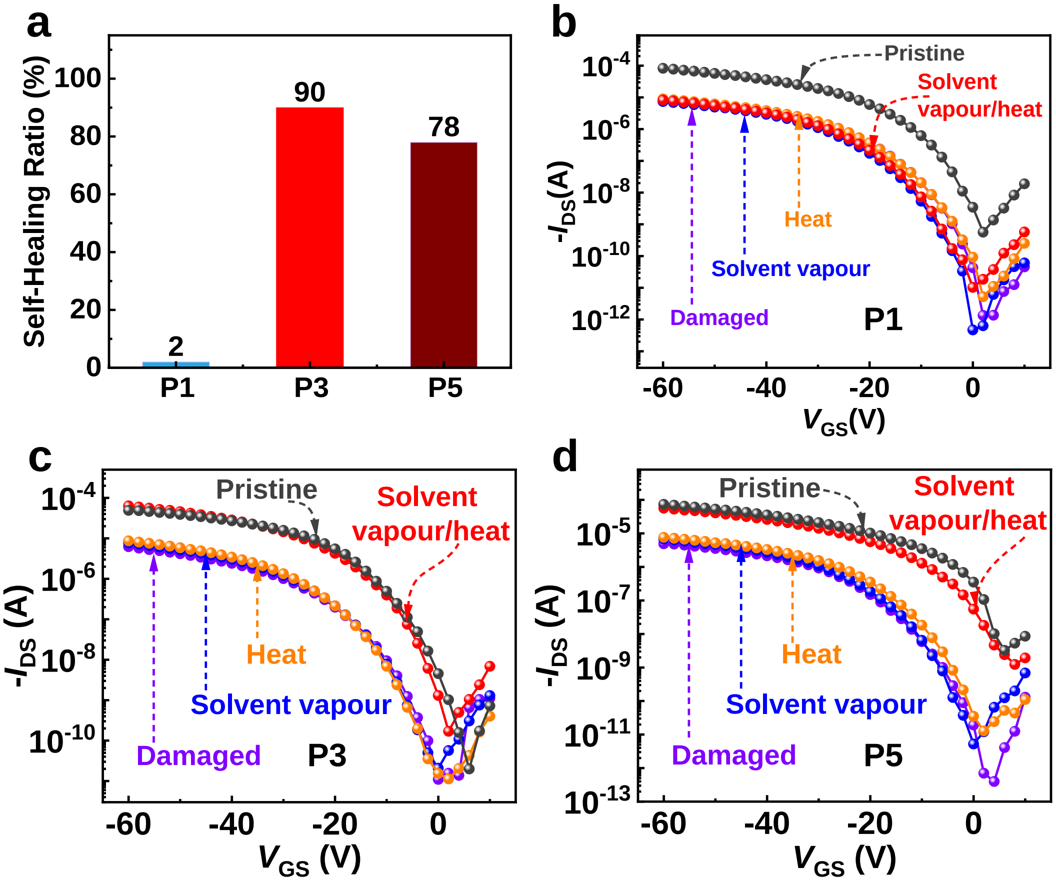

Figure S51. The self-healing ratios (a) for P1–P3 and the transfer curves of pristine, damaged, post-processing process and healed for P1 (b), P3 (c) and P5 (d).

9. Characterization of fully stretchable transistors

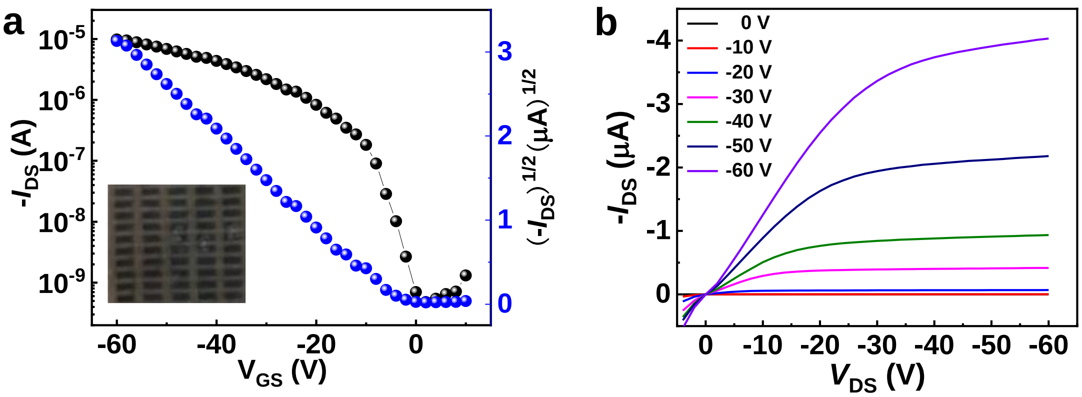

Figure S52. Transfer (a) and output (b) curves for fully stretchable transistor based on P3.

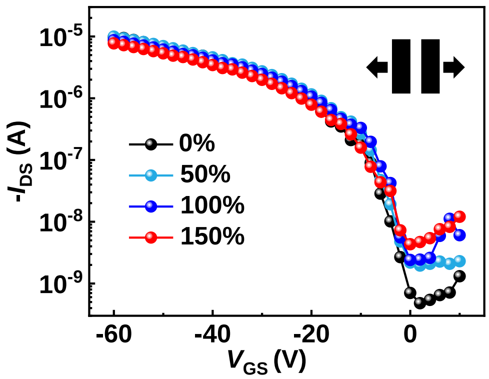

Figure S53. Transfer curves of P3 with various strains parallel to the charge transport direction.

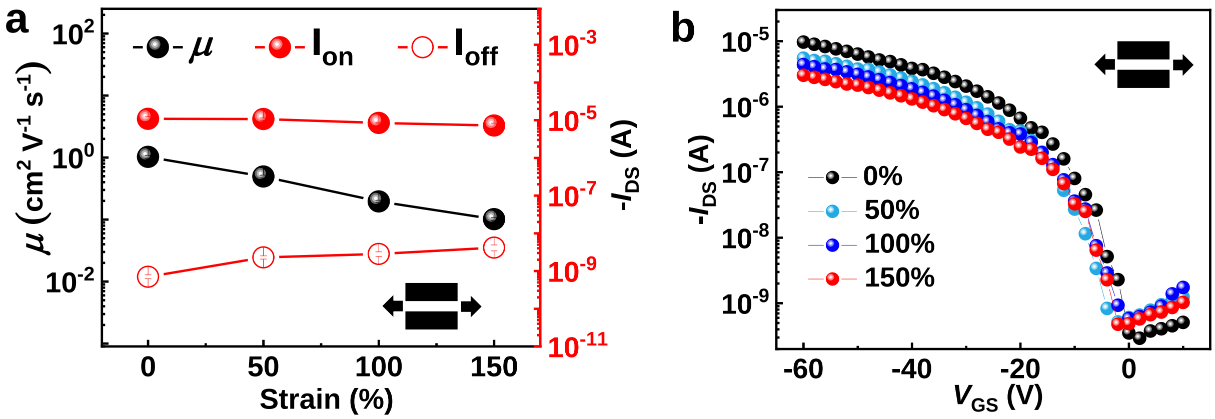

Figure S54. Electrical characteristics of P3 films with various strains for fully stretchable transistors. (**a**) Field-effect mobilities, on-currents and off-currents as a function of various strains perpendicular to the charge transport direction. (**b**) Transfer curves of P3 with various strains perpendicular to the charge transport direction.

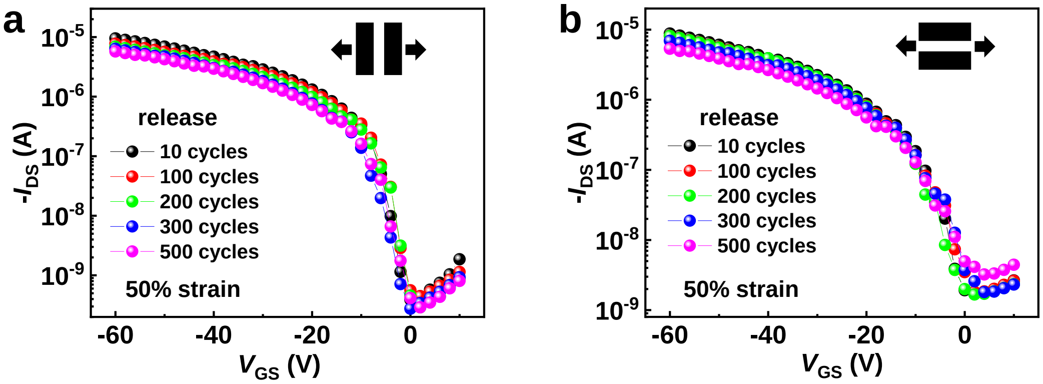

Figure S55. **Electrical characteristics of P3 films with various stretching-releasing cycles for fully stretchable transistors.** Transfer curves of the fully stretchable transistor for the releasing P3 after multiple stretching cycles along the charge transport direction (**a**) and perpendicular to charge transport direction (**b**) under 50% strain.

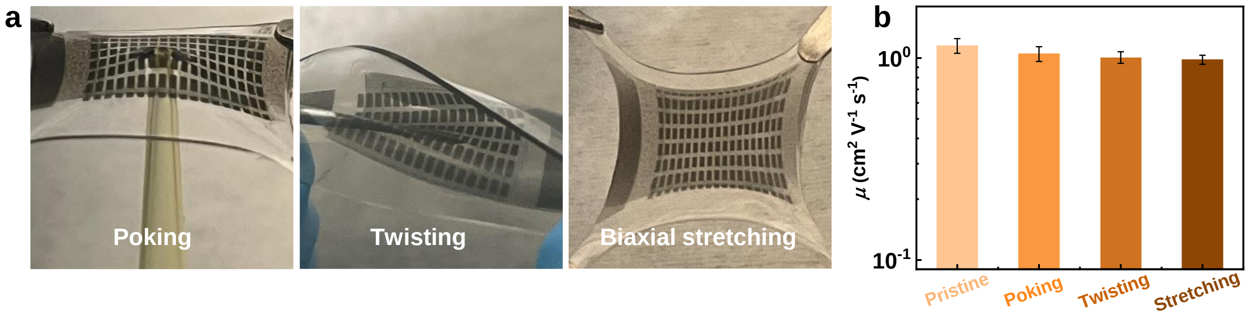

Figure S56. **Electrical stability for transistor array under various mechanical deformations**. (**a**) Photos of the fully stretchable transistors under various mechanical deformations. (**b**) Mobilities of the fully stretchable transistors after the mechanical deformations.

Table S9. Device geometry and dielectric capacitance change as function of strain in the fully stretchable transistor.

Stretching direction	Strain (%)	Channel-length (μm)	Channel-width (μm)	Capacitance (nF/cm^2)
Parallel //	0	200	1000	1.13
	25	250	930	1.45
	50	300	860	1.57
	75	350	790	1.68
	100	400	720	1.85
	150	500	580	2.15
Vertical ⊥	0	200	1000	1.13
	25	186	1250	1.45
	50	172	1500	1.57
	75	158	1750	1.68
	100	144	2000	1.85
	150	116	2500	2.15

10. NMR Spectra

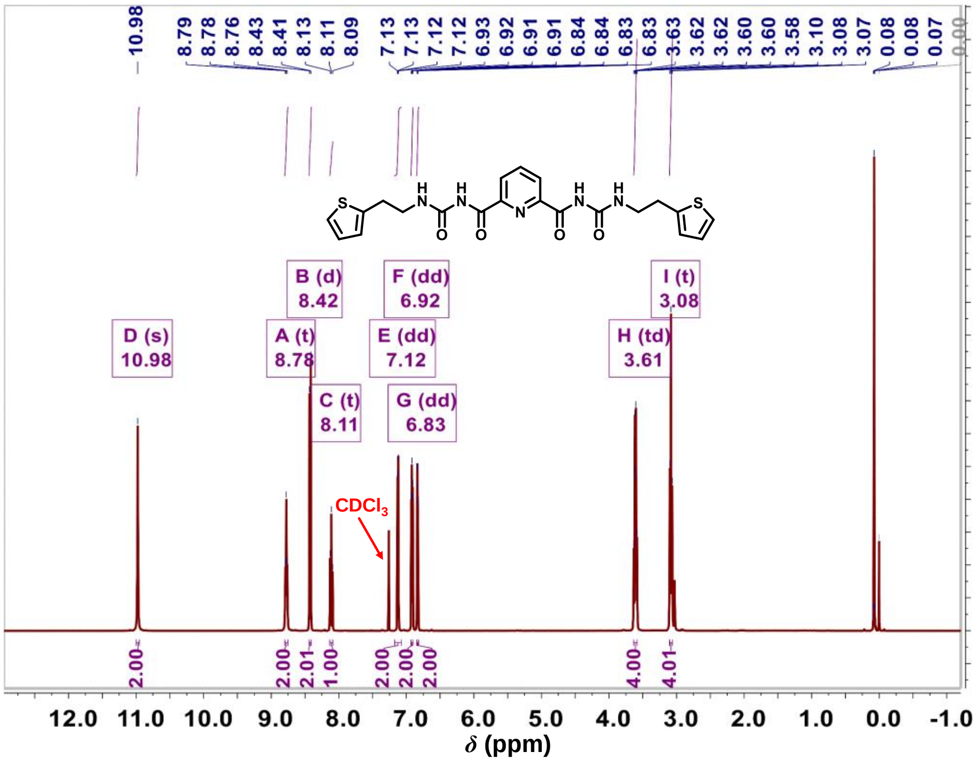

^{1}H NMR spectrum of DCPDCA in $CDCl_3$.

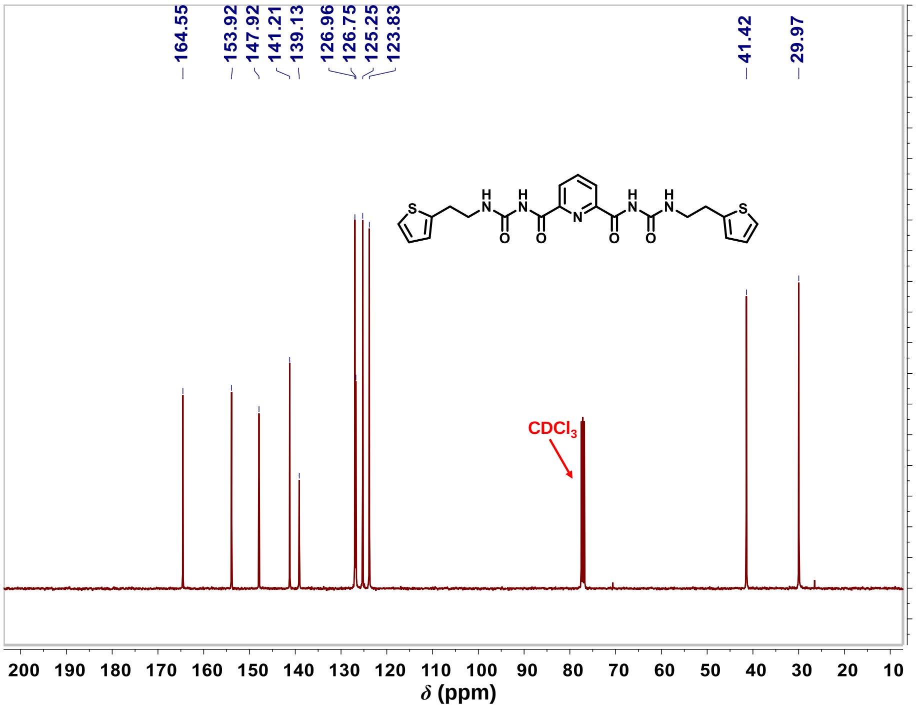

^{13}C NMR spectrum of DCPDCA in $CDCl_3$.

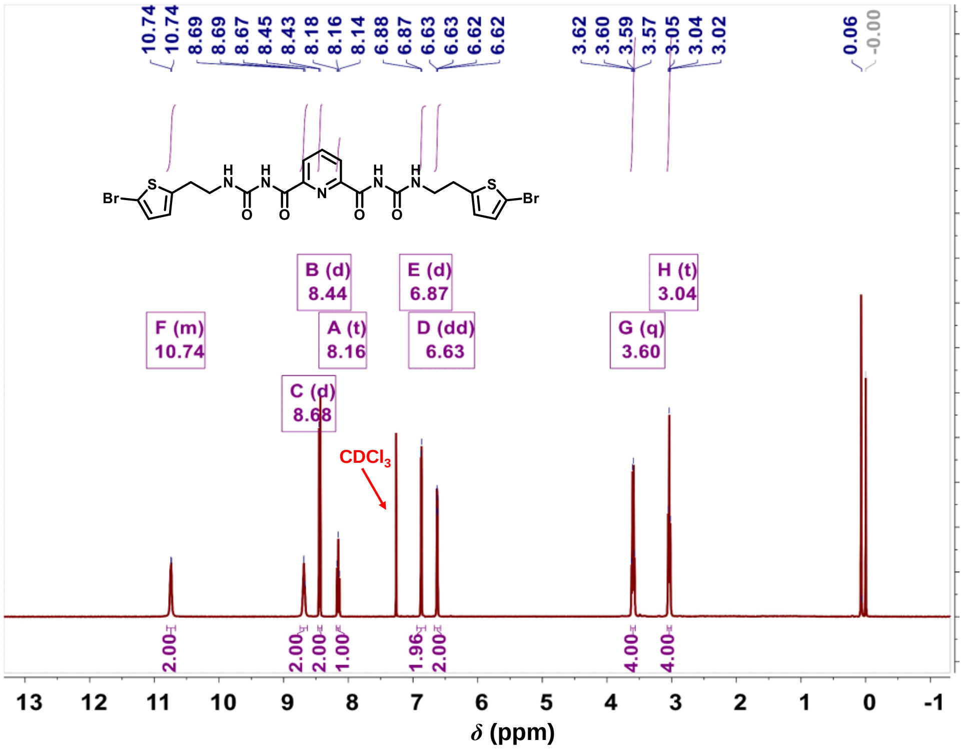

^{1}H NMR spectrum of compound DCPDCA-Br2 in CDCl3.

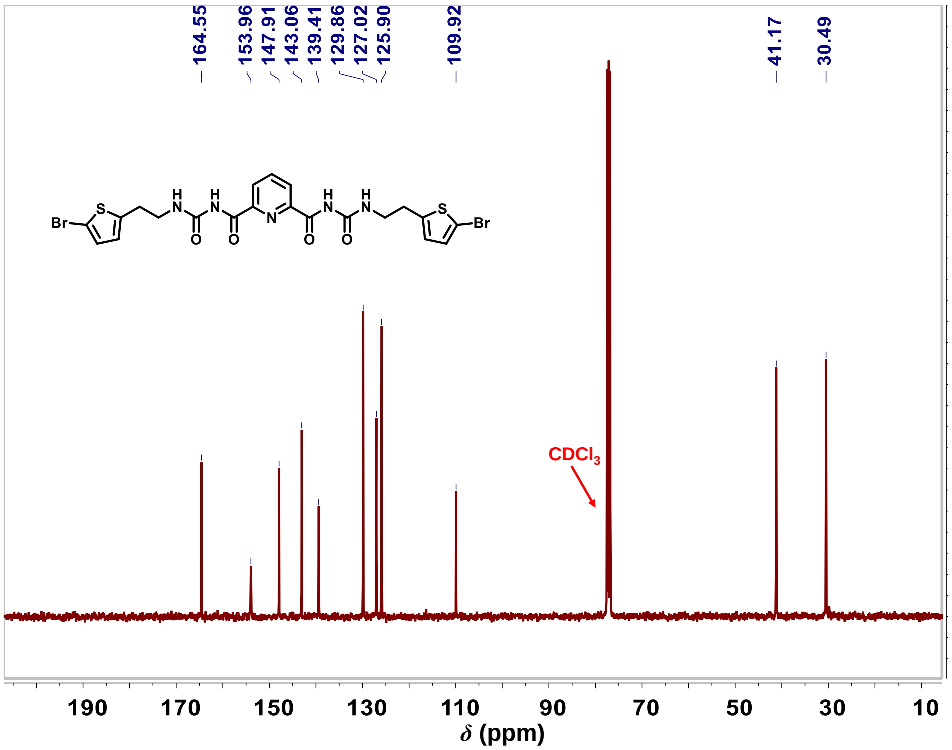

^{13}C NMR spectrum of DCPDCA-Br2 in CDCl3.

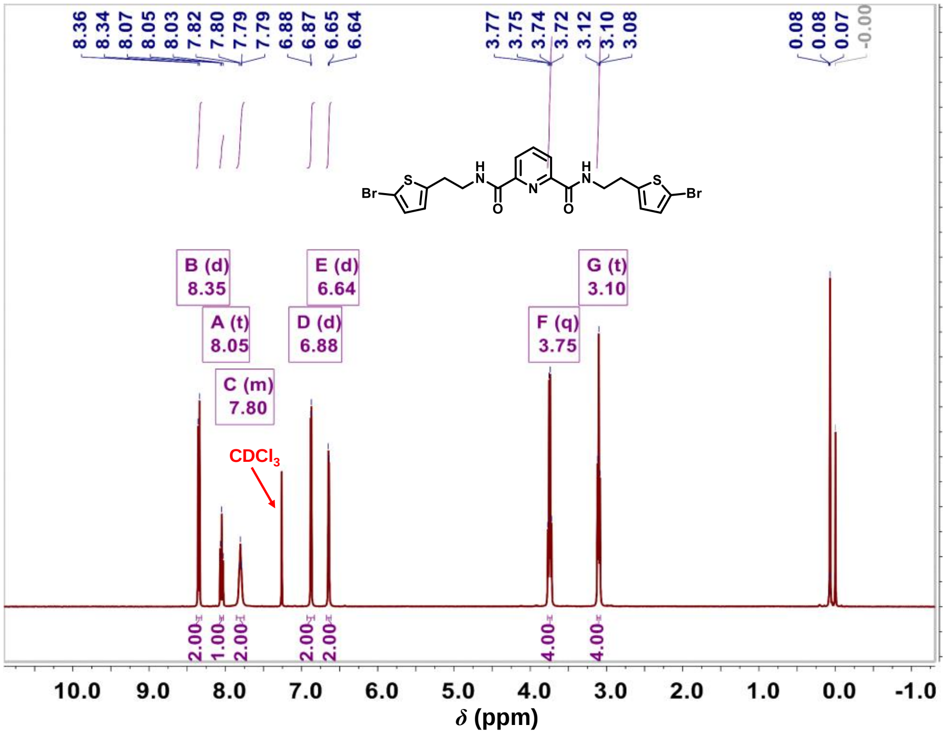

^{1}H NMR spectrum of PDCA-Br$_2$ in CDCl$_3$.

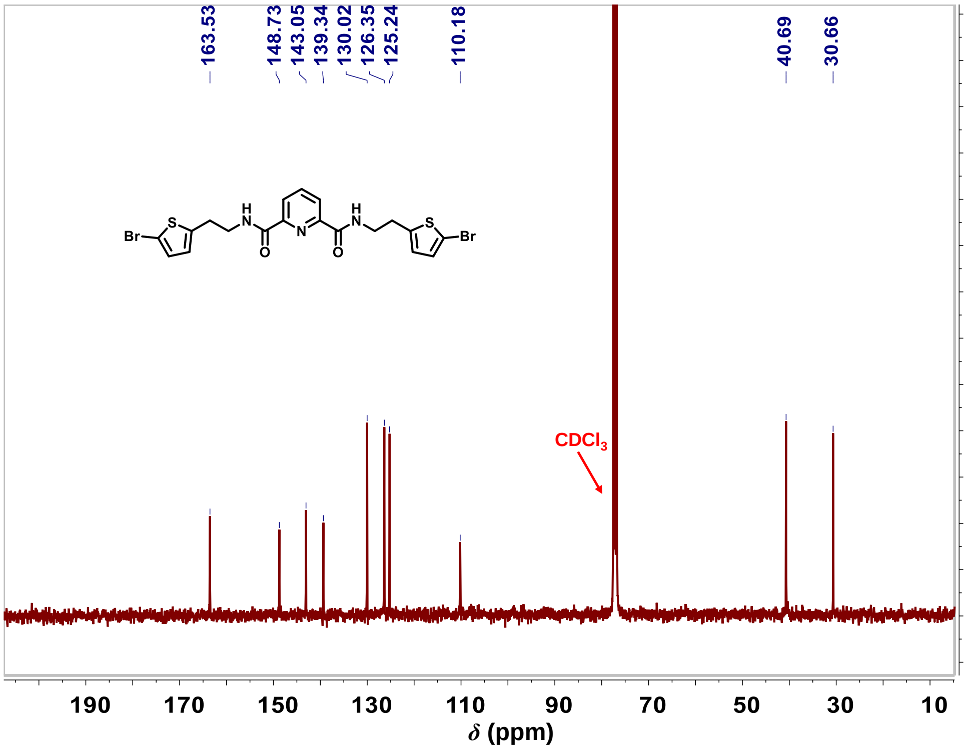

^{13}C NMR spectrum of PDCA-Br$_2$ in CDCl$_3$.

11. References

1. Oh J, Rondeau G, Chiu Y *et al*. Intrinsically stretchable and healable semiconducting polymer for organic transistors. *Nature* 2016; **539**: 411-415.

checkCIF/PLATON report

Structure factors have been supplied for datablock(s) tx16599_auto

THIS REPORT IS FOR GUIDANCE ONLY. IF USED AS PART OF A REVIEW PROCEDURE FOR PUBLICATION, IT SHOULD NOT REPLACE THE EXPERTISE OF AN EXPERIENCED CRYSTALLOGRAPHIC REFEREE.

No syntax errors found.   CIF dictionary   Interpreting this report

Datablock: tx16599_auto

```
Bond precision:   C-C = 0.0076 A          Wavelength=1.54184

Cell:            a=9.6407(4)        b=15.5622(5)      c=16.1443(4)
                 alpha=65.352(3)    beta=80.626(3)    gamma=79.511(3)
Temperature:     170 K

                  Calculated                      Reported
Volume            2154.39(14)                     2154.41(14)
Space group       P -1                            P -1
Hall group        -P 1                            -P 1
Moiety formula    C21 H21 N5 O4 S2                4(C21 H21 N5 O4 S2)
Sum formula       C21 H21 N5 O4 S2                C84 H84 N20 O16 S8
Mr                471.55                          1886.19
Dx,g cm-3         1.454                           1.454
Z                 4                               1
Mu (mm-1)         2.585                           2.585
F000              984.0                           984.0
F000'             989.42
h,k,lmax          12,19,20                        12,19,20
Nref              9052                            8629
Tmin,Tmax                                         0.759,1.000
Tmin'

Correction method= # Reported T Limits: Tmin=0.759 Tmax=1.000
AbsCorr = MULTI-SCAN

Data completeness= 0.953           Theta(max)= 76.570

                                                    wR2(reflections)=
R(reflections)= 0.0732( 7405)                       0.2209( 8629)

S = 1.054                  Npar= 669
```

The following ALERTS were generated. Each ALERT has the format
test-name_ALERT_alert-type_alert-level.
Click on the hyperlinks for more details of the test.

Alert level C

```
PLAT041_ALERT_1_C Calc. and Reported SumFormula    Strings  Differ     Please Check
              Calc: C21 H21 N5 O4 S2
              Rep.: C84 H84 N20 O16 S8
PLAT042_ALERT_1_C Calc. and Reported MoietyFormula Strings  Differ     Please Check
              Calc: C21 H21 N5 O4 S2
              Rep.: 4(C21 H21 N5 O4 S2)
PLAT053_ALERT_1_C Minimum Crystal Dimension Missing (or Error) ...     Please Check
PLAT054_ALERT_1_C Medium  Crystal Dimension Missing (or Error) ...     Please Check
PLAT055_ALERT_1_C Maximum Crystal Dimension Missing (or Error) ...     Please Check
PLAT213_ALERT_2_C Atom C23             has ADP max/min Ratio .....        3.3 prolat
PLAT220_ALERT_2_C NonSolvent   Resd 1  C   Ueq(max)/Ueq(min) Range        3.9 Ratio
PLAT220_ALERT_2_C NonSolvent   Resd 2  C   Ueq(max)/Ueq(min) Range        3.7 Ratio
PLAT340_ALERT_3_C Low Bond Precision on  C-C Bonds ..............    0.00763 Ang.
PLAT767_ALERT_4_C INS Embedded LIST 6 Instruction Should be LIST 4     Please Check
PLAT906_ALERT_3_C Large K Value in the Analysis of Variance ......      7.869 Check
PLAT906_ALERT_3_C Large K Value in the Analysis of Variance ......      2.253 Check
PLAT911_ALERT_3_C Missing FCF Refl Between Thmin & STh/L=    0.600         28 Report
               -4  1  0,   5  1  0,  -3  6  0,  -7-14  1,   8 14  1,   7 15  1,
                2  6  2,   2  9  2,   9 13  2,  -5-14  3,  -3-10  3,  -5-13  5,
                5 -4  5,   1 -3  5,  -3  4  5,  -4  5  5,  -9  8  6,   3  3  7,
                7  5  7,  -2-12  8,  -2-11  9,   2 12  9,   3  1 12,  -1  5 12,
                3 10 12,   0 -6 14,   0 -3 16,  -1  0 17,
PLAT975_ALERT_2_C Check Calcd Resid. Dens.  0.94Ang From O4      .       0.47 eA-3
PLAT975_ALERT_2_C Check Calcd Resid. Dens.  1.00Ang From O7      .       0.40 eA-3
PLAT977_ALERT_2_C Check Negative Difference Density on H19       .      -0.31 eA-3
```

Alert level G

```
PLAT002_ALERT_2_G Number of Distance or Angle Restraints on AtSite         22 Note
PLAT003_ALERT_2_G Number of Uiso or U(i,j)  Restrained non-H-Atoms         20 Report
PLAT007_ALERT_5_G Number of Unrefined Donor-H Atoms .............          8 Report
              H1    H2    H4    H5    H6    H7    H9    H10A
PLAT045_ALERT_1_G Calculated and Reported Z Differ by a Factor ...          4 Check
PLAT083_ALERT_2_G SHELXL Second Parameter in WGHT  Unusually Large      10.03 Why ?
PLAT154_ALERT_1_G The s.u.'s on the Cell Angles are Equal ..(Note)      0.003 Degree
PLAT172_ALERT_4_G The CIF-Embedded .res File Contains DFIX Records          5 Report
PLAT176_ALERT_4_G The CIF-Embedded .res File Contains SADI Records         26 Report
PLAT178_ALERT_4_G The CIF-Embedded .res File Contains SIMU Records          3 Report
PLAT186_ALERT_4_G The CIF-Embedded .res File Contains ISOR Records          5 Report
PLAT188_ALERT_3_G A Non-default SIMU Restraint Value has been used     0.0100 Report
PLAT188_ALERT_3_G A Non-default SIMU Restraint Value has been used     0.0100 Report
PLAT188_ALERT_3_G A Non-default SIMU Restraint Value has been used     0.0200 Report
PLAT191_ALERT_3_G A Non-default SADI Restraint Value has been used     0.0400 Report
PLAT191_ALERT_3_G A Non-default SADI Restraint Value has been used     0.0400 Report
PLAT191_ALERT_3_G A Non-default SADI Restraint Value has been used     0.0400 Report
PLAT191_ALERT_3_G A Non-default SADI Restraint Value has been used     0.0400 Report
PLAT191_ALERT_3_G A Non-default SADI Restraint Value has been used     0.0400 Report
PLAT191_ALERT_3_G A Non-default SADI Restraint Value has been used     0.0400 Report
PLAT191_ALERT_3_G A Non-default SADI Restraint Value has been used     0.0400 Report
PLAT191_ALERT_3_G A Non-default SADI Restraint Value has been used     0.0400 Report
```

```
PLAT191_ALERT_3_G A Non-default SADI Restraint Value has been used     0.0400 Report
PLAT191_ALERT_3_G A Non-default SADI Restraint Value has been used     0.0400 Report
PLAT191_ALERT_3_G A Non-default SADI Restraint Value has been used     0.0400 Report
PLAT191_ALERT_3_G A Non-default SADI Restraint Value has been used     0.0400 Report
PLAT191_ALERT_3_G A Non-default SADI Restraint Value has been used     0.0400 Report
PLAT191_ALERT_3_G A Non-default SADI Restraint Value has been used     0.0400 Report
PLAT301_ALERT_3_G Main Residue  Disorder .............(Resd    1)        31% Note
PLAT432_ALERT_2_G Short Inter X...Y Contact  S19      ..C35      .       3.21 Ang.
                                                   x,y,z  =      1_555 Check
PLAT432_ALERT_2_G Short Inter X...Y Contact  C40      ..C40      .       3.18 Ang.
                                               -x,-y,1-z  =      2_556 Check
PLAT720_ALERT_4_G Number of Unusual/Non-Standard Labels ..........          6 Note
              H5AA    H5AB    H5BC    H5BD    H2AA    H4AA
PLAT860_ALERT_3_G Number of Least-Squares Restraints .............        338 Note
PLAT912_ALERT_4_G Missing # of FCF Reflections Above STh/L=  0.600        395 Note
PLAT933_ALERT_2_G Number of HKL-OMIT Records in Embedded .res File         17 Note
               -5-14  3,  -4  1  0,  -4  5  5,  -3-10  3,  -3  4  5,  -3  6  0,
               -1  5 12,   1 -3  5,   2  6  2,   2  9  2,   2 12  9,   3  1 12,
                3  3  7,   3 10 12,   5 -4  5,   5  1  0,   7  5  7,
PLAT941_ALERT_3_G Average HKL Measurement Multiplicity ..........        3.2 Low
PLAT969_ALERT_5_G The 'Henn et al.' R-Factor-gap value ..........      4.798 Note
              Predicted wR2: Based on SigI**2  4.60 or SHELX Weight 20.95
PLAT978_ALERT_2_G Number C-C Bonds with Positive Residual Density.          1 Info
```

---

0 **ALERT level A** = Most likely a serious problem - resolve or explain
0 **ALERT level B** = A potentially serious problem, consider carefully
16 **ALERT level C** = Check. Ensure it is not caused by an omission or oversight
37 **ALERT level G** = General information/check it is not something unexpected

7 ALERT type 1 CIF construction/syntax error, inconsistent or missing data
13 ALERT type 2 Indicator that the structure model may be wrong or deficient
24 ALERT type 3 Indicator that the structure quality may be low
7 ALERT type 4 Improvement, methodology, query or suggestion
2 ALERT type 5 Informative message, check

It is advisable to attempt to resolve as many as possible of the alerts in all categories. Often the minor alerts point to easily fixed oversights, errors and omissions in your CIF or refinement strategy, so attention to these fine details can be worthwhile. In order to resolve some of the more serious problems it may be necessary to carry out additional measurements or structure refinements. However, the purpose of your study may justify the reported deviations and the more serious of these should normally be commented upon in the discussion or experimental section of a paper or in the "special_details" fields of the CIF. checkCIF was carefully designed to identify outliers and unusual parameters, but every test has its limitations and alerts that are not important in a particular case may appear. Conversely, the absence of alerts does not guarantee there are no aspects of the results needing attention. It is up to the individual to critically assess their own results and, if necessary, seek expert advice.

Publication of your CIF in IUCr journals

A basic structural check has been run on your CIF. These basic checks will be run on all CIFs submitted for publication in IUCr journals (*Acta Crystallographica*, *Journal of Applied Crystallography*, *Journal of Synchrotron Radiation*); however, if you intend to submit to *Acta Crystallographica Section C* or *E* or *IUCrData*, you should make sure that full publication checks are run on the final version of your CIF prior to submission.

Publication of your CIF in other journals

Please refer to the *Notes for Authors* of the relevant journal for any special instructions relating to CIF submission.

PLATON version of 19/12/2024; check.def file version of 19/12/2024

Datablock tx16599_auto - ellipsoid plot

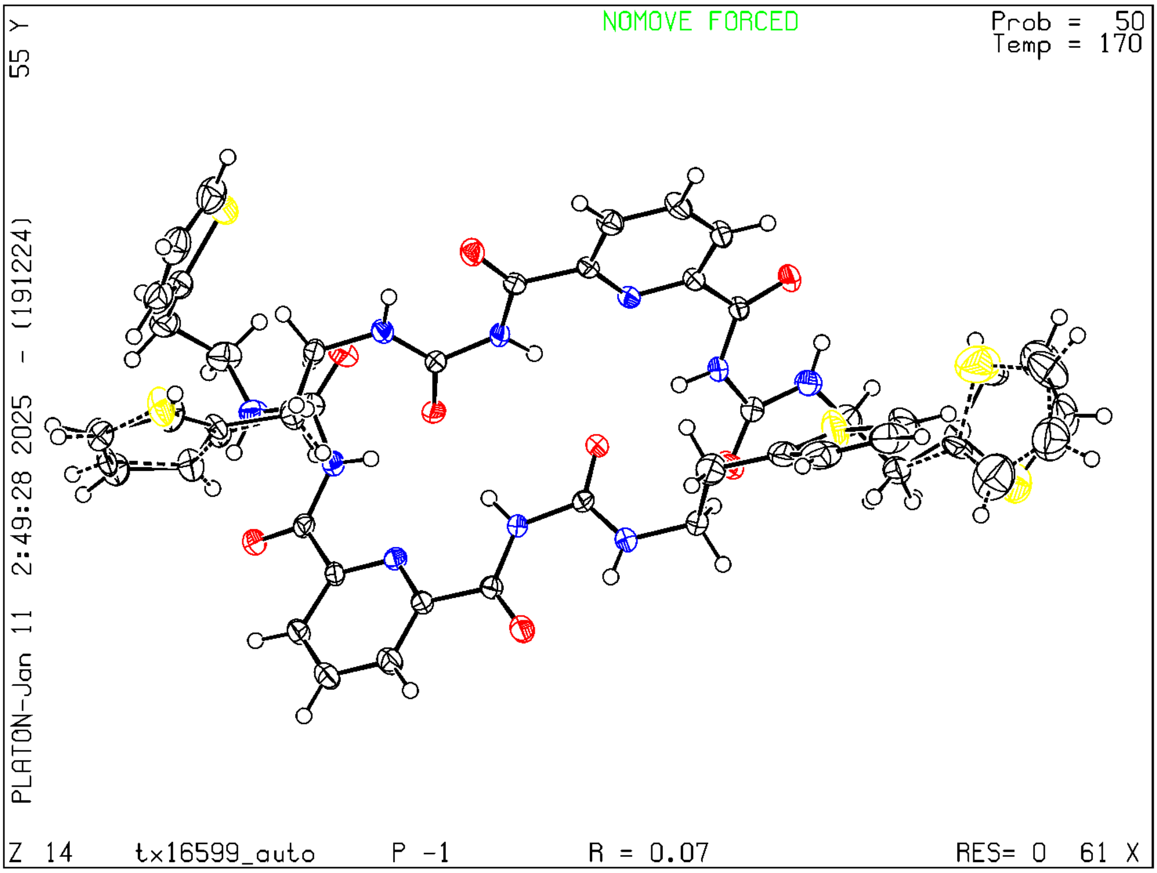

Supplement: nwag162_Supplemental_File [file nwag162_supplemental_file.pdf]
